# Steric “attraction”: not by dispersion alone

**DOI:** 10.3762/bjoc.14.125

**Published:** 2018-06-19

**Authors:** Ganna Gryn’ova, Clémence Corminboeuf

**Affiliations:** 1 Institut des Sciences et Ingénierie Chimiques, École polytechnique fédérale de Lausanne, CH-1015 Lausanne, Switzerland

**Keywords:** charge penetration, dispersion, hydrocarbon, non-covalent interactions, steric attraction

## Abstract

Non-covalent interactions between neutral, sterically hindered organic molecules generally involve a strong stabilizing contribution from dispersion forces that in many systems turns the ‘steric repulsion’ into a ‘steric attraction’. In addition to London dispersion, such systems benefit from electrostatic stabilization, which arises from a short-range effect of charge penetration and gets bigger with increasing steric bulk. In the present work, we quantify this contribution for a diverse set of molecular cores, ranging from unsubstituted benzene and cyclohexane to their derivatives carrying *tert*-butyl, phenyl, cyclohexyl and adamantyl substituents. While the importance of electrostatic interactions in the dimers of sp^2^-rich (e.g., π-conjugated) cores is well appreciated, less polarizable assemblies of sp^3^-rich systems with multiple short-range CH···HC contacts between the bulky cyclohexyl and adamantyl moieties are also significantly influenced by electrostatics. Charge penetration is drastically larger in absolute terms for the sp^2^-rich cores, but still has a non-negligible effect on the sp^3^-rich dimers, investigated herein, both in terms of their energetics and equilibrium interaction distances. These results emphasize the importance of this electrostatic effect, which has so far been less recognized in aliphatic systems compared to London dispersion, and are therefore likely to have implications for the development of force fields and methods for crystal structure prediction.

## Introduction

**Figure 1:**
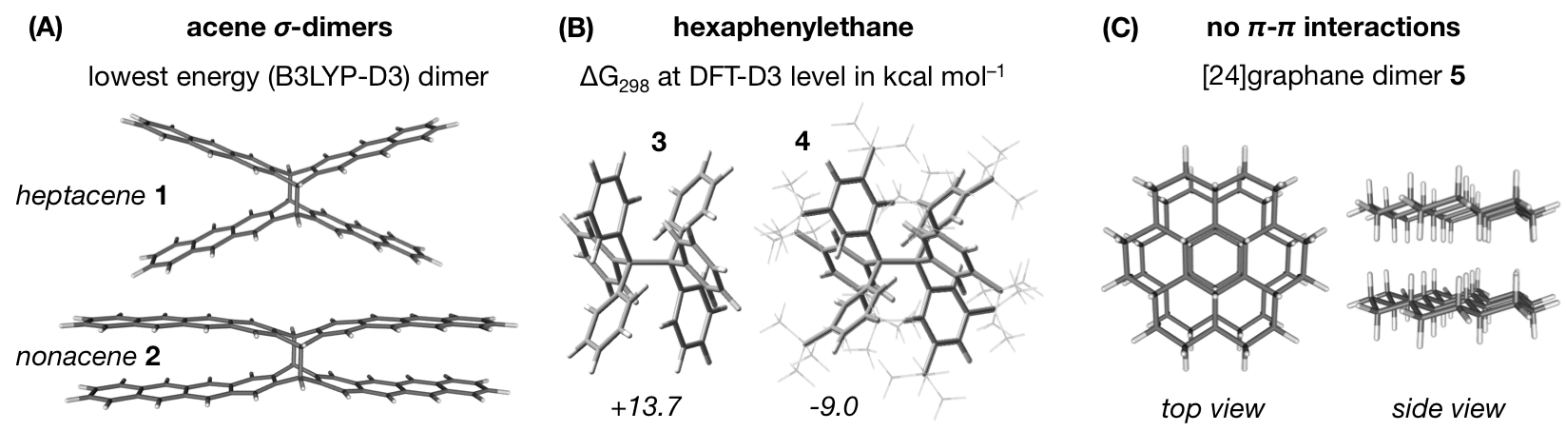
(A) Dispersion is insufficient to bend the heptacene σ-dimer, but becomes sizable enough in nonacene [2]. (B) Bare hexaphenylethane is not thermodynamically stable, but its all-*meta*-*tert*-butyl derivative is [3–4]. (C) Thermodynamically stable (at B97D/6-31G(d,p) level) bilayer of a fully saturated hydrocarbon, [24]-graphane [15].

In the recent years, perception of the vaguely defined ‘steric’ interactions as categorically repulsive has shifted towards recognizing the crucial role of attractive dispersion in the bulky systems [1]. London dispersion was shown to be capable of bending σ-bonded acene dimers (**2** in Figure 1A) [2] and stabilizing extremely crowded systems, hexaphenylethane (**3**) being the mascot of this concept. As elegantly illustrated by Schreiner and Grimme [3–4], while bare hexaphenylethane (**3**) is thermodynamically unstable due to significant Pauli repulsion between the phenyl rings, its analogue **4** carrying all-*meta*-*tert*-butyl substituents (termed ‘dispersion donors’) can be synthesized and characterized thanks to sufficient stabilization by dispersion (Figure 1B). This realization sparked a race towards the longest covalent C–C bonds [5–6]: already impressive 1.67 Å in hexakis(3,5-di-*tert*-butylphenyl)ethane is not even a limit and stable diamondoid dimer with a central C–C bond as long as 1.71 Å has been achieved [7–8]. Bulky alkyl groups assist not only in achieving the longest C–C bonds, but also the shortest intermolecular H···H contacts [9], which are otherwise tackled by squeezing them inside the cages [10]. Intermolecular interactions in hydrocarbons are also subject to significant dispersion contribution. In the unsaturated systems, from benzene dimer to higher acenes and, ultimately, graphenes, dispersion is increasingly the key force behind the π–π stacking interactions [11]. Large and flat π-conjugated moieties (e.g., ligands) are even referred to as ‘sticky pancakes’ in homage to strong attractive interactions between them [12–14]. Less intuitively, similarly strong attractive forces are found in extended saturated systems, e.g., the double sheet graphanes and [*n*]ladderane dimers, where the interaction occurs via the CH···HC and CH···C contacts (Figure 1C) [15–24].

While London dispersion is deservedly paraded as the champion of ‘steric attraction’ in bulky hydrocarbons, several studies have recently pointed to the somewhat less expected electrostatic contribution to it [25]. For example, “dispersion dominates and electrostatics commands” is the ‘punch line’ of the 2017 computational study on the σ–σ, σ–π and π–π stacking interactions between benzene, cyclohexane and some of their fluorinated derivatives [26]. The authors show that while electrostatics is not the largest stabilizing energetic contribution, it is nonetheless the one that defines the trend in the total interaction energy for a range of investigated dimers. Electrostatic stabilization in graphane and graphene dimers has been attributed to the charge transfer (σ_CH_ → σ_HC*_ hyperconjugative interaction) [17], and a similar argument was used to suggest the possibility of manipulating the band gap of patterned hydrogenated graphene C_4_H bilayer by an external electric field [27]. Furthermore, Schreiner et al. showed that approx. 10% of the total interaction energy in the tris(3,5-di-*tert*-butylphenyl)methane dimer (the system mentioned above for its shortest intermolecular H···H contacts) comes from stabilizing electrostatics [9]. Similarly, the interaction energy difference between the all-meta-*tert*-butyl-hexaphenylethane and the bare hexaphenylethane features ≈14% electrostatic contribution at the ISAPT0/jun-cc-pVDZ level [28]. These studies have identified the penetration energy as the dominant component of the electrostatic interaction energy. While at long range electrostatics is virtually entirely due to interactions between the permanent multipoles of the interacting species, at small interaction distances it is instead strongly influenced and in some systems even dominated by charge penetration [29]. The latter is an outcome of the overlapping diffuse electron clouds of interacting molecules. The resulting attraction between the nuclei of one molecule to the electron density of the other is greater than the electron–electron and nuclei–nuclei repulsion. The crucial role of charge penetration has been demonstrated for a diverse range of chemical systems, including the saturated [9,28] and unsaturated hydrocarbons [30], nucleic acids [31], metal ions interacting with proteins [32], heteroaromatic cores that are the common building blocks for organic semiconductors [33], cyclophanes [34], Wilcox torsion balance systems [35], etc.

The recognized importance of charge penetration in various chemical problems is paralleled by myriad developments aimed at accurately describing intermolecular interactions. Effective fragment potential (EFP) methods estimate it by adding a damping term to their classical multipolar expansion [36–37]. To account for this effect, quantum mechanically derived force fields (FFs) are fitted to semi-empirical [38], dispersion-corrected density functional theory [39], post-Hartree–Fock [40–41], symmetry adapted perturbation theory (SAPT) [42–46] data or to a combination of the latter two (e.g., the monomer electron density force field, MEDFF) [47]. The latter approach has been subsequently exploited in the machine learning parameterization of physics-based potentials [48]. Explicit corrections for the missing penetration term in standard FFs were also introduced based on SAPT [49–50], a Gaussian electrostatic model (GEM), which uses density fitting to afford continuous description of molecular charge [51–52], a charge-distribution model based on a promolecule augmented with point charges [53] and a screened charge model with a molecular mechanics outer density screening algorithm [54]. In the context of hydrocarbon chemistry, the need to include charge penetration in FFs when modeling π–π and CH···π interactions in unsaturated hydrocarbons has been emphasized by Sherrill et al. in 2009 [55]. Accordingly, several potentials with accurate electrostatics treatment have been developed and successfully applied to describe the intermolecular interactions of anthracene [56], polycyclic aromatic hydrocarbons [57–60] and fullerene with graphite [61]. However, the importance of introducing the penetration effects in the molecular mechanics united-atom and all-atom force fields, commonly employed to describe the aliphatic systems [62–63], including such industrially relevant representatives as graphane [64] and polyethylene [65], is far less – if at all – recognized.

In the present work, we quantify the penetration energy in a diverse range of hydrocarbon dimers, including π-conjugated moieties and bulky aliphatic substituents. A direct one-to-one quantitative comparison between the fairly polarizable sp^2^-rich (π-conjugated) and the much less polarizable sp^3^-rich (aliphatic) systems demonstrates that charge penetration is important in both. While the energetic and structural consequences of neglecting this term are more drastic in the former, the resulting errors in the aliphatic dimer systems are nonetheless significant, i.e., ≈50% in interaction energy and 0.3 Å in interaction range. We discuss the implications of these results for the modeling of intermolecular interactions involving extended alkyl side chains, graphanes and various aliphatic systems in general.

## Results and Discussion

**Figure 2:**
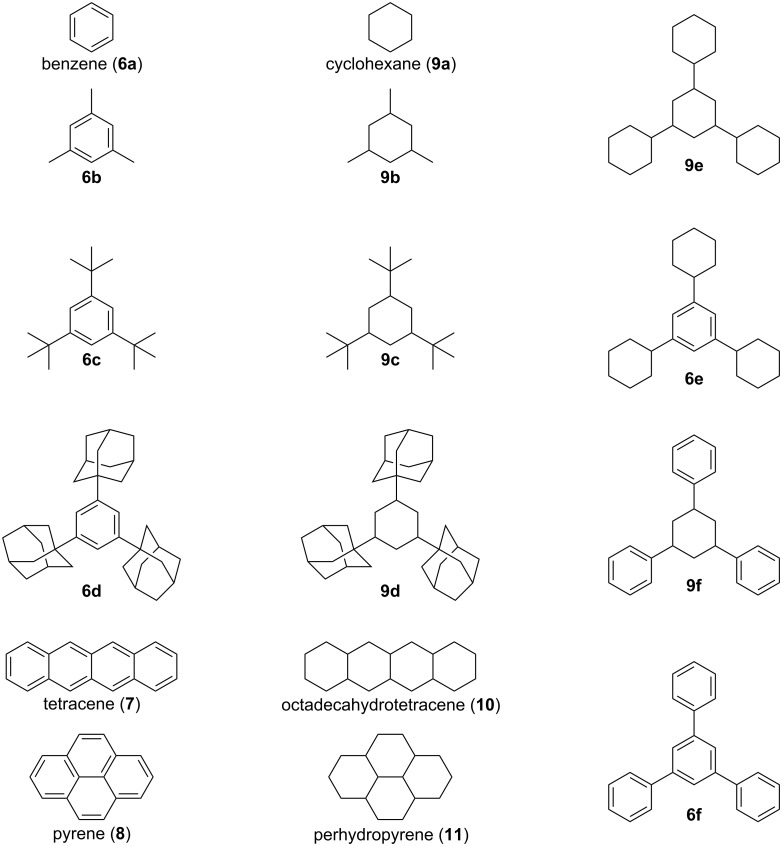
Studied monomer cores and their abbreviations, adopted here.

Here, we investigate the nature of non-covalent interactions for a range of hydrocarbon dimers featuring both aromatic and aliphatic skeletons and bearing substituents, from methyl all the way to bulky adamantyl (Figure 2). First, we consider the dimers, constructed from the optimized monomers that are kept fixed (frozen) in terms of all geometry parameters, except for the intermonomer distance, *d*. Configurations, corresponding to the lowest total interaction energy, *E*_tot_, in these constricted energy profiles (see Figures S1–S3 in Supporting Information File 1) are called ‘frozen dimers’ and are used in this work to compare the various systems on equal grounds. Second, to go beyond this somewhat constrained insight into the non-covalent interactions in the hydrocarbons, we relaxed the geometries of the frozen dimers. The resulting optimized dimers are, in general, structurally similar to the frozen counterparts albeit feature shorter interaction distances and in some cases undergo pronounced changes (e.g., lateral shifts and tilts) upon relaxation (see Figures S4 and S6 in Supporting Information File 1).

**Figure 3:**
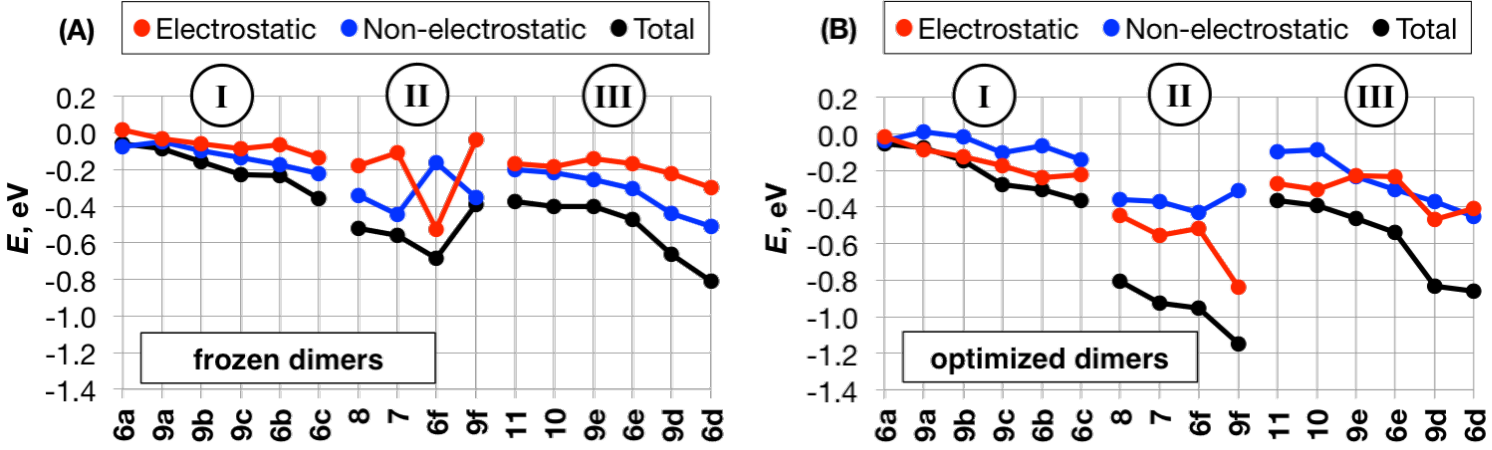
Breakdown of the SAPT0/jun-cc-pVDZ total interaction energies into electrostatic and non-electrostatic contributions in frozen (A) and optimized (B) dimers.

**Figure 4:**
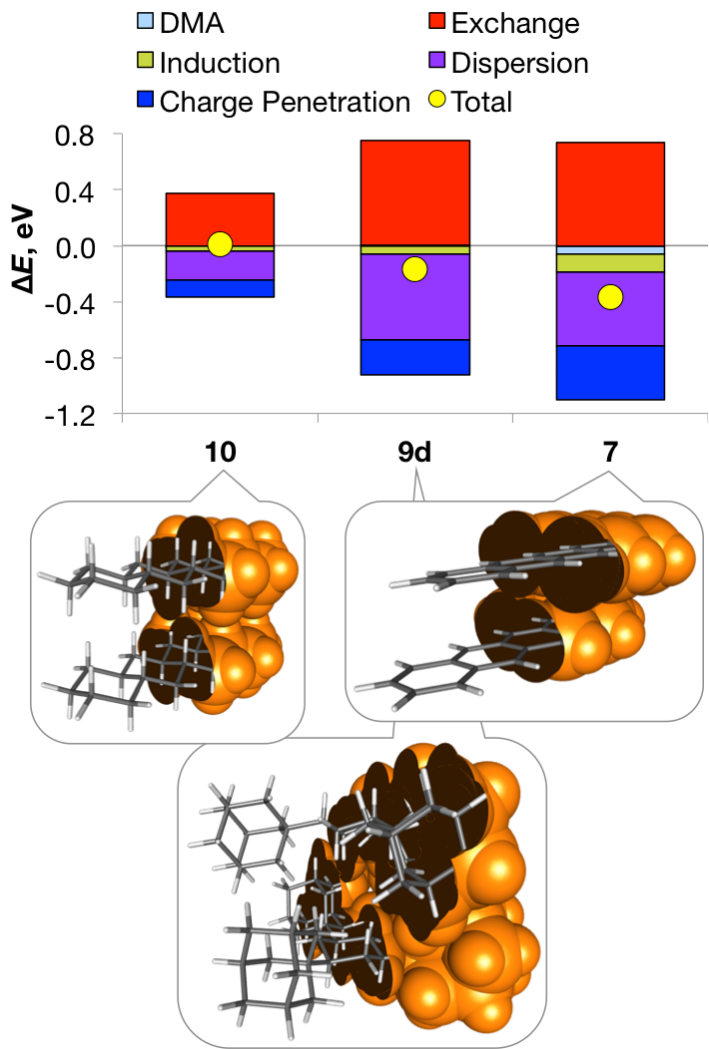
Decomposition of the SAPT0/jun-cc-pVDZ energy difference between the optimized and frozen dimers (*i.e.*, ∆*E* = *E*_total or component_[optimized] – *E*_total or component_[frozen]) for the representative cores from classes (II) and (III), as well as the M06-2X/def2-SVP geometries of their optimized dimers (clipped van der Waals surfaces are shown in orange).

We start by considering the electrostatic (*E*_elst_) and non-electrostatic (*E*_non-elst_) contributions to the total SAPT0/jun-cc-pVDZ intermolecular interaction energies (*E*_tot_) of the investigated dimers. The electrostatic part of *E*_tot_ consists of the distributed multipole and charge penetration terms (*E*_elst_ = *E*_DMA_ + *E*_Cpen_), while the non-electrostatic contribution includes exchange, dispersion and induction terms (*E*_non-elst_ = *E*_exch_ + *E*_disp_ + *E*_ind_). This breakdown allows us to discriminate between systems, driven by the non-electrostatic (*E*_elst_ < *E*_non-elst_), and those, driven by the electrostatic (*E*_elst_ > *E*_non-elst_) terms. Comparison between the two types of dimers – frozen and optimized – reveals the following three classes of hydrocarbons (Figure 3):

1. Non-substituted and substituted by comparatively non-bulky Me and *t-*Bu groups benzene and cyclohexane cores, **6a–c** and **9a–c**. These systems are associated with relatively small interaction energies, which do not change appreciably upon geometry relaxation. However, in their frozen dimers *E*_tot_ is dominated by the non-electrostatic term, while in their optimized assemblies electrostatics takes over. The intermonomer distances *d* are approx. 0.1–0.6 Å shorter in the optimized dimers, in which the monomers have the freedom to shift, e.g., laterally, compared to the frozen ones. The electrostatic term is thus almost entirely due to charge penetration (see Figure S7 in Supporting Information File 1), which increases exponentially at shorter range. The non-electrostatic term grows at a slower pace with shorter *d* since it depends both on exchange, which increases exponentially, and dispersion, which increases slower, i.e., as 1/*d*^6^ [66]. Similar behavior of *E*_Cpen_ and *E*_exch_ is rooted in their dependence on the extent of density overlap [67] and the following expression connecting them has been suggested as far back as 1970 [68]: *E*_exch_ = –*E*_Cpen_(*a* + *b* × *d*), where *a* and *b* are empirical parameters. The extent to which this linearity holds depends on the rank of multipolar extension, used to compute the *E*_Cpen_ from *E*_elst_, as well as the geometric features of the molecular core and its dimer [69].

2. Extended sp^2^-rich cores **7**, **8**, **6f** and **9f**, which have the freedom to shift and get significantly closer upon geometry relaxation (see Figure S8 in Supporting Information File 1). Notably shorter interaction range in their optimized dimers (by 0.3–1.3 Å) compared to the frozen ones is associated with stabilizing effects, similar to those in class (I) above and thus dominated by enhanced charge penetration, albeit significantly amplified by the extended system size (Figure 4).

3. Bulky, sterically congested sp^3^-rich cores **10**, **11**, **6d**,**e** and **9d**,**e**, which do not have the space to move considerably upon optimization. This results in a moderate decrease in the intermonomer distances and interaction energies upon geometry relaxation (by approx. 0.1–0.7 Å) due to the competition between destabilizing exchange and stabilizing dispersion, with the latter becoming increasingly dominant as the bulk of the substituents increases [3–4,28]. However, the associated shortening of the multiple CH···HC contacts between the bulky cyclohexyl and adamantyl units brings about appreciable – even dominant – electrostatic stabilization (Figure 4). The extent of charge penetration increases with the increasing number of close-range CH···HC contacts, e.g., from **9e** (1,3,5-tricyclohexylcyclohexane) to **10** (less bulky perhydrotetracene) to **9d** (1,3,5-triadamantylcyclohexane, see Figure S8 in Supporting Information File 1).

The crucial role of electrostatics is well appreciated in the interactions of polarizable π-conjugated cores [9,30]. Bulky sp^3^-rich systems, despite being significantly less polarizable and generally featuring smaller (in absolute terms) stabilization, also involve appreciable electrostatic contribution from charge penetration both in their intra- [28] and intermolecular (Figure 4) assemblies.

**Figure 5:**
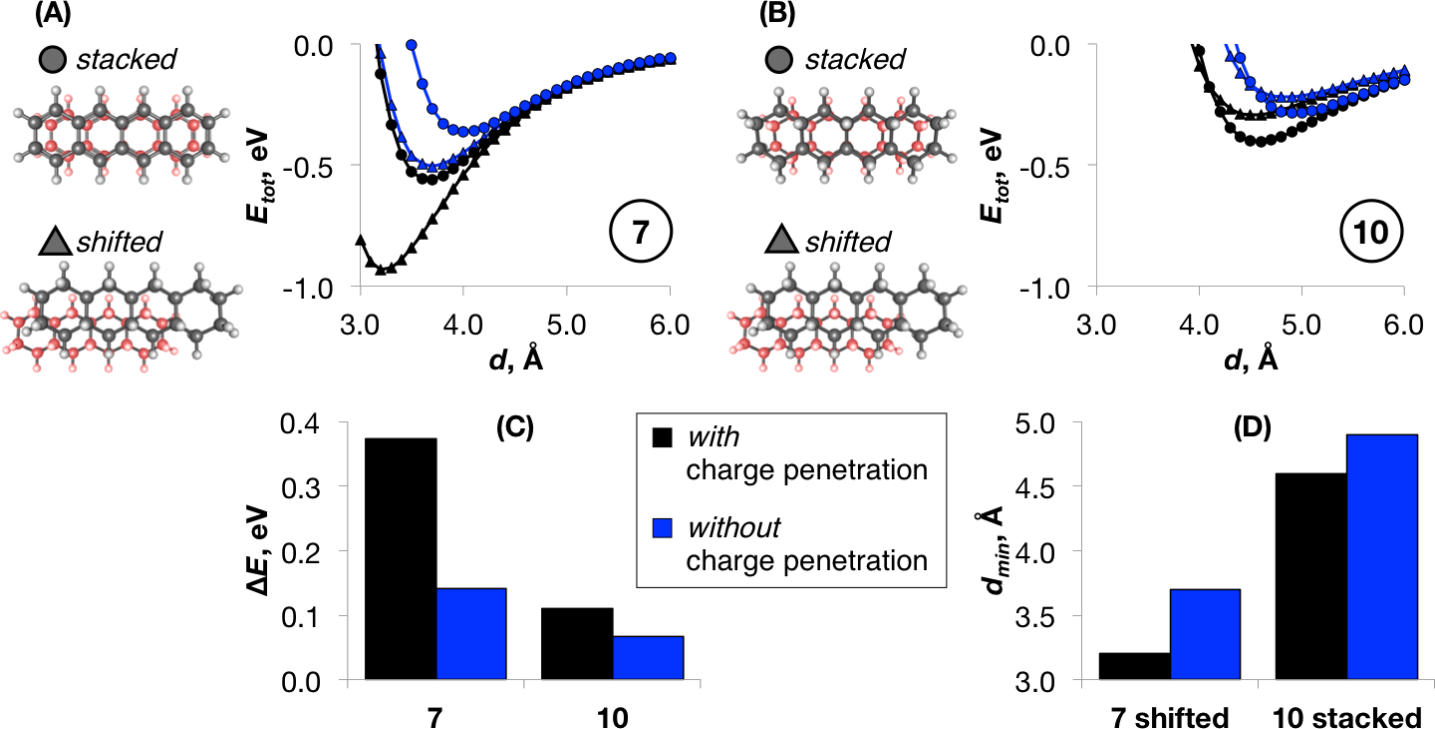
Structures and SAPT0/jun-cc-pVDZ interaction energy profiles with and without the charge penetration contribution vs varying intermonomer distance *d* for stacked and shifted dimers of tetracene (**7**, A) and perhydrotetracene (**10**, B). (C) The absolute SAPT0/jun-cc-pVDZ energetic difference between the energy minima on the shifted and stacked dimer profiles with and without the *E*_Cpen_. (D) The interplanar separation, corresponding to the minimum energy dimer for **7** and **10** with and without the *E*_Cpen_.

The demonstrated quantitative significance of *E*_Cpen_ nonetheless does not directly reflect its qualitative importance, nor does it reveal the implications of this term for the chemical and physical properties of the bulky π-conjugated and saturated hydrocarbons. To address this question, we have compared the SAPT0/jun-cc-pVDZ energy profiles of the dimer interplanar separation for representative class (II) and class (III) systems: tetracene (**7**) and its fully saturated analogue, perhydrotetracene (**10**, Figure 5A and B). For each monomer, four types of dimers were compared – perfectly stacked (i.e., the frozen dimer) and shifted transversally, laterally and in both directions by approx. half the ring (Figure S11 in Supporting Information File 1). The main purpose of this exercise is to mimic the results of geometrical relaxation with and without the charge penetration for a diverse sample of dimer arrangements, i.e., beyond the model frozen and optimized geometries. For tetracene, the shifted dimer is energetically favored, while for perhydrotetracene the stacked dimer is preferred. Our results in Figure 5C comparing selected – shifted and stacked – dimers (see all four dimers comparison in Figure S11 in Supporting Information File 1) illustrate that in tetracene (**7**) neglecting the penetration effects would result in a drastic underestimation of the energy difference between the stacked and shifted dimer geometries; in perhydrotetracene (**10**), even though this term accounts for almost half the difference in *E*_tot_, the relative error would be less significant. In terms of geometries of the energetically preferred dimers (Figure 5D), excluding *E*_Cpen_ leads to a longer interaction range both for **7** (by 0.5 Å) and, to a lesser extent, **10** (by 0.3 Å).

From the methodological viewpoint, these results have relevant implications on the use of existing and for the development of improved force fields (FFs) and other methods for the modeling and crystal structure prediction of hydrocarbons. In the case of π-conjugated complexes or assemblies, neglecting *E*_Cpen_ would flatten the potential energy surface dramatically. As shown in Figure 5, the penetration energy contribution strongly discriminates even between the slightly different (perfectly π-stacked and shifted by ≈1.2 Å in long and short axis) geometries. This contribution is therefore vital when exploring the free-energy landscape. While the energetic consequences are less pronounced for the sp^3^-rich systems, the absence of charge penetration would lead to elongated intermolecular distances. This might potentially be one of the reasons why the molecular mechanics force fields, commonly applied to aliphatics (see Introduction), significantly underestimate the liquid density and vapor pressure for long chain linear alkanes [70] and branched alkanes [71], fail to accurately reproduce the chain length dependence of the tilt and twist angles in alkanethiol self-assembled monolayers [72] and increasingly deviate (by as much as 15%) from experimental data for the hydrophobic solvation free energies of alkanes in alkanes with the increasing chain lengths [73].

## Conclusion

The significance of stabilizing dispersion and electrostatic effects within sterically hindered hydrocarbons is well recognized. In such systems, electrostatic contributions are generally dominated by charge penetration, which increases with system size (bulk) and shorter interaction distances. In the present work, we have performed a direct comparison between the sp^2^ and sp^3^-rich hydrocarbons and quantified the *E*_Cpen_ term of their intermolecular interactions. The electrostatic effects are, not surprisingly, important in systems with strong π–π interactions. Our results illustrate that the less polarizable saturated hydrocarbon dimers with increasingly more and shorter CH···HC contacts can also be significantly influenced by electrostatics. In absolute terms, the penetration energy is greater in the π-conjugated systems and is thus crucial for the correct modeling of the energetic and structural properties of their bulk assemblies. In the bulky aliphatic systems, this contribution still constitutes a significant portion of the total interaction energy and accounts for approx. 0.3 Å difference in the interaction range. This re-emphasizes the importance of accounting for these effects even when modeling saturated hydrocarbons and provides the context for the underperformance of the molecular mechanics force fields, commonly applied to aliphatics.

## Computational Details

Geometries of the isolated monomer cores were optimized at the M06-2X/def2-SVP level using Gaussian 09 software package [74]. The molar volume of each monomer was computed using the Monte-Carlo integration inside a contour of 0.001 electrons/Bohr^3^ density in conjunction with M06-2X/def2-SVP density. The dimers were constructed from optimized monomers by translating one monomer with respect to another along the perpendicular axis and, in some systems, rotating it around this axis by 60° to achieved a staggered arrangement (for details, see Figure S3 in Supporting Information File 1). A range of intermonomer distances, *d*, was screened (3.0–6.0 Å or 4.0–7.0 Å depending on the system) with a 0.1 Å step size (see Figures S1and S2 in Supporting Information File 1). For each of these ‘frozen’ dimer geometries, the total interaction energy was evaluated using the method, considered a bronze standard for non-covalent interactions [75] – the zeroth-order symmetry-adapted perturbation theory (SAPT0) with jun-cc-pVDZ basis [76], which allows decomposing the total interaction energy *E*_tot_ into the exchange *E*_exch_, electrostatic *E*_elst_, dispersion *E*_disp_ and induction *E*_ind_ components. SAPT0 computations were performed using the Psi4 code [77] and employed the density-fitting algorithm (DF-SAPT) [78–79]. For the distributed multipole analyses (DMA) [80] computations, the atom-centered multipoles up to the 8th-order were generated using Molpro [81] at the HF/6-311G** level (see also Figure S5A in Supporting Information File 1 regarding the different basis sets in SAPT0 and DMA computations). The multipole–multipole interaction energies were computed up to 32-poles (i.e., including all *R^–n^* terms, where *n* ≤ 6) using an in-house program of the Sherrill research group [31]. Charge penetration *E*_Cpen_ was evaluated as the difference between the electrostatic energy term of the SAPT0 total interaction energy, *E*_elst_, and the DMA electrostatic term *E*_DMA_. Furthermore, for each system the dimer with the lowest *E*_tot_ (called here the ‘frozen dimer’) was then used as a starting point for geometry relaxation at M06-2X/def2-SVP and PBE0-dDsC/def2-SVP levels, producing the ‘optimized dimer’. The two methods were used to allow comparison of different dispersion treatments and yielded very similar results (for details, see Figure S5B–D in Supporting Information File 1); for consistency, the M06-2X results are discussed in the manuscript. Energy decomposition analyses for the optimized dimers were performed in the same way as for the frozen dimers.

## Supporting Information

File 1 Additional figures, complete set of computed data and geometries of the studied monomers and dimers.
