# Supplementary material for: Steric “attraction”: not by dispersion alone
Source: Beilstein J Org Chem. 2018 Jun 19;14:1482–90. doi: 10.3762/bjoc.14.125 (PMC6037011; doi:10.3762/bjoc.14.125)
Supplement: File 1 — Additional figures, complete set of computed data and geometries of the studied monomers and dimers. [file Beilstein_J_Org_Chem-14-1482-s001.pdf]

Supporting Information

for

Steric “attraction”: not by dispersion alone

Ganna Gryn’ova and Clémence Corminboeuf*

Address: Institut des Sciences et Ingénierie Chimiques, École polytechnique fédérale de Lausanne, CH-1015 Lausanne, Switzerland

Email: Clémence Corminboeuf - clemence.corminboeuf@epfl.ch

* Corresponding author

Additional figures, complete set of computed data and geometries of the studied monomers and dimers

Table of Contents

Figure S1 ………………………………………………………………………… S2
Figure S2 ………………………………………………………………………… S3
Figure S3 ………………………………………………………………………… S4
Figure S4 ………………………………………………………………………… S5
Figure S5 ………………………………………………………………………… S6
Figure S6 ………………………………………………………………………… S7
Figure S7 ………………………………………………………………………… S7
Figure S8 ………………………………………………………………………… S7
Figure S9 ………………………………………………………………………… S8
Figure S10 ……………………………………………………………………….. S8
Table S1 …………………………………………………………………………. S9
Table S2 …………………………………………………………………………. S9
Geometries of the monomers ……………………………………………………. S11
Geometries of the frozen dimers ……………………………………………….. S19
Geometries of the optimized dimers …………………………………………….. S34

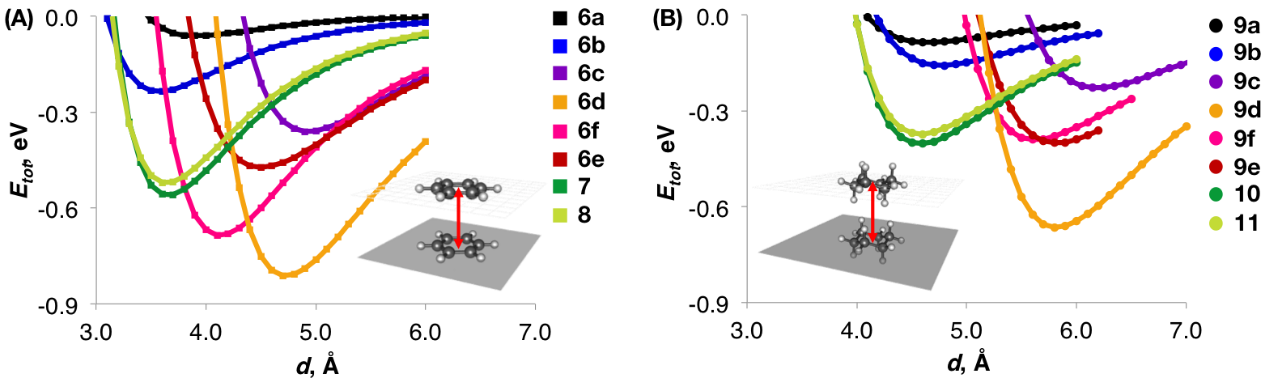

Figure S1: Computed SAPT0/jun-cc-pVDZ interaction energies E_{tot} for varied intermonomer distances d (schematically illustrated with red arrows for **6a** and **9a** dimers, see insets) for all investigated systems.

The interaction energy plots, shown in Figure S1, reveal a diverse interaction behavior: from rather shallow in smaller species, e.g., benzene (**6a**) and cyclohexane (**9a**), to pronounced deep minima in bulky **6d** and **9d**. Considering the various contributions to the total interaction energies, exchange is, of course, the dominant factor at short range but gives way to dispersion as d increases (Figure S2). In all species, charge penetration is appreciable at smaller d and decays with increasing separation. In contrast, the multipolar electrostatics, quantified by E_{DMA}, is negligible in all dimers, except **6a**, **6b**, **7** and **8**. In the latter systems, its contribution instead increases with larger d. The reason is that the multipolar electrostatics arising from quadrupole-quadrupole interactions (repelling π-clouds in the case of these π-conjugated monomers) decays slower (as $1/d^5$) than the exchange and charge penetration [1], and, at sufficiently large distances, than even dispersion (which decays as $1/d^6$). This effect vanishes moving from **6b** to **6c** due to much larger interaction distances, imposed by the steric bulk.

1 Ryno, S. M.; Risko, C.; Brédas, J.-L. *Chem. Mater.* **2016**, *28* (11), 3990-4000.

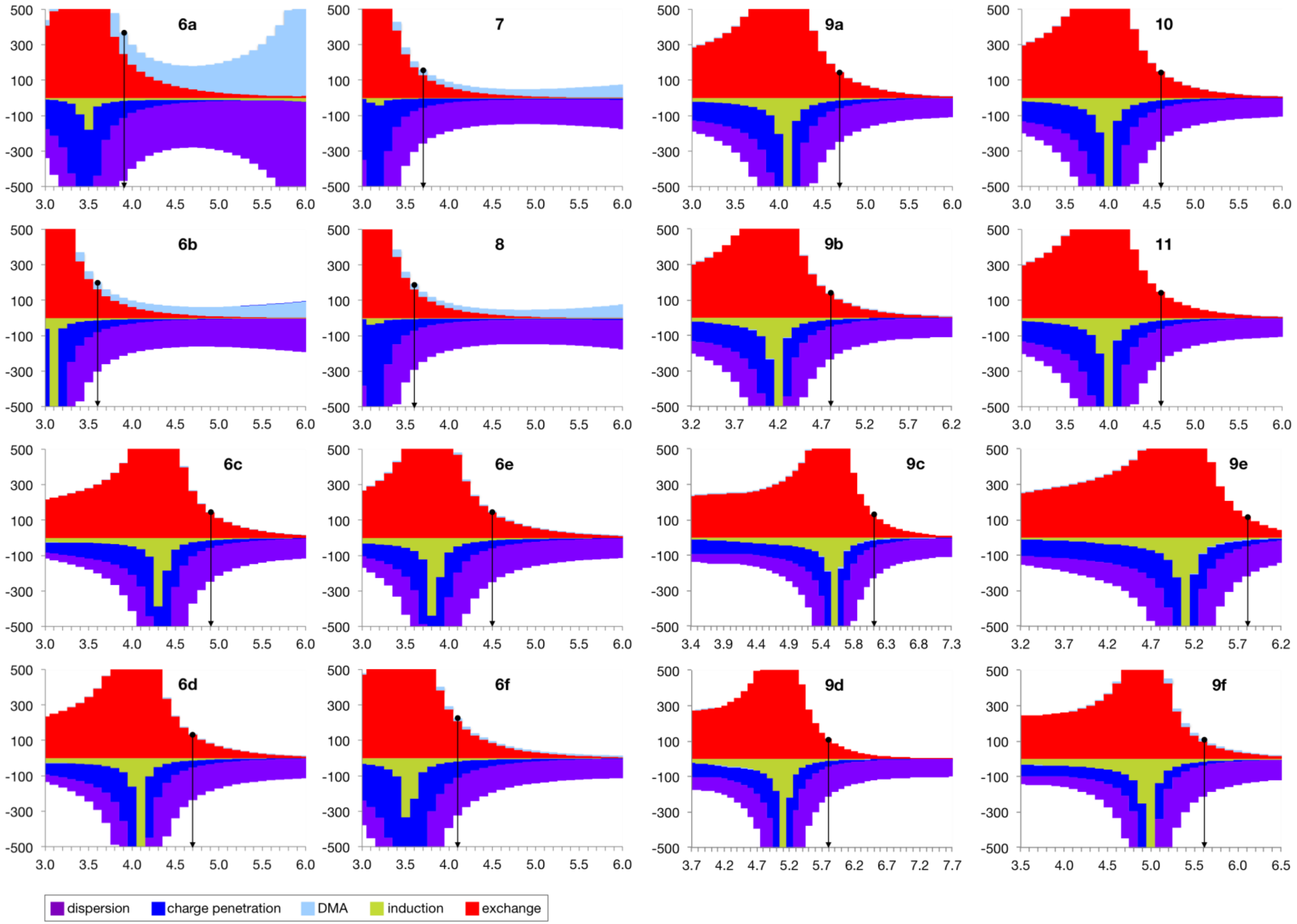

Figure S2: Percentile contributions to the total SAPT0/jun-cc-pVDZ interaction energies for the studied dimers with varied intermonomer separation (*x*-axis, in Å). Separations corresponding to the lowest E_{tot} are denoted with an arrow. *y*-Axis range is fixed to allow comparison and enhance the visibility around the lowest E_{tot}.

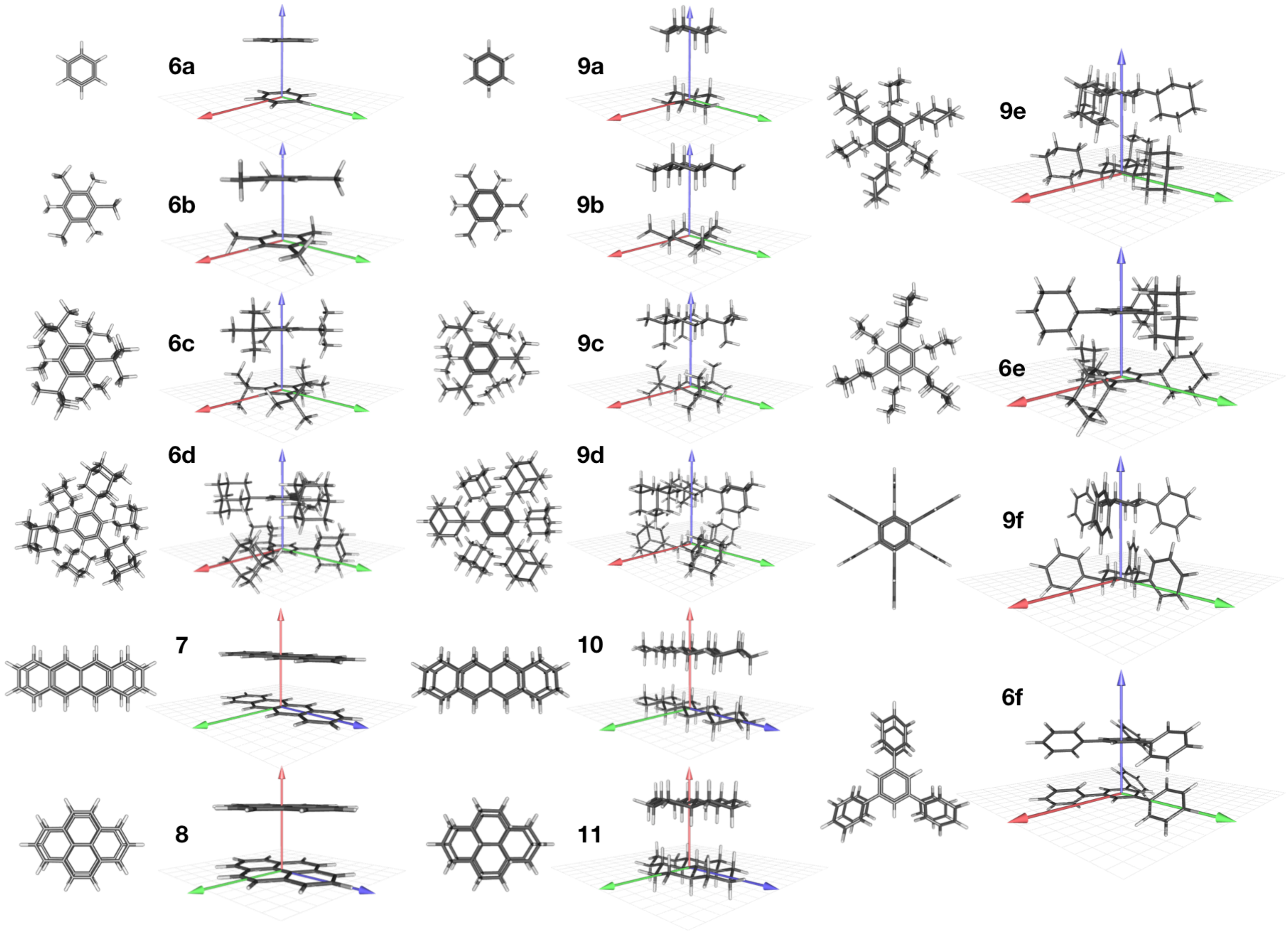

Figure S3: Frozen dimer geometries, assembled from M06-2X/def2-SVP optimized monomers. Systems are shown along the perpendicular axis (*i.e.*, top view, left) and from the side (right). Energy decomposition analysis was performed for changing intermonomer distance (0.1 Å step size along red axis for **7**, **8**, **10** and **11**, and blue axis for the rest).

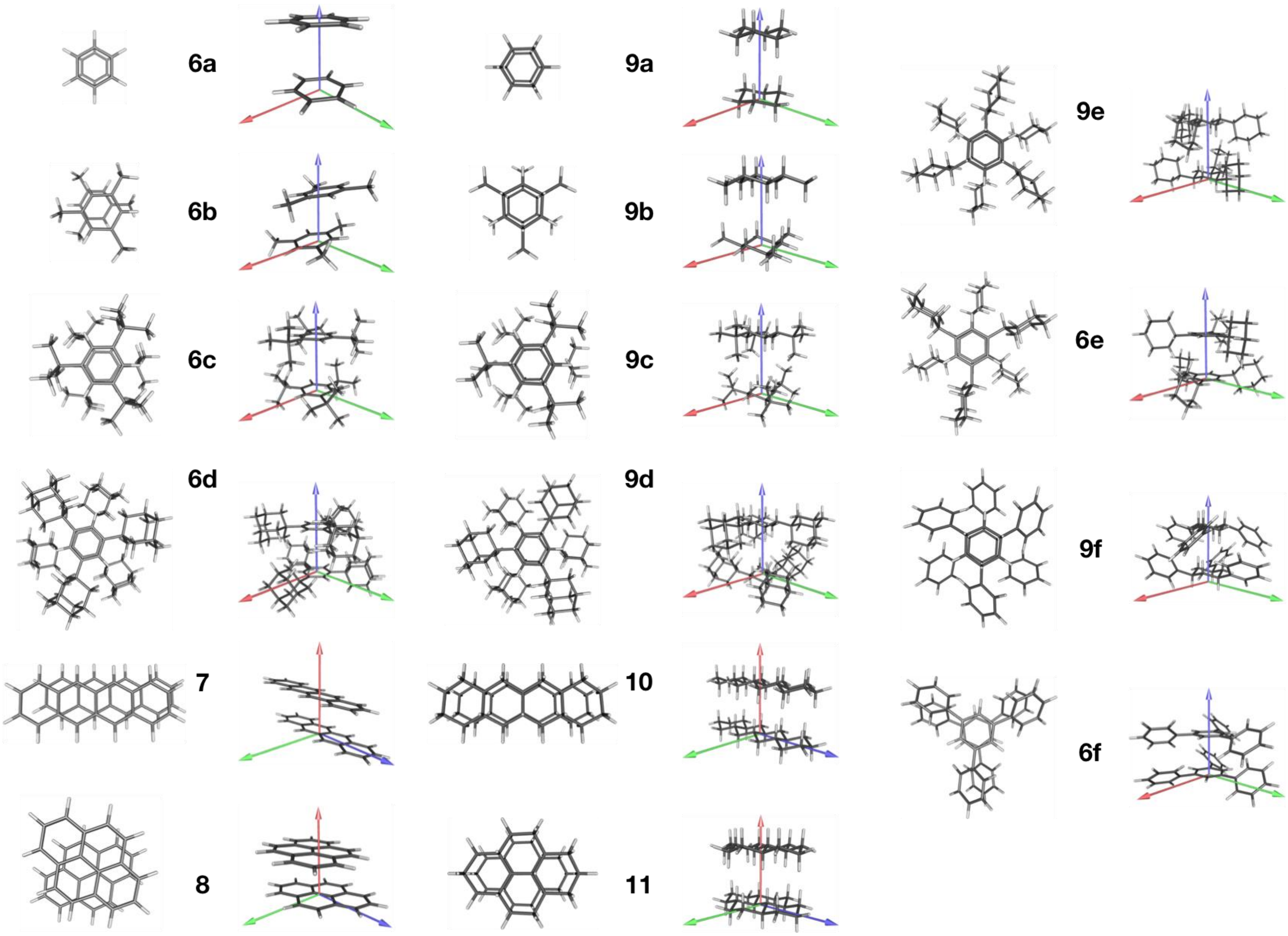

Figure S4: Optimized dimer geometries (M06-2X/def2-SVP level). Systems are shown along the perpendicular axis (*i.e.*, top view, left) and from the side (right).

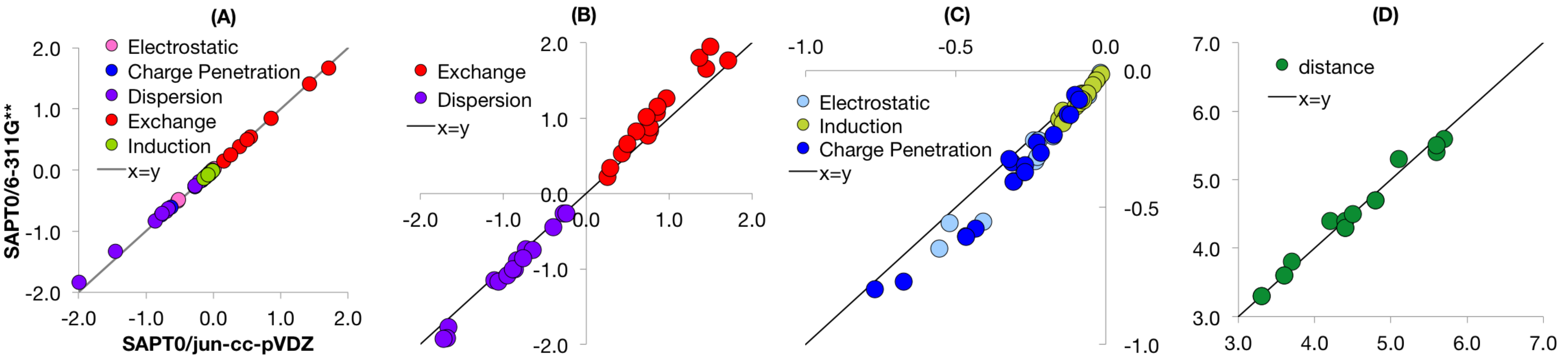

Figure S5: Comparison of the total interaction energies and their components for frozen and optimized dimers of **6a**, **6f**, **9f** and **11**, computed at SAPT0/jun-cc-pVDZ and SAPT0/6-311G** level in conjunction with HF/6-311G** DMA (A). Comparison of M06-2X/def2-SVP (*x*-axis) and PBE0-dDsC/def2-SVP (*y*-axis) optimized dimers: (B) exchange and dispersion energy components, in eV; (C) total electrostatic, charge penetration and induction energy components, in eV; (D) intermonomer distances, in Å.

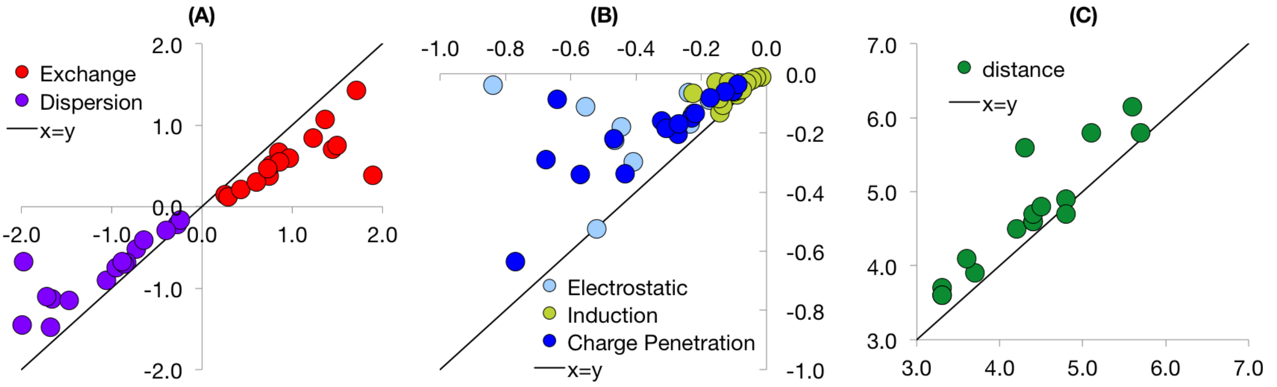

Figure S6: Comparison of the M06-2X/def2-SVP optimized (*x*-axis) and frozen (*y*-axis) dimers: (A) exchange and dispersion energy components, in eV; (B) total electrostatic, charge penetration and induction energy components, in eV; (C) intermonomer distances, in Å.

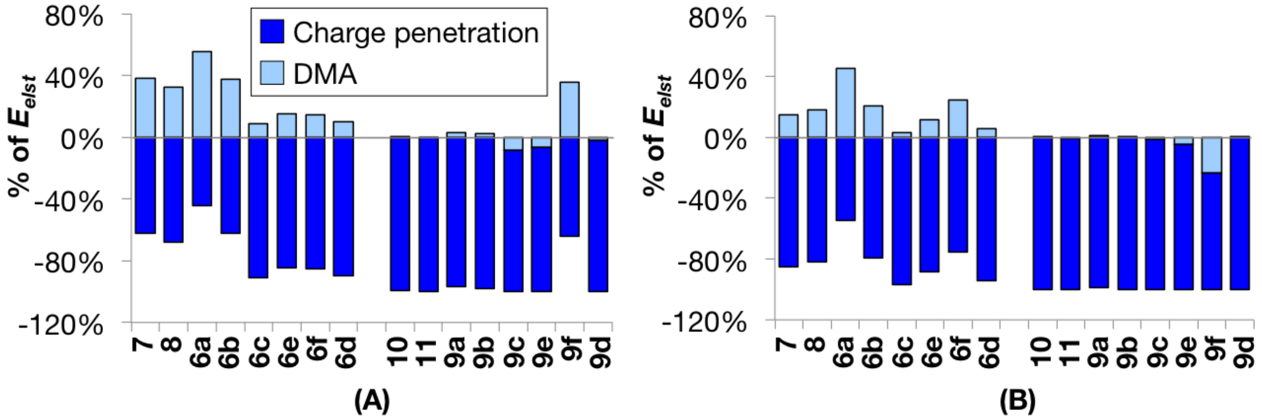

Figure S7: Percentages of the E_{Cpen} and E_{DMA} contributions to the total electrostatic energy in the frozen (A) and optimized dimers (B).

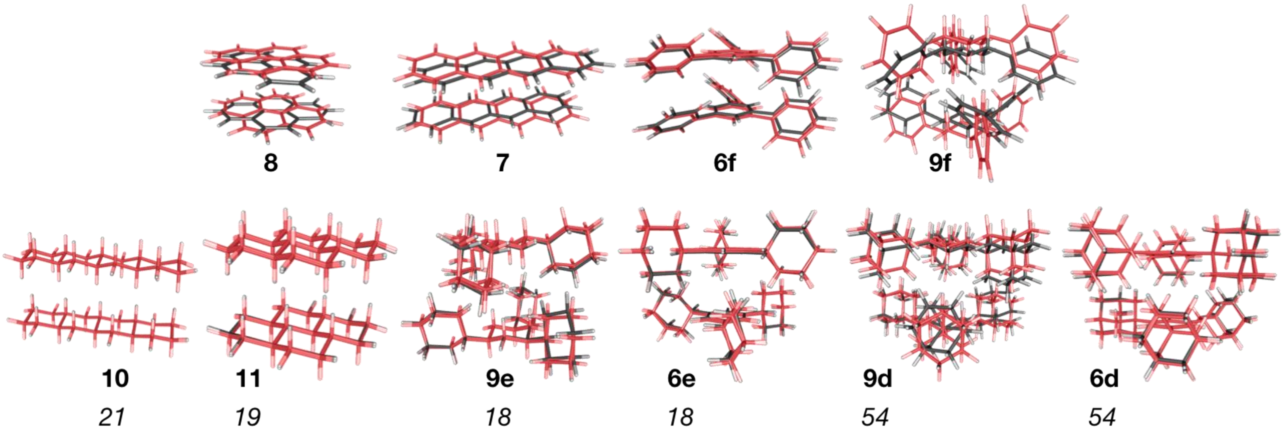

Figure S8: Structures of the frozen (in red) and optimized dimers in classes (II) and (III), top and bottom respectively. Numbers in italics for class (III) are quantitites of intermolecular CH···HC contacts < 3Å.

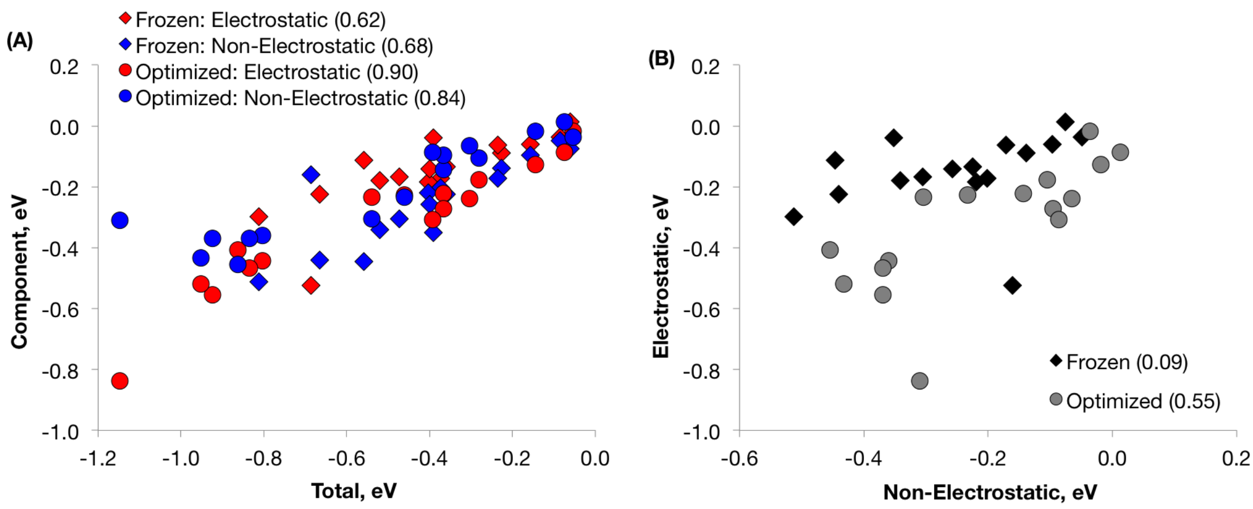

Figure S9: Probing the linear regression between the SAPT0/jun-cc-pVDZ total interaction energies and their electrostatic and non-electrostatic components in the frozen and optimized dimers. Numbers in brackets are R^2 values.

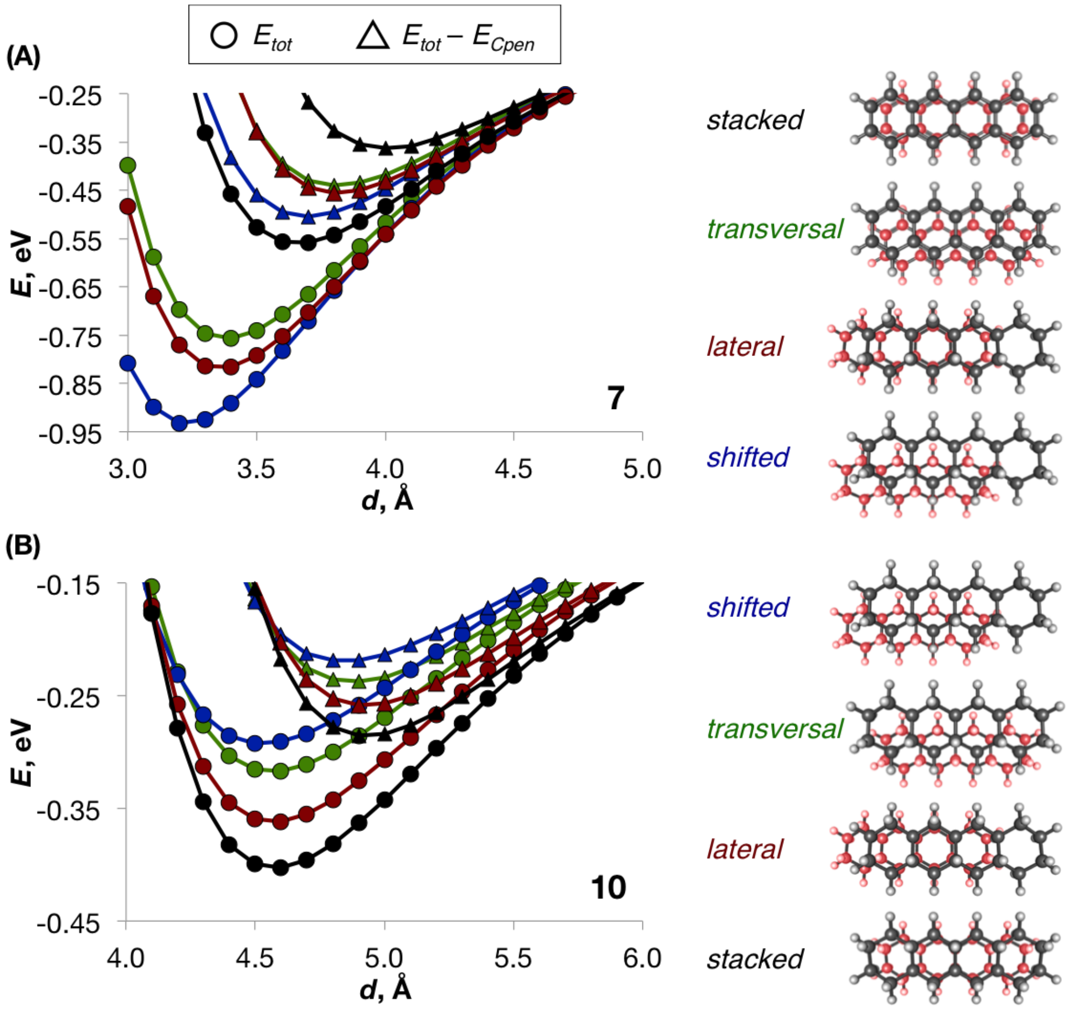

Figure S10: SAPT0/jun-cc-pVDZ interaction energy profiles with and without the charge penetration contribution for different dimers of tetracene (**7**, A) and perhydrotetracene (**10**, B): perfectly π-stacked (black), laterally (red) shifted by 1.24 Å in **7** and 2.54 Å in **10**, transversally (green) shifted by 1.12 Å in **7** and 2.17 Å in **10**, shifted (blue) both laterally and transversally by the aforementioned amounts.

Table S1: Computed properties of the frozen dimers.

Monomer	Volume, $Å^3mol^{-1}$	d, Å	E_{DMA}, eV	SAPT0/jun-cc-pVDZ energy decomposition, eV					E_{Cpen}, eV
				E_{elst}	E_{exch}	E_{ind}	E_{disp}	E_{tot}	
**7**	302	3.7	0.18	-0.11	0.71	-0.03	-1.13	-0.56	-0.29
**8**	259	3.6	0.16	-0.18	0.84	-0.03	-1.15	-0.52	-0.34
**6a**	120	3.9	0.07	0.01	0.15	-0.01	-0.22	-0.06	-0.06
**6b**	195	3.6	0.10	-0.06	0.38	-0.03	-0.52	-0.23	-0.16
**6c**	409	4.9	0.01	-0.14	0.51	-0.05	-0.69	-0.36	-0.15
**6e**	461	4.5	0.04	-0.17	0.67	-0.07	-0.91	-0.47	-0.20
**6f**	441	4.1	0.11	-0.52	1.43	-0.13	-1.46	-0.69	-0.63
**6d**	639	4.7	0.04	-0.30	1.07	-0.11	-1.48	-0.81	-0.34
**10**	356	4.6	0.00	-0.18	0.59	-0.06	-0.75	-0.40	-0.18
**11**	307	4.6	0.00	-0.17	0.55	-0.06	-0.70	-0.37	-0.17
**9a**	131	4.7	0.00	-0.04	0.12	-0.01	-0.16	-0.08	-0.04
**9b**	233	4.8	0.00	-0.06	0.21	-0.02	-0.29	-0.16	-0.06
**9c**	417	6.1	-0.01	-0.09	0.30	-0.03	-0.41	-0.23	-0.08
**9e**	486	5.8	-0.01	-0.14	0.47	-0.05	-0.67	-0.40	-0.13
**9f**	383	5.6	0.05	-0.04	0.39	-0.07	-0.68	-0.39	-0.09
**9d**	601	5.8	0.00	-0.22	0.75	-0.08	-1.11	-0.66	-0.22

Table S2: Computed properties of the optimized dimers.

Monomer	d, Å	E_{DMA}, eV	SAPT0/jun-cc-pVDZ energy decomposition, eV					E_{Cpen}, eV
			E_{elst}	E_{exch}	E_{ind}	E_{disp}	E_{tot}	
			M06-2X/def2-SVP					
**7**	3.3	0.12	-0.55	1.45	-0.16	-1.66	-0.92	-0.67
**8**	3.3	0.13	-0.44	1.23	-0.12	-1.47	-0.80	-0.57
**8 (stuck)**[2]	3.6	0.16	-0.16	0.77	-0.03	-1.11	-0.52	-0.31
**6a**	3.7	0.08	-0.02	0.26	-0.01	-0.28	-0.05	-0.10
**6b**	3.3	0.08	-0.24	0.74	-0.08	-0.73	-0.30	-0.32
**6c**	4.8	0.01	-0.22	0.77	-0.07	-0.84	-0.37	-0.23
**6e**	4.2	0.04	-0.23	0.85	-0.09	-1.06	-0.54	-0.27
**6f**	3.6	0.25	-0.52	1.71	-0.14	-2.00	-0.95	-0.77
**6d**	4.8	0.03	-0.41	1.36	-0.13	-1.68	-0.86	-0.43
**10**	4.4	0.00	-0.31	0.97	-0.10	-0.96	-0.39	-0.31
**11**	4.4	0.00	-0.27	0.86	-0.09	-0.86	-0.37	-0.27
**9a**	4.4	0.00	-0.09	0.29	-0.03	-0.24	-0.07	-0.09
**9b**	4.5	0.00	-0.13	0.43	-0.04	-0.40	-0.14	-0.13
**9c**	5.6	0.00	-0.18	0.61	-0.06	-0.65	-0.28	-0.17
**9e**	5.7	-0.01	-0.23	0.73	-0.07	-0.89	-0.46	-0.22
**9f**	4.3	-0.20	-0.84	1.89	-0.23	-1.97	-1.15	-0.64
**9f (stuck)**[2]	5.6	0.06	-0.06	0.50	-0.08	-0.76	-0.41	-0.12
**9d**	5.1	0.00	-0.47	1.50	-0.14	-1.72	-0.84	-0.47

PBE0-dDsC/def2-SVP

[2] The 'stuck' local minimum is very similar to the frozen geometry; this is the structure captured at the PBE0 level and used for comparisons in Figure S5.

**7**	3.3	0.12	-0.65	1.65	-0.18	-1.77	-0.94	-0.77
**8**	3.6	0.16	-0.18	0.83	-0.03	-1.15	-0.53	-0.34
**6a**	3.8	0.08	-0.01	0.22	-0.01	-0.26	-0.06	-0.09
**6b**	3.3	0.07	-0.25	0.77	-0.08	-0.73	-0.31	-0.32
**6c**	4.7	0.01	-0.25	0.88	-0.09	-0.89	-0.35	-0.26
**6e**	4.4	0.02	-0.33	1.07	-0.11	-1.17	-0.54	-0.35
**6f**	3.6	0.24	-0.56	1.77	-0.15	-2.01	-0.95	-0.80
**6d**	4.7	0.03	-0.55	1.81	-0.17	-1.92	-0.84	-0.58
**10**	4.3	0.00	-0.40	1.26	-0.13	-1.09	-0.36	-0.41
**11**	4.4	0.00	-0.37	1.16	-0.12	-1.00	-0.33	-0.37
**9a**	4.3	0.00	-0.10	0.34	-0.04	-0.27	-0.07	-0.11
**9b**	4.5	0.00	-0.16	0.53	-0.05	-0.45	-0.13	-0.16
**9c**	5.4	0.00	-0.24	0.83	-0.08	-0.75	-0.24	-0.24
**9e**	5.6	-0.01	-0.32	1.01	-0.11	-1.01	-0.41	-0.30
**9f**	5.5	0.07	-0.09	0.66	-0.11	-0.86	-0.39	-0.16
**9d**	5.3	0.00	-0.60	1.95	-0.19	-1.93	-0.78	-0.61

Geometries of the studied species

(on a page, left bottom of left column continues on top of the right column)

Monomers (M06-2X/def2-SVP)

7
30
0 1
C 0.0000000013 0.7229984214 -2.4446946599
C -0.0000000013 -0.7229984214 -2.4446946599
C 0. -1.4094335199 -3.7084720514
C -0.0000000044 -0.7174238937 -4.8813880483
C 0.0000000045 0.7174238937 -4.8813880483
C -0.0000000007 1.4094335199 -3.7084720514
C 0.0000000002 1.4051504752 -1.2346796945
C 0.0000000002 -1.4051504752 -1.2346796945
H -0.0000000004 -2.5012416025 -3.7053575762
H -0.0000000041 -1.2518046335 -5.8324011925
H 0.0000000045 1.2518046335 -5.8324011925
H -0.0000000002 2.5012416025 -3.7053575762
C -0.000000001 -0.7223507702 0.
C 0.0000000013 0.7223507702 0.
H 0.0000000007 2.497865981 -1.2353750297
H -0.0000000005 -2.497865981 -1.2353750297
C 0.0000000017 -1.4051504752 1.2346796945
C -0.0000000013 1.4051504752 1.2346796945
C 0.0000000004 0.7229984214 2.4446946599
C 0.0000000001 -0.7229984214 2.4446946599
H 0.0000000008 -2.497865981 1.2353750297
H -0.0000000009 2.497865981 1.2353750297
C -0.0000000014 1.4094335199 3.7084720514
C 0.0000000012 -1.4094335199 3.7084720514
C 0.0000000009 0.7174238937 4.8813880483
C -0.0000000008 -0.7174238937 4.8813880483
H -0.000000003 2.5012416025 3.7053575762
H 0.0000000026 -2.5012416025 3.7053575762
H 0.0000000007 1.2518046335 5.8324011925
H -0.0000000009 -1.2518046335 5.8324011925

8
26
0 1
C -0.0000000013 -2.4646083636 0.6784110538
C -0.0000000015 -2.4646083636 -0.6784110538
C 0.0000000002 -1.2322347528 -1.427657105
C -0.0000000014 0. -0.7148181042
C -0.0000000008 0. 0.7148181042
C -0.0000000002 -1.2322347528 1.427657105
C -0.0000000011 -1.2093090131 -2.8289670289
C 0.0000000037 0. -3.5198747389
C -0.0000000015 1.2093090131 -2.8289670289
C 0.0000000002 1.2322347528 -1.427657105
H -0.0000000008 -3.4057630201 1.2320478493
H -0.0000000008 -3.4057630201 -1.2320478493
C -0.0000000001 1.2322347528 1.427657105
C -0.0000000011 -1.2093090131 2.8289670289
H 0.0000000018 -2.1549726742 -3.3746816086
H 0.0000000012 0. -4.6108342634
H 0.0000000015 2.1549726742 -3.3746816086
C -0.000000002 2.4646083636 -0.6784110538
C -0.0000000007 1.2093090131 2.8289670289
H 0.0000000012 -2.1549726742 3.3746816086
C 0.000000003 0. 3.5198747389
H -0.0000000009 3.4057630201 -1.2320478493
C -0.0000000008 2.4646083636 0.6784110538
H 0.0000000016 2.1549726742 3.3746816086
H 0.0000000014 0. 4.6108342634
H -0.0000000008 3.4057630201 1.2320478493

6a
12
0 1
C 1.2079608624 -0.6974210355 0.
C 1.2079608624 0.6974210355 0.
C 0. 1.3948256098 0.
C -1.2079608624 0.6974210355 0.
C -1.2079608624 -0.6974210355 0.
C 0. -1.3948256098 0.
H 2.1528905787 -1.2429361064 0.
H 2.1528905787 1.2429361064 0.
H 0. 2.485924516 0.
H -2.1528905787 1.2429361064 0.
H -2.1528905787 -1.2429361064 0.
H 0. -2.485924516 0.

6b
21
0 1
C 1.3284365791 0.4002214307 0.00463027
C 1.0216714304 -0.968558135 0.0045546892
C -0.3175988377 -1.3505867108 0.0039826371
C -1.3495907644 -0.4005529723 0.0032922306
C -1.0107787011 0.9503655573 0.0032904582
C 0.3279444779 1.3690473118 0.0040112288
H -0.5713808095 -2.4145214769 0.0040906401
C -2.78848008 -0.8482624892 0.0024943698
H -1.805346723 1.7020421242 0.0028255962
C 0.6596145485 2.8390569573 0.0045969476

H 2.3767093779 0.7124072443 0.0052419072
C 2.1289107975 -1.9907707533 0.0041986548
H -3.4740532778 0.0090006801 0.003491647
H -3.0097578249 -1.4632221428 0.8873841122
H -3.0094826856 -1.4608699097 -0.8841053723
H 1.744789141 3.0042484558 0.0014287799
H 0.2413475758 3.3363547909 0.8922704475
H 0.235729594 3.3384044634 -0.8792181025
H 1.7293764347 -3.0131164466 0.0133914174
H 2.7768248581 -1.8694793147 0.8848810309
H 2.7650848888 -1.8812086644 -0.8866035896

6c
48
0 1
C -0.7222544484 -1.1820381134 0.0132837638
C -1.4116567927 0.03664539 -0.1212798151
C -0.6697458205 1.2135229544 -0.1166116014
C 0.7304104407 1.2086975346 0.018078652
C 1.3720741985 -0.0188105463 0.148786582
C 0.6614499071 -1.232659174 0.1486887653
H -1.1801283356 2.1710421777 -0.2196538863
C 1.4858517958 2.5428679197 0.0151693172
H 2.4563820971 -0.049988496 0.2545358146
C 1.4324120872 -2.5496669764 0.2959909374
H -1.2964972229 -2.1083899818 0.0102404876
C -2.9378588123 0.0191147261 -0.266291796
C 0.9860486649 3.4121533008 1.1806873676
C 1.2263674109 3.2702027068 -1.3143819615
C 2.9967881971 2.3503004596 0.1716729195
C 0.5033732875 -3.7665672563 0.2762232243
C 2.1950003346 -2.5451748861 1.6310746222
C 2.4319600835 -2.6854761309 -0.8643863163
C -3.5581509012 -0.6423543684 0.9753422126
C -3.3194908283 -0.7853329894 -1.5197412328
C -3.5202924117 1.4285793237 -0.4028239246
H -0.0897362799 3.6221383485 1.096876813
H 1.5187285077 4.3756144494 1.1930868084
H 1.1589077042 2.9079490154 2.1429386892
H 0.1565589784 3.4764004378 -1.4609425589
H 1.5737666663 2.6627870629 -2.1631428748
H 1.7619900275 4.2319184254 -1.3335084199
H 3.2445368716 1.853171877 1.1211016829
H 3.4963027667 3.3301469842 0.1646201646
H 3.415483575 1.7532905928 -0.65188378
H -0.2211731701 -3.7351711962 1.103264956
H 1.0972309965 -4.6860331898 0.3839274667
H -0.053384395 -3.8345587276 -0.6700046785
H 1.4977574369 -2.4450407912 2.4759767743
H 2.9130788221 -1.7145004476 1.6842629593
H 2.7553673927 -3.4850294667 1.7528700915
H 1.9067845614 -2.6870633514 -1.830931612
H 2.9952123811 -3.6271362611 -0.7738318581
H 3.1560868938 -1.858412895 -0.873829834
H -3.2936427015 -0.0840409634 1.8854882883
H -3.2091780718 -1.6773979911 1.0996071999
H -4.655435078 -0.6634989046 0.8876451177
H -2.963808299 -1.8237077279 -1.4580956028
H -2.8823639232 -0.3303474199 -2.4208197736
H -4.4137683031 -0.8090255935 -1.6387377459
H -3.2983349456 2.0449300436 0.4807921916
H -4.6137432632 1.3677601361 -0.5045345163
H -3.127788083 1.9434259797 -1.2921010807

6e
60
0 1
C 1.350586464 0.3084466026 0.4036606813
C 0.4020782203 1.3301560156 0.4387063336
C -0.9725419717 1.054420167 0.4574249449
C -1.3832888432 -0.2781147255 0.4405304207
C -0.4576704489 -1.3306574761 0.4055975877
C 0.9016453935 -1.0196872112 0.3875283744
C 2.830856069 0.6248301923 0.3880149054
C -1.9867904564 2.1773480081 0.4974381595
C -0.9237759955 -2.7707467053 0.389304572
C -1.7032280698 -3.1428939517 1.6603953029
C -2.141019069 -4.607352052 1.6434468369
C -2.9630724299 -4.9259431222 0.3946164827
C -2.1948164454 -4.5626207305 -0.8760615448
C -1.7568678567 -3.0981797256 -0.859754518
C -1.8726762862 3.0172079912 1.779392812
C -2.9233766898 4.1266785073 1.8201815888
C -2.8366079971 5.0189352637 0.5819287016
C -2.9521805637 4.1930052217 -0.6992424586
C -1.9011940501 3.0838055109 -0.7404664386
C 3.5235720008 0.1117455589 -0.8841225146
C 5.0110707471 0.4638558673 -0.8958722878
C 5.7162407803 -0.065906907 0.3527004112
C 5.0369648099 0.4422640356 1.6243991704
C 3.549521881 0.0905794244 1.6369452152
H 0.7466555862 2.3683421229 0.4521688449
H 1.6283873658 -1.8369190782 0.3606363273
H -2.4545991818 -0.4991060837 0.4554065827
H -2.0094328323 2.4752014637 -1.6512487764
H -0.8951473426 3.5370326915 -0.7820661612
H -1.96136217 2.3612331563 2.6588756422
H -0.8650158029 3.4668867292 1.8221081788
H 3.0610048521 0.4862867494 2.5405005827
H 3.4344775012 -1.0071112646 1.6718186547

H 3.4087944558 -0.9852630674 -0.9355124114
H 3.0168087733 0.5223569852 -1.7708310626
H -2.6510373375 -2.4504395393 -0.8718538302
H -1.1786070293 -2.8520330868 -1.7634503848
H -2.5954982879 -2.4964613446 1.7334429576
H -1.0866414833 -2.9284555757 2.546671057
H -2.8569130302 4.8395112587 -1.5847089519
H -2.807760822 4.7258622867 2.7360007252
H -3.616367327 5.7949260806 0.6113960835
H -1.8659778253 5.5452346847 0.5847222885
H 5.5320953183 0.030436821 2.5167898527
H 5.4877893402 0.0676813696 -1.8052084431
H 6.7792956864 0.2184915556 0.3441578376
H 5.6838466567 -1.1695399079 0.3435545086
H -2.8066150089 -4.7661613338 -1.7678968136
H -2.7142665004 -4.8430169577 2.5528194198
H -3.2472935814 -5.9890061123 0.3817744755
H -3.9030054375 -4.347379565 0.4250162663
H 2.933614609 1.7247500421 0.3961161909
H -0.0226875383 -3.4091612138 0.3589735755
H 5.1489375772 1.5392210008 1.6797485849
H 5.1218478772 1.5616491119 -0.9346385693
H -1.3007970151 -5.2057657668 -0.9535791231
H -1.2449188062 -5.2520597634 1.6598403342
H -2.9904550853 1.7156970271 0.4966660363
H -3.9576378561 3.7394281883 -0.7467959628
H -3.9277314906 3.6705625763 1.8666417441

6f

42
0 1
C 1.2232921081 -0.6946191906 -0.0766149293
C 1.2015786849 0.7026105953 -0.0006788592
C -0.0056172466 1.4058157574 0.0813670943
C -1.205743015 0.6860840499 0.086363488
C -1.2137533968 -0.7113392297 0.0109741617
C 0.0081489498 -1.388814837 -0.0697861991
H 2.1437315818 1.2542258901 -0.0057976889
H -2.1532648523 1.2247826658 0.1498298461
H 0.0134382874 -2.478995056 -0.1288830598
C -0.0130909892 2.8903226556 0.1605714593
C 0.8961543113 3.6524111671 -0.5863361572
H 1.6003483169 3.1476819008 -1.2504530802
C 0.8887779139 5.0434201097 -0.5132961925
H 1.5984605525 5.6197710055 -1.1088506144
C -0.0277040175 5.6980550578 0.3088205947
H -0.033435492 6.787444356 0.3663136874
C -0.9367953194 4.9509647877 1.0570348144
H -1.6520976385 5.4539580667 1.7096243433
C -0.9297197181 3.5599777342 0.9831954462
H -1.6284991214 2.9807847621 1.5897702123
C -2.4968555076 -1.4620062582 0.0148268689
C -2.6157732537 -2.6765537086 0.7049584673
H -1.7614793126 -3.0606203614 1.2656496948
C -3.8182867622 -3.3795758144 0.7076213145
H -3.8940775911 -4.3188443972 1.2576862741
C -4.9240482067 -2.8813605045 0.0195452562
H -5.8657478162 -3.4320842314 0.0213614688
C -4.8181097157 -1.6744166789 -0.6707943189
H -5.6753605205 -1.2807692519 -1.2191328496
C -3.6157188174 -0.971304188 -0.672715809
H -3.5330974033 -0.0394697917 -1.2353321396
C 2.5137348547 -1.42761549 -0.1640573107
C 2.6416269105 -2.5589527383 -0.9820363588
H 1.7886918754 -2.8888994424 -1.5781168064
C 3.8510720728 -3.245009754 -1.0652124421
H 3.9336159139 -4.1182917613 -1.7141275853
C 4.9550216134 -2.812671139 -0.3312049021
H 5.902247398 -3.3499287688 -0.3962971559
C 4.8401330108 -1.68900854 0.4864400622
H 5.6960100234 -1.3480749448 1.0710505909
C 3.630743182 -1.0027792767 0.5689328645
H 3.5415131522 -0.1382062067 1.2294104487

6d

84
0 1
C -0.9656044306 1.0304893778 0.0086735106
C 0.4097636173 1.3249426496 0.0090968285
C 1.3760761905 0.3232204886 0.0028867673
C 0.9434070043 -1.015047916 -0.0078540285
C -0.4071847035 -1.3512205123 -0.0069638033
C -1.3495363772 -0.3072548191 0.0045513442
C -1.973962587 2.1772902997 0.0057646982
H 0.7185298671 2.3698967776 0.0134162961
C 2.8738451607 0.621223203 0.0077679906
H 1.6939855073 -1.804909475 -0.0168970857
C -0.8981736434 -2.7971979079 -0.0150703948
H -2.4087524465 -0.5623520814 0.0083157851
C -1.7757728379 3.0655840306 1.2570223524
C -3.4325693225 1.6815024763 0.0051867729
C -1.7717546318 3.055085437 -1.2525049755
C 3.5412961719 0.0085049613 -1.2462328191
C 3.5320192458 0.0010260449 1.2631496622
C 3.1769798986 2.1315144685 0.0135020523
C 0.2568419267 -3.8146444087 -0.0749997157
C -1.8095414448 -3.0423298602 -1.2412894241
C -1.7165139932 -3.0841879778 1.2661893969
H -1.9209379801 2.4506648546 2.1607731476
C -2.7541022079 4.2478924877 1.2496651034

H -0.740476435 3.4411501832 1.2873972878
H -3.610552947 1.0552598521 0.8951834704
H -3.6073683754 1.048520778 -0.8806199821
C -4.4172086329 2.8585421265 -0.0015294657
H -0.73628307 3.4301893936 -1.2824127596
C -2.7500207631 4.2371992765 -1.2590072419
H -1.9135694688 2.4321142106 -2.151266532
H 3.3461295135 -1.0751784639 -1.2808238595
C 5.0550432952 0.2603096076 -1.2393872513
H 3.0816733548 0.4465098653 -2.1478375928
H 3.3360911886 -1.0828294045 1.2893478217
H 3.0653757395 0.4335254866 2.1638077457
C 5.0457306578 0.2530772513 1.2693620231
H 2.7239827695 2.602951877 -0.8744778784
C 4.6896877794 2.3918184169 0.0197374088
H 2.7183015094 2.597335365 0.9014859049
H 0.8603881981 -3.6357945469 -0.9804674376
C -0.2745546732 -5.2546119851 -0.0822055875
H 0.920394757 -3.6725169566 0.7940416718
H -1.234772097 -2.8440815714 -2.1612724811
H -2.6513023353 -2.3320159787 -1.2296041692
C -2.3492313369 -4.4787187846 -1.2451064543
H -1.0750510815 -2.915125863 2.1470391379
C -2.2545323436 -4.521567475 1.2616732808
H -2.5549033965 -2.3728064473 1.3385364812
C -4.194439351 3.7201739967 1.2481465177
C -4.190243697 3.7092499813 -1.2579236188
C -2.519193328 5.0965728054 -0.0079363327
H -2.5850577485 4.8624884878 2.1477274129
H -5.4458063446 2.4654133071 -0.001587144
H -2.5778450951 4.8439623749 -2.1618003553
H -4.906898478 4.5613010371 1.2599026316
H -4.3784253611 3.1232255853 2.1563820226
H -4.90287615 4.5500296994 -1.2796323262
H -4.370842394 3.1043037628 -2.1615436253
H -3.2024649909 5.9616174225 -0.0126814414
H -1.4908155327 5.4940630392 -0.0080202897
C 5.67188191 -0.3736686189 0.0154961956
C 5.3124050292 1.7636960749 1.2733187114
C 5.3218918697 1.7708674861 -1.2326361853
H 5.5007747502 -0.1913569677 -2.1394695837
H 5.48467521 -0.2037133694 2.1702016558
H 4.8663812324 3.4787010029 0.0235453396
H 6.763101384 -0.2174474375 0.0199842638
H 5.4988292234 -1.4625277962 0.0117751307
H 6.3973752295 1.9576545659 1.2947180772
H 4.880831931 2.2205045577 2.178963203
H 6.4070115521 1.9648251532 -1.2445642873
H 4.8972973144 2.2329308786 -2.1389023047
C -1.0809642002 -5.5075887129 1.1981379517
C -1.1760519216 -5.4644701657 -1.305893046
C -3.157955921 -4.7232861689 0.0367527882
H 0.5774615648 -5.9507949478 -0.1264258107
H -2.9977065298 -4.6187149287 -2.1241529334
H -2.8345673962 -4.6934867105 2.1819013689
H -1.4555010601 -6.5442979864 1.2110032554
H -0.434889275 -5.3825389693 2.0824901655
H -1.551040806 -6.5009160346 -1.3261380971
H -0.5989698784 -5.3074868353 -2.2318545168
H -3.5685498685 -5.7463174645 0.0346372536
H -4.0134516634 -4.0292159161 0.0808949508

10
48
0 1
C 0.1474520022 -1.4563147864 1.2698802159
C -0.2722225757 -0.7177896326 0.0000000012
C 0.2722225748 0.7177896337 0.0000000012
C -0.1474520027 1.4563147871 1.2698802164
C 0.2712288378 0.7188193386 2.54073987
C -0.2712288379 -0.7188193385 2.5407398699
C 0.1503923286 -1.4621827497 3.8091953574
C -0.2712147131 -0.7145434768 5.0733999299
C 0.2712147137 0.714543476 5.0733999301
C -0.1503923283 1.4621827495 3.8091953579
H 1.2474905851 -1.573046544 1.2693885358
C 0.1474519994 -1.4563147851 -1.269880216
C -0.1474519999 1.4563147857 -1.2698802165
H -1.2474905855 1.5730465446 1.2693885366
H 1.2496214953 -1.5766743401 3.8010052849
H -1.3735141189 -0.6816440103 5.1211290968
H 1.3735141195 0.6816440096 5.1211290967
H -1.2496214949 1.5766743399 3.8010052858
C 0.2712288393 0.7188193378 -2.5407398701
C -0.2712288395 -0.7188193377 -2.54073987
C -0.1503923253 1.4621827496 -3.809195358
C 0.2712147152 0.7145434755 -5.0733999303
C -0.2712147146 -0.7145434762 -5.0733999301
C 0.1503923256 -1.4621827498 -3.8091953575
H -0.2754655721 -2.4749430574 -1.2699823938
H 0.2754655716 2.474943058 -1.2699823947
H -1.2496214918 1.5766743421 -3.801005286
H 1.249621492 -1.5766743422 -3.8010052851
H -0.2754655682 -2.4749430591 1.2699823942
H -1.3778424194 -0.643093757 0.0000000012
H 1.3778424185 0.6430937581 0.0000000012
H 0.2754655677 2.4749430598 1.2699823951
H 1.3772551514 0.6451253904 2.542040964
H -1.3772551515 -0.6451253903 2.5420409643
H -0.2711574791 -2.4800867856 3.8025323364

H 0.0673108253 -1.2549350066 5.970339782
H -0.0673108245 1.2549350055 5.9703397825
H 0.2711574795 2.4800867853 3.8025323372
H 1.2474905821 -1.5730465434 -1.2693885376
H -1.2474905826 1.573046544 -1.2693885385
H 1.3772551528 0.6451253878 -2.5420409641
H -1.377255153 -0.6451253877 -2.5420409644
H 0.2711574844 2.4800867847 -3.802532337
H 1.3735141209 0.6816440069 -5.1211290968
H -1.3735141203 -0.6816440076 -5.1211290969
H -0.271157484 -2.4800867849 -3.8025323363
H -0.067310822 1.2549350057 -5.9703397826
H 0.0673108228 -1.2549350067 -5.9703397822

11
42
0 1
C 0.2222699388 -2.5228444095 0.7295949859
C -0.2222699393 -2.5228444091 -0.7295949854
C 0.2499267608 -1.2691873852 -1.4642899922
C -0.2169220337 -0.0000000009 -0.7362241383
C 0.2169220364 -0.0000000009 0.7362241369
C -0.2499267602 -1.2691873845 1.4642899923
C -0.197992353 -1.2576299991 -2.9258961698
C 0.2712583822 0.0000000004 -3.653739682
C -0.197992355 1.2576299992 -2.9258961693
C 0.2499267591 1.2691873853 -1.4642899917
H 1.3253141261 -2.564244195 0.776228918
H -0.1487265967 -3.4213000507 1.2480636271
H -1.3253141267 -2.5642441931 -0.7762289169
H 0.1487265948 -3.4213000508 -1.2480636269
H 1.3580643622 -1.2623333272 -1.4472534087
H -1.3277221291 -0.0000000012 -0.7539430569
C -0.2499267585 1.2691873846 1.4642899917
C 0.197992353 -1.257629999 2.92589617
H -1.3016100676 -1.3068335006 -2.9553185869
H 0.1708805493 -2.1619089634 -3.4357850103
H -0.0888306223 0.0000000002 -4.6936019437
H 1.373765502 0.0000000011 -3.7002799624
H -1.3016100696 1.3068334996 -2.9553185862
H 0.1708805464 2.161908964 -3.4357850095
C -0.2222699405 2.5228444088 -0.7295949849
H 1.3277221318 -0.0000000012 0.7539430556
H -1.3580643615 -1.2623333251 1.4472534082
H 1.3580643605 1.2623333287 -1.4472534085
H -1.3580643598 1.2623333266 1.447253408
C 0.197992355 1.2576299991 2.9258961695
H 1.3016100676 -1.306833502 2.9553185873
H -0.1708805505 -2.1619089628 3.4357850105
C -0.2712583817 0.0000000004 3.6537396816
H -1.3253141279 2.5642441926 -0.7762289155
H 0.148726593 3.4213000506 -1.2480636268
C 0.2222699399 2.5228444092 0.7295949854
H -0.1708805476 2.1619089634 3.4357850097
H 1.3016100695 1.306833501 2.9553185866
H 0.088830622 0.0000000002 4.6936019436
H -1.3737655016 0.0000000011 3.7002799613
H -0.1487265949 3.4213000505 1.248063627
H 1.3253141273 2.5642441945 0.7762289166

9a
18
0 1
C 1.2623943438 0.7288425811 0.2318676307
C 1.2623943438 -0.7288425811 -0.2318676307
C 0. -1.4576817735 0.2318568498
C -1.2623943438 -0.7288425811 -0.2318676307
C -1.2623943438 0.7288425811 0.2318676307
C 0. 1.4576817735 -0.2318568498
H 2.161701116 1.2480818419 -0.1327879713
H 1.308161501 0.7552393568 1.3348083094
H 2.161701116 -1.2480818419 0.1327879713
H 1.308161501 -0.7552393568 -1.3348083094
H 0. -1.5104306055 1.3347907901
H 0. -2.4961186944 -0.1328186408
H -2.161701116 -1.2480818419 0.1327879713
H -1.308161501 -0.7552393568 -1.3348083094
H -1.308161501 0.7552393568 1.3348083094
H -2.161701116 1.2480818419 -0.1327879713
H 0. 2.4961186944 0.1328186408
H 0. 1.5104306055 -1.3347907901

9b
27
0 1
C -0.7239675881 -1.2539482548 0.1281382574
C -1.4719174321 0. -0.3302913315
C -0.7239675881 1.2539482548 0.1281382574
C 0.7359311772 1.2747751335 -0.3303268213
C 1.4479279244 0. 0.1279202445
C 0.7359311772 -1.2747751335 -0.3303268213
H -0.7492570736 1.2978694318 1.233943904
H 0.7381590651 1.2789018796 -1.4360486421
H 1.4986693932 0. 1.2337096409
H 0.7381590651 -1.2789018796 -1.4360486421
H -0.7492570736 -1.2978694318 1.233943904
H -1.4762800155 0. -1.4360179929
H -1.2450494153 2.1564580373 -0.2327780945
C 1.4584628632 2.5260927164 0.1571365796
H 2.4900144358 0. -0.2331686223

C 1.4584628632 -2.5260927164 0.1571365796
H -1.2450494153 -2.1564580373 -0.2327780945
C -2.9169392479 0. 0.1568912374
H 2.500056716 2.5501028332 -0.195615277
H 1.4758207308 2.5553725497 1.2582089695
H 0.958036843 3.4401919038 -0.1949107661
H 0.958036843 -3.4401919038 -0.1949107661
H 1.4758207308 -2.5553725497 1.2582089695
H 2.500056716 -2.5501028332 -0.195615277
H -2.9511491972 0. 1.2579504315
H -3.4583562486 -0.8902553606 -0.1955799135
H -3.4583562486 0.8902553607 -0.1955799135

9c
54
0 1
C -0.7348251397 -1.2609273319 0.0605425454
C -1.46521576 0.0000000016 -0.4193408211
C -0.7348251367 1.2609273332 0.0605425461
C 0.7412486808 1.2715260419 -0.35518408
C 1.4150152173 -0.0000000019 0.1708122584
C 0.741248678 -1.2715260439 -0.3551840805
H -0.8003392752 1.3279992819 1.1613342964
H 0.7613005358 1.224880085 -1.4615756757
H 1.3482994771 -0.0000000022 1.2722045961
H 0.7613005336 -1.2248800864 -1.4615756763
H -0.8003392785 -1.3279992808 1.1613342956
H -1.398307726 0.0000000025 -1.5252225612
H -1.2380429733 2.1462222666 -0.3476677008
C 1.5015883962 2.5762454748 0.0268549734
H 2.4841384088 -0.0000000035 -0.0840635496
C 1.501588392 -2.5762454776 0.0268549728
H -1.2380429791 -2.1462222636 -0.3476677019
C -2.9894454133 0.0000000018 -0.0966823017
C 2.8404101897 2.6248266789 -0.722842082
C 1.7738432258 2.658570057 1.5341390826
C 0.6889515178 3.8079756011 -0.3955552619
C 0.6889515217 -3.8079756043 -0.3955552771
C 1.7738432077 -2.6585700671 1.5341390841
C 2.840410193 -2.6248266728 -0.7228420696
C -3.2626446732 0.0000000083 1.4127144563
C -3.6515832441 -1.2394656944 -0.7160047967
C -3.6515832457 1.2394656928 -0.7160048063
H 3.4939176133 1.7809874845 -0.4605658641
H 3.3829457525 3.5502093518 -0.4761433578
H 2.6799322954 2.6074218906 -1.8120667671
H 2.4753929512 1.8785764785 1.8638504413
H 0.8473939878 2.5571740557 2.1198970228
H 2.220271382 3.6332786715 1.7843188134
H 0.3667534684 3.7287205756 -1.4462383149
H 1.3013118037 4.7170957625 -0.297474298
H -0.2062087757 3.944118555 0.227655894
H -0.2062087767 -3.9441185653 0.2276558701
H 1.3013118101 -4.717095764 -0.2974743132
H 0.3667534814 -3.7287205737 -1.4462383325
H 0.8473939638 -2.5571740713 2.1198970161
H 2.4753929277 -1.8785764884 1.8638504536
H 2.2202713641 -3.6332786817 1.784318814
H 2.6799323094 -2.6074218814 -1.8120667562
H 3.3829457579 -3.550209344 -0.4761433433
H 3.4939176098 -1.7809874761 -0.4605658422
H -2.844393888 -0.8913756608 1.9029430591
H -4.3479555408 0.0000000122 1.5977501458
H -2.8443938863 0.8913756815 1.9029430502
H -3.3339808248 -2.1685917044 -0.2226551761
H -3.4120955919 -1.3207736674 -1.7882398048
H -4.745749051 -1.1742331844 -0.6181290291
H -3.3339808311 2.1685917066 -0.2226551899
H -4.7457490527 1.17423318 -0.6181290421
H -3.4120955901 1.3207736603 -1.7882398141

9e
66
0 1
C 1.2325890312 5.3827346355 1.5620669502
C 1.103146065 5.1180298638 0.0621906975
C 1.1765976016 3.6215205222 -0.2432048802
C 0.1165603503 2.8303537813 0.5331729396
C 0.2727814948 3.0971258312 2.0359498648
C 0.189167723 4.5919277505 2.3506997379
C 0.0960771549 1.339259854 0.1500706888
C 1.3313566553 0.5549280791 0.6061678918
C 1.3004211373 -0.8941434074 0.1074729607
C 0.0161853252 -1.57681101 0.5907538483
C -1.2376698009 -0.8212865285 0.1362690331
C -1.172449207 0.6270269759 0.6332436415
C 2.5904296412 -1.6633886508 0.4471403709
C 2.7250872025 -2.9636047269 -0.3548748012
C 4.0623932607 -3.6569790752 -0.0914661729
C 4.2602398662 -3.9272333005 1.3998581429
C 4.1196824057 -2.6411680793 2.2135628782
C 2.7782477413 -1.9574693839 1.9410763757
C -2.5364560775 -1.5597005042 0.5089403929
C -3.7567066641 -1.0236503489 -0.250091048
C -5.0128271186 -1.8442273171 0.0454740151
C -5.2986617565 -1.8970181445 1.5461676342
C -4.0869102356 -2.4194222813 2.3173393022
C -2.8379801049 -1.590037423 2.0128223871
H 1.2487844541 -0.8506620125 -0.9972618886
H 2.2537765079 1.0445560298 0.2534558963

H 1.3691731801 0.5561876407 1.7094443396
H 0.0807325861 1.3050055487 -0.9561107119
H -2.0592316737 1.1914723608 0.3014091629
H -1.1868153169 0.627116765 1.7369744511
H -1.2077010146 -0.7869724019 -0.969581674
H -0.0306048489 -2.6165654268 0.2278579366
H 0.0289004897 -1.6223466768 1.693558456
H 2.6083351957 -2.7514441516 -1.4298274488
H 1.9066398168 -3.6505912236 -0.0768625964
H 2.699421743 -1.0307601689 2.5305633636
H 1.9662722616 -2.6227158561 2.2833966792
H 1.0558602155 3.4441575763 -1.3240360619
H 2.1807045374 3.2511466295 0.0283627205
H -0.4907591455 2.5474083463 2.6079728068
H 1.2548491061 2.7190725598 2.3702014972
H -3.5485674049 -1.0173355114 -1.332151131
H -3.9381109698 0.0251570533 0.0436827296
H -1.9761302236 -1.9853145843 2.5725937103
H -3.0030669435 -0.5581876399 2.3691532875
H 4.1264538547 -4.5946907072 -0.6641082608
H 4.2276614261 -2.8529762865 3.2881795712
H 3.4989382171 -4.651679526 1.7385814588
H 5.2410516141 -4.3928077653 1.580504671
H -5.8766626908 -1.4299680394 -0.4962941111
H -4.2905891675 -2.4183593436 3.3989307569
H -5.540048304 -0.8802517369 1.902577803
H -6.1829112153 -2.5203935837 1.7483431841
H 1.8834083782 5.6606264828 -0.4928905735
H 0.3147651859 4.7613979573 3.4309056737
H 2.2405364306 5.0794541136 1.8954934064
H 1.1412200437 6.4591002687 1.7728842581
H -0.8675727799 3.2406429449 0.2340937443
H 0.1335178246 5.5104400981 -0.2908130167
H -0.8179284414 4.9602110893 2.0882426548
H 3.4310064534 -1.0117416482 0.1387999006
H 4.8808663368 -3.0099451966 -0.4523129658
H 4.9382338726 -1.9512299254 1.9438931397
H -2.3992348328 -2.6102323709 0.1867402614
H -4.8677468679 -2.8721314305 -0.3301775916
H -3.9016945814 -3.4696583625 2.0319026597

9f

48
0 1
C -0.7157917607 1.2883474942 -0.2366449746
C 0.7771336604 1.2756882619 -0.5920220953
C 1.4347147659 -0.0179114458 -0.0921926831
C 0.7265893526 -1.265623002 -0.6372239768
C -0.7659614828 -1.2310543562 -0.28141236
C -1.4434351015 0.051912059 -0.7819498186
H -1.1875637696 2.2044428542 -0.623728666
H -0.8260693446 1.3176989101 0.8616849262
H 0.8543720596 1.280904832 -1.6936689696
H 1.4029862844 -0.036895082 1.011415639
H 2.4982001399 -0.03396779 -0.3756919206
H 0.8044701134 -1.2348880942 -1.7384036472
H -0.8776052805 -1.295000533 0.8152650944
H -1.2737191609 -2.113315885 -0.7002770677
H -1.3458221651 0.0694716413 -1.8818412926
C -2.9225654319 0.0772471231 -0.4596600618
C -3.36992101 0.0587143724 0.8681532208
H -2.6440949427 0.0239473479 1.6843262377
C -4.7302516497 0.0842801847 1.1660867554
H -5.057427118 0.0693104917 2.2070281665
C -5.6727364085 0.1290691696 0.1383061393
H -6.7384590149 0.1493350977 0.3707602749
C -5.2421423988 0.1478604752 -1.1863406526
H -5.9703339109 0.1829419488 -1.9983389066
C -3.8783743481 0.122081466 -1.4792038257
H -3.5470067714 0.1372303135 -2.5202188757
C 1.3905942717 -2.5449658715 -0.1739280313
C 1.9561416175 -3.4328178215 -1.0942671423
H 1.9157023746 -3.1935893191 -2.1595751363
C 2.567631994 -4.6136277199 -0.6725789285
H 3.0024177987 -5.2919229098 -1.4085935733
C 2.6229433393 -4.925848824 0.6839112406
H 3.1002482958 -5.8485986414 1.0169756993
C 2.0629412208 -4.0492384955 1.613749378
H 2.1011904586 -4.284921289 2.6785708955
C 1.4535977255 -2.8715936131 1.1873316074
H 1.0192690038 -2.1952892661 1.9277191018
C 1.4928924175 2.5093941848 -0.0842169939
C 1.5697609663 2.783980638 1.2877719842
H 1.1071499141 2.1004399928 2.0040225029
C 2.228984674 3.918415365 1.7552245478
H 2.2775399256 4.1136734619 2.8277763632
C 2.8259570554 4.8031371513 0.8565816818
H 3.3426749048 5.6918273408 1.2218754809
C 2.7570581984 4.5426019426 -0.5101488773
H 3.2204251648 5.2276778774 -1.2220620294
C 2.0956243966 3.404897089 -0.9729776634
H 2.045128977 3.2059888721 -2.0461157673

9d

90
0 1
C 0.7212877745 1.2709678128 -0.5592749317
C -0.7563949098 1.2641032005 -0.1477954179
C -1.4882878602 -0.0000015532 -0.6223059335
C -0.7563943079 -1.2641064077 -0.1477962333

C 0.7212888529 -1.2709698685 -0.5592744504
C 1.3868102097 -0.0000006132 -0.0213792175
C 1.491003003 2.5706507437 -0.1912987254
H 0.7431699444 1.2152287822 -1.665371771
H -1.2555730536 2.143963371 -0.5693492773
H -0.8257892403 1.3447440752 0.9520535288
H -1.4233278151 -0.000000986 -1.7286964283
C -3.0105586339 -0.000001065 -0.3014460396
H -1.2555712836 -2.1439664926 -0.5693514921
H -0.8257895921 -1.344748568 0.9520525742
H 0.7431718482 -1.2152316368 -1.6653713123
C 1.4910048004 -2.5706519135 -0.1912966806
H 2.4614359653 -0.0000001583 -0.2515721655
H 1.2954222956 -0.0000004422 1.0776784941
C 1.8156943249 2.6693158326 1.3136393602
C 0.680932248 3.8244387537 -0.5850160383
C 2.8242905367 2.6174552667 -0.9710502426
C -3.3102682448 -0.0000082602 1.2120542015
C -3.6858150327 -1.2455871634 -0.9180925365
C -3.6858097341 1.2455942793 -0.9180794895
C 1.8156954285 -2.6693152265 1.3136416669
C 2.8242927507 -2.6174563819 -0.9710474972
C 0.680935046 -3.8244408437 -0.5850130923
H 0.8830901574 2.638164849 1.9028802035
C 2.5888131486 3.9610714683 1.6241317011
H 2.4249059807 1.8052749433 1.6234771464
H -0.2626722848 3.8540816491 -0.0189248809
H 0.4167323501 3.7683845235 -1.656135534
C 1.4614135193 5.1131400332 -0.2921224572
H 3.4429073048 1.742239559 -0.7173907636
C 3.6124734445 3.8972969271 -0.6626064483
H 2.6112159892 2.5598601919 -2.0524302409
H -2.8592805255 -0.8873031302 1.6860125984
C -4.8264724541 -0.0000066044 1.4693088607
H -2.8592771654 0.8872800319 1.6860216832
H -3.260903686 -2.1612653423 -0.4799065599
H -3.4746397501 -1.2748165785 -2.0016552063
C -5.2000720433 -1.2481066528 -0.670879102
H -3.2608939017 2.1612660761 -0.4798843993
C -5.2000666608 1.2481179556 -0.6708656727
H -3.4746346968 1.2748337377 -2.0016419245
H 2.4249063143 -1.8052735799 1.6234788736
C 2.5888149654 -3.9610700099 1.6241357538
H 0.8830909371 -2.638164287 1.9028819991
H 3.4429088467 -1.7422400306 -0.7173886151
H 2.6112187072 -2.559862563 -2.0524276625
C 3.6124763267 -3.8972972163 -0.6626019685
H -0.2626697664 -3.8540837477 -0.0189223971
C 1.4614170029 -5.1131413134 -0.2921177417
H 0.4167356711 -3.7683879031 -1.6561327831
C 3.9094512151 3.9615098023 0.8418347677
C 2.7801946028 5.1174947422 -1.0738045818
C 1.7556025438 5.1817756684 1.2125715381
H 2.7997413431 4.0045389934 2.7042186877
H 0.8509487705 5.9802461853 -0.5895209547
H 4.5584549964 3.8855964491 -1.2264748714
H 4.5256297469 3.0978861897 1.1419124305
H 4.4872668887 4.8709142622 1.0753168641
H 2.5777011395 5.0879910798 -2.1570475254
H 3.3385609896 6.0462001168 -0.8707884786
H 0.8109138373 5.2036201408 1.7805339831
H 2.3011997128 6.1095249274 1.4513437638
C 2.7801984734 -5.1174960066 -1.0737992107
C 3.9094534089 -3.9615083024 0.8418394621
C 1.7556053436 -5.181775175 1.2125764721
H 2.7997426579 -4.0045362485 2.7042228899
H 4.5584581467 -3.8855967246 -1.2264699398
H 0.8509529569 -5.9802481738 -0.5895156151
H 2.5777055284 -5.0879936332 -2.1570422857
H 3.3385653568 -6.0462008056 -0.8707818377
H 4.5256312222 -3.0978839614 1.1419165045
H 4.487269569 -4.8709121322 1.0753228091
H 0.8109163733 -5.2036196443 1.7805384774
H 2.3012029879 -6.1095238327 1.4513499519
C -5.4572401535 -1.2506577893 0.8423874328
C -5.4572347893 1.2506539891 0.8424008995
C -5.8305149411 0.0000104019 -1.2980740877
H -5.0075402813 -0.0000120423 2.555668142
H -5.6380731236 -2.1524162338 -1.1218531022
H -5.6380637885 2.1524342767 -1.1218299986
H -5.0269021499 -2.1587460507 1.2959161322
H -6.541120658 -1.2681819951 1.0431669699
H -5.0268928976 2.1587355392 1.2959393435
H -6.5411152174 1.2681806637 1.043180632
H -5.6643673645 0.0000159077 -2.3879219141
H -6.9206654984 0.0000118923 -1.1335290654

Frozen dimers

7
60

C 3.700000 0.722998 -2.444695
C 3.700000 -0.722998 -2.444695
C 3.700000 -1.409433 -3.708472
C 3.700000 -0.717424 -4.881388
C 3.700000 0.717424 -4.881388
C 3.700000 1.409433 -3.708472
C 3.700000 1.405151 -1.234680
C 3.700000 -1.405151 -1.234680
H 3.700000 -2.501242 -3.705358
H 3.700000 -1.251805 -5.832401
H 3.700000 1.251805 -5.832401
H 3.700000 2.501242 -3.705358
C 3.700000 -0.722351 0.000000
C 3.700000 0.722351 0.000000
H 3.700000 2.497866 -1.235375
H 3.700000 -2.497866 -1.235375
C 3.700000 -1.405151 1.234680
C 3.700000 1.405151 1.234680
C 3.700000 0.722998 2.444695
C 3.700000 -0.722998 2.444695
H 3.700000 -2.497866 1.235375
H 3.700000 2.497866 1.235375
C 3.700000 1.409433 3.708472
C 3.700000 -1.409433 3.708472
C 3.700000 0.717424 4.881388
C 3.700000 -0.717424 4.881388
H 3.700000 2.501242 3.705358
H 3.700000 -2.501242 3.705358
H 3.700000 1.251805 5.832401
H 3.700000 -1.251805 5.832401
C 0.0000000013 0.7229984214 -2.4446946599
C -0.0000000013 -0.7229984214 -2.4446946599
C 0. -1.4094335199 -3.7084720514
C -0.0000000044 -0.7174238937 -4.8813880483
C 0.0000000045 0.7174238937 -4.8813880483
C -0.0000000007 1.4094335199 -3.7084720514
C 0.0000000002 1.4051504752 -1.2346796945
C 0.0000000002 -1.4051504752 -1.2346796945
H -0.0000000004 -2.5012416025 -3.7053575762
H -0.0000000041 -1.2518046335 -5.8324011925
H 0.0000000045 1.2518046335 -5.8324011925
H -0.0000000002 2.5012416025 -3.7053575762
C -0.000000001 -0.7223507702 0.
C 0.0000000013 0.7223507702 0.
H 0.0000000007 2.497865981 -1.2353750297
H -0.0000000005 -2.497865981 -1.2353750297
C 0.0000000017 -1.4051504752 1.2346796945
C -0.0000000013 1.4051504752 1.2346796945
C 0.0000000004 0.7229984214 2.4446946599
C 0.0000000001 -0.7229984214 2.4446946599
H 0.0000000008 -2.497865981 1.2353750297
H -0.0000000009 2.497865981 1.2353750297
C -0.0000000014 1.4094335199 3.7084720514
C 0.0000000012 -1.4094335199 3.7084720514
C 0.0000000009 0.7174238937 4.8813880483
C -0.0000000008 -0.7174238937 4.8813880483
H -0.000000003 2.5012416025 3.7053575762
H 0.0000000026 -2.5012416025 3.7053575762
H 0.0000000007 1.2518046335 5.8324011925
H -0.0000000009 -1.2518046335 5.8324011925

8
52

C 3.600000 -2.464608 0.678411
C 3.600000 -2.464608 -0.678411
C 3.600000 -1.232235 -1.427657
C 3.600000 0.000000 -0.714818
C 3.600000 0.000000 0.714818
C 3.600000 -1.232235 1.427657
C 3.600000 -1.209309 -2.828967
C 3.600000 0.000000 -3.519875
C 3.600000 1.209309 -2.828967
C 3.600000 1.232235 -1.427657
H 3.600000 -3.405763 1.232048
H 3.600000 -3.405763 -1.232048
C 3.600000 1.232235 1.427657
C 3.600000 -1.209309 2.828967
H 3.600000 -2.154973 -3.374682
H 3.600000 0.000000 -4.610834
H 3.600000 2.154973 -3.374682
C 3.600000 2.464608 -0.678411
C 3.600000 1.209309 2.828967
H 3.600000 -2.154973 3.374682
C 3.600000 0.000000 3.519875
H 3.600000 3.405763 -1.232048
C 3.600000 2.464608 0.678411
H 3.600000 2.154973 3.374682
H 3.600000 0.000000 4.610834
H 3.600000 3.405763 1.232048
C -0.0000000013 -2.4646083636 0.6784110538
C -0.0000000015 -2.4646083636 -0.6784110538
C 0.0000000002 -1.2322347528 -1.427657105
C -0.0000000014 0. -0.7148181042
C -0.0000000008 0. 0.7148181042

C -0.0000000002 -1.2322347528 1.427657105
C -0.0000000011 -1.2093090131 -2.8289670289
C 0.0000000037 0. -3.5198747389
C -0.0000000015 1.2093090131 -2.8289670289
C 0.0000000002 1.2322347528 -1.427657105
H -0.0000000008 -3.4057630201 1.2320478493
H -0.0000000008 -3.4057630201 -1.2320478493
C -0.0000000001 1.2322347528 1.427657105
C -0.0000000011 -1.2093090131 2.8289670289
H 0.0000000018 -2.1549726742 -3.3746816086
H 0.0000000012 0. -4.6108342634
H 0.0000000015 2.1549726742 -3.3746816086
C -0.000000002 2.4646083636 -0.6784110538
C -0.0000000007 1.2093090131 2.8289670289
H 0.0000000012 -2.1549726742 3.3746816086
C 0.000000003 0. 3.5198747389
H -0.0000000009 3.4057630201 -1.2320478493
C -0.0000000008 2.4646083636 0.6784110538
H 0.0000000016 2.1549726742 3.3746816086
H 0.0000000014 0. 4.6108342634
H -0.0000000008 3.4057630201 1.2320478493

6a
24

C 1.207961 -0.697421 3.900000
C 1.207961 0.697421 3.900000
C 0.000000 1.394826 3.900000
C -1.207961 0.697421 3.900000
C -1.207961 -0.697421 3.900000
C 0.000000 -1.394826 3.900000
H 2.152891 -1.242936 3.900000
H 2.152891 1.242936 3.900000
H 0.000000 2.485924 3.900000
H -2.152891 1.242936 3.900000
H -2.152891 -1.242936 3.900000
H 0.000000 -2.485924 3.900000
C 1.2079608624 -0.6974210355 0.
C 1.2079608624 0.6974210355 0.
C 0. 1.3948256098 0.
C -1.2079608624 0.6974210355 0.
C -1.2079608624 -0.6974210355 0.
C 0. -1.3948256098 0.
H 2.1528905787 -1.2429361064 0.
H 2.1528905787 1.2429361064 0.
H 0. 2.485924516 0.
H -2.1528905787 1.2429361064 0.
H -2.1528905787 -1.2429361064 0.
H 0. -2.485924516 0.

6b
42

C 0.701400 1.200103 3.600000
C 1.417980 -0.005786 3.599940
C 0.706396 -1.202965 3.600040
C -0.696195 -1.220603 3.600000
C -1.377211 -0.005698 3.599960
C -0.691187 1.217751 3.600020
H 1.252235 -2.150824 3.600170
C -1.435126 -2.533929 3.599920
H -2.470990 -0.004579 3.600000
C -1.459157 2.514347 3.600580
H 1.249333 2.146730 3.600090
C 2.924841 0.011015 3.598890
H -2.522140 -2.381212 3.601370
H -1.173468 -3.132745 4.484860
H -1.175692 -3.131357 2.713370
H -0.783475 3.379424 3.596850
H -2.104272 2.589076 4.488530
H -2.110582 2.586206 2.717050
H 3.336144 -1.006648 3.608180
H 3.313107 0.544252 4.479240
H 3.311803 0.527153 2.707760
C   1.397595   0.000000   0.000000
C   0.711555   -1.223520   -0.000060
C   -0.681025   -1.205860   0.000040
C   -1.397595   0.000000   0.000000
C   -0.685965   1.197230   -0.000040
C   0.716585   1.214840   0.000020
H   -1.228975   -2.152500   0.000170
C   -2.904435   -0.016730   -0.000080
H   -1.231885   2.145030   0.000000
C   1.455485   2.528220   0.000580
H   2.491365   -0.001210   0.000090
C   1.479535   -2.520100   -0.001110
H   -3.315685   1.001010   0.001370
H   -3.292195   -0.542740   0.884860
H   -3.292105   -0.540120   -0.886630
H   2.542505   2.375600   -0.003150
H   1.197645   3.124270   0.888530
H   1.192005   3.128300   -0.882950
H   0.803865   -3.385130   0.008180
H   2.135465   -2.589730   0.879240
H   2.120005   -2.597150   -0.892240

6c
96

C 0.702354 1.197579 4.900000

C 1.425514 -0.008910 4.899960
C 0.710055 -1.202063 4.899700
C -0.696386 -1.225110 4.900000
C -1.371836 -0.008824 4.900260
C -0.688691 1.220699 4.900020
H 1.246850 -2.150646 4.899360
C -1.421468 -2.576028 4.899710
H -2.461695 0.000806 4.900390
C -1.496183 2.523981 4.899460
H 1.255762 2.136530 4.899900
C 2.957933 0.039056 4.900160
C -1.019274 -3.368669 6.154240
C -1.020620 -3.366979 3.643610
C -2.943962 -2.413265 4.900650
C -0.593849 3.760969 4.900290
C -2.383738 2.572072 6.154040
C -2.381666 2.572180 3.643430
C 3.442380 0.781583 6.156310
C 3.442666 0.784339 3.645770
C 3.578614 -1.360672 4.898630
H 0.063527 -3.555823 6.185090
H -1.531486 -4.343125 6.169660
H -1.294074 -2.818015 7.065990
H 0.062206 -3.553871 3.611310
H -1.296508 -2.814912 2.733030
H -1.532761 -4.341454 3.627320
H -3.291913 -1.872235 5.792930
H -3.420972 -3.404292 4.900820
H -3.293025 -1.872020 4.008920
H 0.048042 3.792197 5.793010
H -1.213414 4.669710 4.899440
H 0.050108 3.791975 4.009050
H -1.769375 2.534958 7.065750
H -3.087007 1.727714 6.184930
H -2.971735 3.502770 6.169240
H -1.765867 2.535034 2.732700
H -2.969561 3.502935 3.627210
H -3.084991 1.727924 3.611300
H 3.102442 0.266493 7.066900
H 3.062625 1.812727 6.188780
H 4.542369 0.825479 6.172430
H 3.062210 1.815288 3.615180
H 3.103561 0.270812 2.733990
H 4.542632 0.829034 3.630350
H 3.284719 -1.934053 5.790140
H 4.675361 -1.278011 4.898250
H 3.283966 -1.932198 4.006180
C 1.398740 0.000000 0.000000
C 0.715470 -1.229520 -0.000040
C -0.675560 -1.206490 -0.000300
C -1.398740 0.000000 0.000000
C -0.683130 1.193100 0.000260
C 0.723240 1.216240 0.000020
H -1.228660 -2.145660 -0.000640
C -2.931210 -0.047520 -0.000290
H -1.219720 2.141760 0.000390
C 1.448170 2.567190 -0.000540
H 2.488600 -0.009790 -0.000100
C 1.523220 -2.532650 0.000160
C -3.416560 -0.792150 1.254240
C -3.415770 -0.790140 -1.256390
C -3.551500 1.352380 0.000650
C 2.970600 2.404240 0.000290
C 1.046040 3.359880 1.254040
C 1.047170 3.358140 -1.256570
C 2.408490 -2.580930 1.256310
C 2.411020 -2.579800 -1.254230
C 0.621360 -3.770040 -0.001370
H -3.037240 -1.823460 1.285090
H -4.516570 -0.835790 1.269660
H -3.077080 -0.278840 2.165990
H -3.036210 -1.821340 -1.288690
H -3.075610 -0.275180 -2.166970
H -4.515760 -0.833850 -1.272680
H -3.256930 1.924230 0.892930
H -4.648260 1.269970 0.000820
H -3.257300 1.925300 -0.891080
H 3.318590 1.863960 0.893010
H 3.447810 3.395170 -0.000560
H 3.319430 1.862060 -0.890950
H 1.321080 2.809270 2.165750
H -0.036830 3.546750 1.284930
H 1.558050 4.334450 1.269240
H 1.322900 2.806270 -2.167300
H 1.559280 4.332650 -1.272790
H -0.035640 3.545110 -1.288700
H 1.792440 -2.544080 2.166900
H 3.111610 -1.736480 1.288780
H 2.996500 -3.511600 1.272430
H 3.113620 -1.734840 -1.284820
H 1.796740 -2.542890 -2.166010
H 2.999710 -3.510050 -1.269650
H -0.022150 -3.802210 0.890140
H 1.241320 -4.678520 -0.001750
H -0.020920 -3.800630 -0.893820

6e
120

C -0.005343 1.408983 4.501420

C -1.202452 0.693640 4.502294
C -1.219027 -0.708389 4.501433
C -0.000971 -1.387348 4.500000
C 1.221529 -0.700814 4.499360
C 1.200451 0.693576 4.500000
C -0.006612 2.922757 4.506526
C -2.528892 -1.467015 4.504386
C 2.533241 -1.456318 4.499385
C 2.709233 -2.313549 5.762671
C 4.050677 -3.046428 5.762955
C 4.216702 -3.899584 4.505415
C 4.047005 -3.055146 3.242494
C 2.705622 -2.322108 3.241571
C -3.352567 -1.198115 5.773657
C -4.656867 -1.995203 5.776971
C -5.486197 -1.707546 4.525417
C -4.676463 -1.974983 3.256667
C -3.372360 -1.177552 3.252918
C 0.665076 3.510835 3.255748
C 0.630293 5.039066 3.264267
C 1.269597 5.601254 4.533945
C 0.605519 5.025303 5.784605
C 0.639881 3.497205 5.776875
H -2.146346 1.246638 4.504143
H 2.151315 1.234505 4.499877
H -0.007933 -2.481293 4.499721
H -2.781166 -1.397644 2.350871
H -3.605536 -0.098706 3.221371
H -2.747404 -1.433519 6.662488
H -3.583653 -0.119586 5.826489
H 0.132941 3.089446 6.664788
H 1.688712 3.155631 5.828414
H 1.715050 3.170848 3.221043
H 0.176188 3.112906 2.353360
H 1.886487 -3.061537 3.202647
H 2.603539 -1.692919 2.344163
H 1.890039 -3.052561 5.808924
H 2.609712 -1.678048 6.655913
H -5.270876 -1.735031 2.362131
H -5.237416 -1.769927 6.684345
H -6.407806 -2.308947 4.527882
H -5.799100 -0.648729 4.536380
H 1.093061 5.411525 6.692489
H 1.135354 5.435268 2.370375
H 1.212706 6.700243 4.539329
H 2.342322 5.339878 4.543038
H 4.136977 -3.683619 2.343592
H 4.143436 -3.668609 6.665948
H 5.197462 -4.398744 4.505675
H 3.454837 -4.698758 4.509348
H -1.061052 3.252190 4.497694
H 3.345559 -0.707598 4.495855
H -0.445069 5.362483 5.823220
H -0.419447 5.376541 3.208760
H 4.863439 -2.313678 3.191747
H 4.867161 -2.304538 5.806155
H -2.286121 -2.544704 4.493759
H -4.441009 -3.052102 3.199104
H -4.420613 -3.073063 5.813456

C	1.21808530	0.70828250	0.00142009
C	0.00002579	1.38733807	0.00229369
C	-1.22245461	0.70067786	0.00143263
C	-1.20142243	-0.69366904	0.00000000
C	0.00438384	-1.40911769	-0.00063953
C	1.20142243	-0.69366904	0.00000000
C	2.52841816	1.46626876	0.00652589
C	-2.53437629	1.45574128	0.00438642
C	0.00595431	-2.92284632	-0.00061536
C	-0.64843375	-3.50387479	1.26267120
C	-0.61240292	-5.03203952	1.26295541
C	-1.26824589	-5.60239987	0.00541482
C	-0.62179007	-5.03321820	-1.25750646
C	-0.65765162	-3.50502795	-1.25842884
C	-2.71333911	2.30351483	1.27365664
C	-4.05578784	3.03452804	1.27697143
C	-4.22133426	3.89657662	0.02541729
C	-4.04807464	3.06160881	-1.24333283
C	-2.70542783	2.33093684	-1.24708226
C	3.37355208	1.17860899	-1.24425200
C	4.67964732	1.97284753	-1.23573260
C	5.48616953	1.70028766	0.03394461
C	4.65534222	1.98742132	1.28460504
C	3.34915110	1.19361343	1.27687539
H	0.00698954	2.48127284	0.00414337
H	2.14531176	-1.24667711	-0.00012326
H	-2.15228741	-1.23461269	-0.00027944
H	-2.60043609	1.70890253	-2.14912940
H	-1.88770769	3.07229639	-1.27862867
H	-2.61462401	1.66172652	2.16248807
H	-1.89484909	3.04290515	1.32648890
H	2.74255129	1.42875718	2.16478844
H	3.57775513	0.11451189	1.32841435
H	3.60410214	0.09931063	-1.27895732
H	2.78449140	1.40303354	-2.14663989
H	-1.70758352	-3.16535059	-1.29735308
H	-0.16379947	-3.10202728	-2.15583648
H	-1.69803421	-3.16393840	1.30892411
H	-0.14783390	-3.09993668	2.15591324
H	-4.13747680	3.69636074	-2.13786853
H	-4.15096828	3.64993637	2.18434467

H -5.20296819 4.39401348 0.02788177
H -3.46082370 4.69696735 0.03638008
H 5.23359089 1.75830822 2.19248866
H 5.27529935 1.73355271 -2.12962506
H 6.40947560 2.29905147 0.03932933
H 5.79617297 0.64059242 0.04303820
H -1.12107752 -5.42537295 -2.15640752
H -1.10484781 -5.42346201 2.16594794
H -1.21015131 -6.70134229 0.00567520
H -2.34128362 -5.34219115 0.00934773
H 2.28649527 2.54415741 -0.00230622
H 1.06052348 -3.25197376 -0.00414541
H 4.42205361 3.06584673 1.32322003
H 4.44703967 3.05068656 -1.29124032
H 0.42855774 -5.36953576 -1.30825309
H 0.43833441 -5.36819029 1.30615495
H -3.34629682 0.70665048 -0.00624063
H -4.86315977 2.31913962 -1.30089643
H -4.87111477 2.29099611 1.31345592

6f
84

C 1.219416 -0.712679 4.100924
C 1.204735 0.686704 4.101674
C 0.000304 1.399374 4.101863
C -1.204217 0.687006 4.101019
C -1.219260 -0.712391 4.099733
C 0.000000 -1.399374 4.100000
H 2.150288 1.232494 4.101855
H -2.149627 1.233103 4.100943
H -0.000195 -2.491168 4.099138
C 0.000345 2.886011 4.101761
C 0.941485 3.602566 3.349312
H 1.665834 3.059576 2.739253
C 0.941193 4.995511 3.348030
H 1.676099 5.535677 2.749157
C 0.000000 5.697692 4.100000
H -0.000194 6.788612 4.099287
C -0.940929 4.996183 4.852962
H -1.675972 5.536880 5.451202
C -0.940908 3.603220 4.853435
H -1.665339 3.060748 5.463856
C -2.506875 -1.455292 4.096345
C -2.659332 -2.630677 4.845194
H -1.828721 -2.988597 5.456487
C -3.866038 -3.326472 4.841045
H -3.968372 -4.234664 5.437079
C -4.942507 -2.860094 4.087336
H -5.887481 -3.405172 4.083828
C -4.803067 -1.692246 3.338206
H -5.637015 -1.324086 2.738623
C -3.596510 -0.996321 3.343085
H -3.486945 -0.096257 2.735172
C 2.507024 -1.455806 4.099988
C 2.656681 -2.629801 3.348517
H 1.823718 -2.986763 2.739891
C 3.863520 -3.325419 3.346371
H 3.963513 -4.232481 2.748236
C 4.943021 -2.860121 4.096413
H 5.888205 -3.404865 4.094780
C 4.806317 -1.693874 4.848436
H 5.642715 -1.326567 5.445141
C 3.599560 -0.998120 4.849893
H 3.492403 -0.099104 5.459784
C 1.21941618 -0.71267896 0.00092364
C 1.20473451 0.68670402 0.00167410
C 0.00030431 1.39937427 0.00186358
C -1.20421686 0.68700597 0.00101949
C -1.21925984 -0.71239090 -0.00026650
C 0.00000000 -1.39937427 0.00000000
H 2.15028770 1.23249414 0.00185505
H -2.14962672 1.23310305 0.00094266
H -0.00019467 -2.49116754 -0.00086191
C 0.00034453 2.88601139 0.00176166
C 0.94148521 3.60256641 -0.75068769
H 1.66583431 3.05957626 -1.36074685
C 0.94119259 4.99551056 -0.75197036
H 1.67609870 5.53567720 -1.35084253
C 0.00000000 5.69769227 0.00000000
H -0.00019433 6.78861243 -0.00071272
C -0.94092936 4.99618286 0.75296207
H -1.67597173 5.53688069 1.35120225
C -0.94090807 3.60321944 0.75343480
H -1.66533945 3.06074762 1.36385565
C -2.50687526 -1.45529190 -0.00365518
C -2.65933218 -2.63067721 0.74519383
H -1.82872142 -2.98859662 1.35648709
C -3.86603755 -3.32647234 0.74104567
H -3.96837216 -4.23466427 1.33707941
C -4.94250750 -2.86009350 -0.01266432
H -5.88748126 -3.40517181 -0.01617172
C -4.80306657 -1.69224643 -0.76179349
H -5.63701470 -1.32408609 -1.36137740
C -3.59651008 -0.99632087 -0.75691483
H -3.48694478 -0.09625653 -1.36482797
C 2.50702388 -1.45580598 -0.00001162
C 2.65668089 -2.62980055 -0.75148323
H 1.82371803 -2.98676342 -1.36010897
C 3.86351996 -3.32541895 -0.75362892

H 3.96351284 -4.23248091 -1.35176422
C 4.94302142 -2.86012137 -0.00358686
H 5.88820468 -3.40486477 -0.00521995
C 4.80631736 -1.69387393 0.74843656
H 5.64271456 -1.32656670 1.34514065
C 3.59955966 -0.99812026 0.74989328
H 3.49240338 -0.09910413 1.35978405

6d

168

C -1.375731 -0.320685 4.708674
C -0.943051 1.017645 4.709097
C 0.407623 1.353635 4.702887
C 1.350263 0.309798 4.692146
C 0.966101 -1.027934 4.693036
C -0.409176 -1.322052 4.704551
C -2.873068 -0.620548 4.705765
H -1.693624 1.807522 4.713416
C 0.898429 2.799743 4.707768
H 2.409592 0.564888 4.683103
C 1.972859 -2.176132 4.684930
H -0.717863 -2.366909 4.708316
C -3.543258 -0.004764 5.957022
C -3.173007 -2.131633 4.705187
C -3.532157 -0.006534 3.447495
C 1.762784 3.071413 3.453767
C 1.764623 3.059639 5.963150
C -0.257954 3.817410 4.713502
C 3.431501 -1.684582 4.625000
C 1.729466 -3.087966 3.458711
C 1.812230 -3.028331 5.966189
H -3.083305 -0.437941 6.860773
C -5.056332 -0.260868 5.949665
H -3.350860 1.079612 5.987397
H -2.719656 -2.598893 5.595183
H -2.712228 -2.599504 3.819380
C -4.684673 -2.395836 4.698471
H -3.339271 1.077763 3.417587
C -5.045031 -0.262680 3.440993
H -3.063556 -0.440835 2.548733
H 2.603698 2.360552 3.419176
C 2.301589 4.508258 3.460613
H 1.153650 2.892370 2.552162
H 2.605305 2.348033 5.989348
H 1.156746 2.871764 6.863808
C 2.303196 4.496578 5.969362
H -0.892729 3.660822 3.825522
C 0.272970 5.257606 4.719737
H -0.890706 3.653094 5.601486
H 3.578386 -1.072471 3.719533
C 4.412852 -2.864769 4.617794
H 3.640192 -1.038865 5.494042
H 1.845162 -2.491077 2.538727
H 0.693436 -3.461795 3.470396
C 2.703570 -4.273545 3.454894
H 1.986549 -2.388276 6.847039
C 2.788028 -4.212958 5.961673
H 0.776961 -3.398706 6.038537
C -5.319483 -1.772096 5.948147
C -5.307925 -1.773924 3.442076
C -5.673857 0.366909 4.692064
H -5.504066 0.192827 6.847727
H -4.858512 -3.483192 4.698413
H -5.484415 0.189810 2.538200
H -6.404150 -1.968540 5.959903
H -4.894504 -2.229907 6.856382
H -6.392378 -1.970693 3.420368
H -4.874326 -2.232800 2.538456
H -6.764643 0.207701 4.687319
H -5.503904 1.456255 4.691980
C 3.159049 4.725467 4.715496
C 1.128299 5.482833 5.973319
C 1.126831 5.494635 3.467364
H 2.915609 4.668440 2.560530
H 2.918260 4.648319 6.870202
H -0.579951 5.954068 4.723545
H 3.569367 5.748602 4.719984
H 4.015502 4.031170 4.711775
H 1.502811 6.519425 5.994718
H 0.516904 5.337485 6.878963
H 1.501419 6.531355 3.455436
H 0.514376 5.357957 2.561098
C 4.228732 -3.689629 5.898138
C 4.143846 -3.750418 3.394107
C 2.511009 -5.096205 4.736753
H 5.441772 -2.474993 4.573574
H 2.500573 -4.905139 2.575847
H 2.646897 -4.801243 6.881901
H 4.939280 -4.532342 5.911003
H 4.443472 -3.067586 6.782490
H 4.853940 -4.593390 3.373862
H 4.296435 -3.172159 2.468146
H 3.191683 -5.963305 4.734637
H 1.482179 -5.490051 4.780895
C -0.9656044306 1.0304893778 0.0086735106
C 0.4097636173 1.3249426496 0.0090968285
C 1.3760761905 0.3232204886 0.0028867673
C 0.9434070043 -1.015047916 -0.0078540285
C -0.4071847035 -1.3512205123 -0.0069638033

C -1.3495363772 -0.3072548191 0.0045513442
C -1.973962587 2.1772902997 0.0057646982
H 0.7185298671 2.3698967776 0.0134162961
C 2.8738451607 0.621223203 0.0077679906
H 1.6939855073 -1.804909475 -0.0168970857
C -0.8981736434 -2.7971979079 -0.0150703948
H -2.4087524465 -0.5623520814 0.0083157851
C -1.7757728379 3.0655840306 1.2570223524
C -3.4325693225 1.6815024763 0.0051867729
C -1.7717546318 3.055085437 -1.2525049755
C 3.5412961719 0.0085049613 -1.2462328191
C 3.5320192458 0.0010260449 1.2631496622
C 3.1769798986 2.1315144685 0.0135020523
C 0.2568419267 -3.8146444087 -0.0749997157
C -1.8095414448 -3.0423298602 -1.2412894241
C -1.7165139932 -3.0841879778 1.2661893969
H -1.9209379801 2.4506648546 2.1607731476
C -2.7541022079 4.2478924877 1.2496651034
H -0.740476435 3.4411501832 1.2873972878
H -3.610552947 1.0552598521 0.8951834704
H -3.6073683754 1.048520778 -0.8806199821
C -4.4172086329 2.8585421265 -0.0015294657
H -0.73628307 3.4301893936 -1.2824127596
C -2.7500207631 4.2371992765 -1.2590072419
H -1.9135694688 2.4321142106 -2.151266532
H 3.3461295135 -1.0751784639 -1.2808238595
C 5.0550432952 0.2603096076 -1.2393872513
H 3.0816733548 0.4465098653 -2.1478375928
H 3.3360911886 -1.0828294045 1.2893478217
H 3.0653757395 0.4335254866 2.1638077457
C 5.0457306578 0.2530772513 1.2693620231
H 2.7239827695 2.602951877 -0.8744778784
C 4.6896877794 2.3918184169 0.0197374088
H 2.7183015094 2.597335365 0.9014859049
H 0.8603881981 -3.6357945469 -0.9804674376
C -0.2745546732 -5.2546119851 -0.0822055875
H 0.920394757 -3.6725169566 0.7940416718
H -1.234772097 -2.8440815714 -2.1612724811
H -2.6513023353 -2.3320159787 -1.2296041692
C -2.3492313369 -4.4787187846 -1.2451064543
H -1.0750510815 -2.915125863 2.1470391379
C -2.2545323436 -4.521567475 1.2616732808
H -2.5549033965 -2.3728064473 1.3385364812
C -4.194439351 3.7201739967 1.2481465177
C -4.190243697 3.7092499813 -1.2579236188
C -2.519193328 5.0965728054 -0.0079363327
H -2.5850577485 4.8624884878 2.1477274129
H -5.4458063446 2.4654133071 -0.001587144
H -2.5778450951 4.8439623749 -2.1618003553
H -4.906898478 4.5613010371 1.2599026316
H -4.3784253611 3.1232255853 2.1563820226
H -4.90287615 4.5500296994 -1.2796323262
H -4.370842394 3.1043037628 -2.1615436253
H -3.2024649909 5.9616174225 -0.0126814414
H -1.4908155327 5.4940630392 -0.0080202897
C 5.67188191 -0.3736686189 0.0154961956
C 5.3124050292 1.7636960749 1.2733187114
C 5.3218918697 1.7708674861 -1.2326361853
H 5.5007747502 -0.1913569677 -2.1394695837
H 5.48467521 -0.2037133694 2.1702016558
H 4.8663812324 3.4787010029 0.0235453396
H 6.763101384 -0.2174474375 0.0199842638
H 5.4988292234 -1.4625277962 0.0117751307
H 6.3973752295 1.9576545659 1.2947180772
H 4.880831931 2.2205045577 2.178963203
H 6.4070115521 1.9648251532 -1.2445642873
H 4.8972973144 2.2329308786 -2.1389023047
C -1.0809642002 -5.5075887129 1.1981379517
C -1.1760519216 -5.4644701657 -1.305893046
C -3.157955921 -4.7232861689 0.0367527882
H 0.5774615648 -5.9507949478 -0.1264258107
H -2.9977065298 -4.6187149287 -2.1241529334
H -2.8345673962 -4.6934867105 2.1819013689
H -1.4555010601 -6.5442979864 1.2110032554
H -0.434889275 -5.3825389693 2.0824901655
H -1.551040806 -6.5009160346 -1.3261380971
H -0.5989698784 -5.3074868353 -2.2318545168
H -3.5685498685 -5.7463174645 0.0346372536
H -4.0134516634 -4.0292159161 0.0808949508

10
96

C 4.747452 -1.456315 1.269880
C 4.327777 -0.717790 0.000000
C 4.872222 0.717790 0.000000
C 4.452548 1.456315 1.269880
C 4.871229 0.718819 2.540740
C 4.328771 -0.718819 2.540740
C 4.750392 -1.462183 3.809195
C 4.328785 -0.714543 5.073400
C 4.871215 0.714543 5.073400
C 4.449607 1.462183 3.809195
H 5.847490 -1.573047 1.269389
C 4.747452 -1.456315 -1.269880
C 4.452548 1.456315 -1.269880
H 3.352509 1.573047 1.269389
H 5.849621 -1.576674 3.801005
H 3.226486 -0.681644 5.121129
H 5.973514 0.681644 5.121129

H 3.350379 1.576674 3.801005
C 4.871229 0.718819 -2.540740
C 4.328771 -0.718819 -2.540740
C 4.449607 1.462183 -3.809195
C 4.871215 0.714543 -5.073400
C 4.328785 -0.714543 -5.073400
C 4.750392 -1.462183 -3.809195
H 4.324534 -2.474943 -1.269982
H 4.875465 2.474943 -1.269982
H 3.350379 1.576674 -3.801005
H 5.849621 -1.576674 -3.801005
H 4.324534 -2.474943 1.269982
H 3.222157 -0.643094 0.000000
H 5.977842 0.643094 0.000000
H 4.875465 2.474943 1.269982
H 5.977255 0.645125 2.542041
H 3.222745 -0.645125 2.542041
H 4.328843 -2.480087 3.802532
H 4.667311 -1.254935 5.970340
H 4.532689 1.254935 5.970340
H 4.871157 2.480087 3.802532
H 5.847490 -1.573047 -1.269389
H 3.352509 1.573047 -1.269389
H 5.977255 0.645125 -2.542041
H 3.222745 -0.645125 -2.542041
H 4.871157 2.480087 -3.802532
H 5.973514 0.681644 -5.121129
H 3.226486 -0.681644 -5.121129
H 4.328843 -2.480087 -3.802532
H 4.532689 1.254935 -5.970340
H 4.667311 -1.254935 -5.970340
C 0.1474520022 -1.4563147864 1.2698802159
C -0.2722225757 -0.7177896326 0.0000000012
C 0.2722225748 0.7177896337 0.0000000012
C -0.1474520027 1.4563147871 1.2698802164
C 0.2712288378 0.7188193386 2.54073987
C -0.2712288379 -0.7188193385 2.5407398699
C 0.1503923286 -1.4621827497 3.8091953574
C -0.2712147131 -0.7145434768 5.0733999299
C 0.2712147137 0.714543476 5.0733999301
C -0.1503923283 1.4621827495 3.8091953579
H 1.2474905851 -1.573046544 1.2693885358
C 0.1474519994 -1.4563147851 -1.269880216
C -0.1474519999 1.4563147857 -1.2698802165
H -1.2474905855 1.5730465446 1.2693885366
H 1.2496214953 -1.5766743401 3.8010052849
H -1.3735141189 -0.6816440103 5.1211290968
H 1.3735141195 0.6816440096 5.1211290967
H -1.2496214949 1.5766743399 3.8010052858
C 0.2712288393 0.7188193378 -2.5407398701
C -0.2712288395 -0.7188193377 -2.54073987
C -0.1503923253 1.4621827496 -3.809195358
C 0.2712147152 0.7145434755 -5.0733999303
C -0.2712147146 -0.7145434762 -5.0733999301
C 0.1503923256 -1.4621827498 -3.8091953575
H -0.2754655721 -2.4749430574 -1.2699823938
H 0.2754655716 2.474943058 -1.2699823947
H -1.2496214918 1.5766743421 -3.801005286
H 1.249621492 -1.5766743422 -3.8010052851
H -0.2754655682 -2.4749430591 1.2699823942
H -1.3778424194 -0.643093757 0.0000000012
H 1.3778424185 0.6430937581 0.0000000012
H 0.2754655677 2.4749430598 1.2699823951
H 1.3772551514 0.6451253904 2.542040964
H -1.3772551515 -0.6451253903 2.5420409643
H -0.2711574791 -2.4800867856 3.8025323364
H 0.0673108253 -1.2549350066 5.970339782
H -0.0673108245 1.2549350055 5.9703397825
H 0.2711574795 2.4800867853 3.8025323372
H 1.2474905821 -1.5730465434 -1.2693885376
H -1.2474905826 1.573046544 -1.2693885385
H 1.3772551528 0.6451253878 -2.5420409641
H -1.377255153 -0.6451253877 -2.5420409644
H 0.2711574844 2.4800867847 -3.802532337
H 1.3735141209 0.6816440069 -5.1211290968
H -1.3735141203 -0.6816440076 -5.1211290969
H -0.271157484 -2.4800867849 -3.8025323363
H -0.067310822 1.2549350057 -5.9703397826
H 0.0673108228 -1.2549350067 -5.9703397822

11
84

C 4.822270 -2.522844 0.729595
C 4.377730 -2.522844 -0.729595
C 4.849926 -1.269187 -1.464290
C 4.383078 -0.000000 -0.736224
C 4.816922 -0.000000 0.736224
C 4.350073 -1.269187 1.464290
C 4.402008 -1.257630 -2.925896
C 4.871258 0.000000 -3.653740
C 4.402008 1.257630 -2.925896
C 4.849926 1.269187 -1.464290
H 5.925314 -2.564244 0.776229
H 4.451273 -3.421300 1.248064
H 3.274686 -2.564244 -0.776229
H 4.748726 -3.421300 -1.248064
H 5.958064 -1.262333 -1.447253
H 3.272278 -0.000000 -0.753943
C 4.350073 1.269187 1.464290

C 4.797992 -1.257630 2.925896
H 3.298390 -1.306834 -2.955319
H 4.770881 -2.161909 -3.435785
H 4.511169 0.000000 -4.693602
H 5.973765 0.000000 -3.700280
H 3.298390 1.306834 -2.955319
H 4.770880 2.161909 -3.435785
C 4.377730 2.522844 -0.729595
H 5.927722 -0.000000 0.753943
H 3.241935 -1.262333 1.447253
H 5.958064 1.262333 -1.447253
H 3.241935 1.262333 1.447253
C 4.797992 1.257630 2.925896
H 5.901610 -1.306834 2.955319
H 4.429119 -2.161909 3.435785
C 4.328742 0.000000 3.653740
H 3.274686 2.564244 -0.776229
H 4.748726 3.421300 -1.248064
C 4.822270 2.522844 0.729595
H 4.429120 2.161909 3.435785
H 5.901610 1.306834 2.955319
H 4.688830 0.000000 4.693602
H 3.226234 0.000000 3.700280
H 4.451273 3.421300 1.248064
H 5.925314 2.564244 0.776229
C 0.2222699388 -2.5228444095 0.7295949859
C -0.2222699393 -2.5228444091 -0.7295949854
C 0.2499267608 -1.2691873852 -1.4642899922
C -0.2169220337 -0.0000000009 -0.7362241383
C 0.2169220364 -0.0000000009 0.7362241369
C -0.2499267602 -1.2691873845 1.4642899923
C -0.197992353 -1.2576299991 -2.9258961698
C 0.2712583822 0.0000000004 -3.653739682
C -0.197992355 1.2576299992 -2.9258961693
C 0.2499267591 1.2691873853 -1.4642899917
H 1.3253141261 -2.564244195 0.776228918
H -0.1487265967 -3.4213000507 1.2480636271
H -1.3253141267 -2.5642441931 -0.7762289169
H 0.1487265948 -3.4213000508 -1.2480636269
H 1.3580643622 -1.2623333272 -1.4472534087
H -1.3277221291 -0.0000000012 -0.7539430569
C -0.2499267585 1.2691873846 1.4642899917
C 0.197992353 -1.257629999 2.92589617
H -1.3016100676 -1.3068335006 -2.9553185869
H 0.1708805493 -2.1619089634 -3.4357850103
H -0.0888306223 0.0000000002 -4.6936019437
H 1.373765502 0.0000000011 -3.7002799624
H -1.3016100696 1.3068334996 -2.9553185862
H 0.1708805464 2.161908964 -3.4357850095
C -0.2222699405 2.5228444088 -0.7295949849
H 1.3277221318 -0.0000000012 0.7539430556
H -1.3580643615 -1.2623333251 1.4472534082
H 1.3580643605 1.2623333287 -1.4472534085
H -1.3580643598 1.2623333266 1.447253408
C 0.197992355 1.2576299991 2.9258961695
H 1.3016100676 -1.306833502 2.9553185873
H -0.1708805505 -2.1619089628 3.4357850105
C -0.2712583817 0.0000000004 3.6537396816
H -1.3253141279 2.5642441926 -0.7762289155
H 0.148726593 3.4213000506 -1.2480636268
C 0.2222699399 2.5228444092 0.7295949854
H -0.1708805476 2.1619089634 3.4357850097
H 1.3016100695 1.306833501 2.9553185866
H 0.088830622 0.0000000002 4.6936019436
H -1.3737655016 0.0000000011 3.7002799613
H -0.1487265949 3.4213000505 1.248063627
H 1.3253141273 2.5642441945 0.7762289166

9a

36

C 1.262394 0.728843 4.931868
C 1.262394 -0.728843 4.468132
C 0.000000 -1.457682 4.931857
C -1.262394 -0.728843 4.468132
C -1.262394 0.728843 4.931868
C 0.000000 1.457682 4.468143
H 2.161701 1.248082 4.567212
H 1.308161 0.755239 6.034808
H 2.161701 -1.248082 4.832788
H 1.308161 -0.755239 3.365191
H 0.000000 -1.510431 6.034791
H 0.000000 -2.496119 4.567181
H -2.161701 -1.248082 4.832788
H -1.308161 -0.755239 3.365191
H -1.308161 0.755239 6.034808
H -2.161701 1.248082 4.567212
H 0.000000 2.496119 4.832819
H 0.000000 1.510431 3.365209
C 1.2623943438 0.7288425811 0.2318676307
C 1.2623943438 -0.7288425811 -0.2318676307
C 0. -1.4576817735 0.2318568498
C -1.2623943438 -0.7288425811 -0.2318676307
C -1.2623943438 0.7288425811 0.2318676307
C 0. 1.4576817735 -0.2318568498
H 2.161701116 1.2480818419 -0.1327879713
H 1.308161501 0.7552393568 1.3348083094
H 2.161701116 -1.2480818419 0.1327879713
H 1.308161501 -0.7552393568 -1.3348083094

H 0. -1.5104306055 1.3347907901
H 0. -2.4961186944 -0.1328186408
H -2.161701116 -1.2480818419 0.1327879713
H -1.308161501 -0.7552393568 -1.3348083094
H -1.308161501 0.7552393568 1.3348083094
H -2.161701116 1.2480818419 -0.1327879713
H 0. 2.4961186944 0.1328186408
H 0. 1.5104306055 -1.3347907901

9b
54

C 0.723967 -1.253949 4.471862
C -0.735959 -1.274718 4.930291
C -1.447935 -0.000001 4.471862
C -0.736022 1.274722 4.930327
C 0.723964 1.253942 4.472080
C 1.471953 -0.000053 4.930327
H -1.498616 0.000059 3.366056
H -0.738482 1.278715 6.036049
H 0.749335 1.297885 3.366290
H 1.476641 -0.000187 6.036049
H 0.749359 -1.297810 3.366056
H -0.738140 -1.278496 6.036018
H -2.490072 -0.000015 4.832778
C -1.458429 2.526113 4.442863
H 1.245007 2.156415 4.833169
C 2.916892 0.000019 4.442863
H 1.245023 -2.156473 4.832778
C -1.458470 -2.526143 4.443109
H -0.958425 3.440164 4.795615
H -1.475107 2.555785 3.341791
H -2.500275 2.549780 4.794911
H 3.458312 -0.890412 4.794911
H 2.950928 0.000412 3.341791
H 3.458482 0.890061 4.795615
H -1.475574 -2.555770 3.342050
H -0.958195 -3.440151 4.795580
H -2.500161 -2.549896 4.795580
C -0.7239675881 -1.2539482548 0.1281382574
C -1.4719174321 0. -0.3302913315
C -0.7239675881 1.2539482548 0.1281382574
C 0.7359311772 1.2747751335 -0.3303268213
C 1.4479279244 0. 0.1279202445
C 0.7359311772 -1.2747751335 -0.3303268213
H -0.7492570736 1.2978694318 1.233943904
H 0.7381590651 1.2789018796 -1.4360486421
H 1.4986693932 0. 1.2337096409
H 0.7381590651 -1.2789018796 -1.4360486421
H -0.7492570736 -1.2978694318 1.233943904
H -1.4762800155 0. -1.4360179929
H -1.2450494153 2.1564580373 -0.2327780945
C 1.4584628632 2.5260927164 0.1571365796
H 2.4900144358 0. -0.2331686223
C 1.4584628632 -2.5260927164 0.1571365796
H -1.2450494153 -2.1564580373 -0.2327780945
C -2.9169392479 0. 0.1568912374
H 2.500056716 2.5501028332 -0.195615277
H 1.4758207308 2.5553725497 1.2582089695
H 0.958036843 3.4401919038 -0.1949107661
H 0.958036843 -3.4401919038 -0.1949107661
H 1.4758207308 -2.5553725497 1.2582089695
H 2.500056716 -2.5501028332 -0.195615277
H -2.9511491972 0. 1.2579504315
H -3.4583562486 -0.8902553606 -0.1955799135
H -3.4583562486 0.8902553607 -0.1955799135

9c
108

C 0.724582 -1.266840 5.739457
C -0.732608 -1.268914 6.219341
C -1.459408 -0.005913 5.739457
C -0.730549 1.277704 6.155184
C 0.707507 1.225439 5.629188
C 1.471798 0.006178 6.155184
H -1.550251 -0.029114 4.638666
H -0.680127 1.271746 7.261576
H 0.674149 1.167662 4.527795
H 1.441427 0.046866 7.261576
H 0.749911 -1.357113 4.638666
H -0.699154 -1.210970 7.325223
H -2.477705 0.000935 6.147668
C -1.480300 2.588536 5.773145
H 1.242069 2.151327 5.884064
C 2.981887 0.012291 5.773145
H 1.239661 -2.145287 6.147668
C -1.494723 -2.588935 5.896682
C -0.852962 3.772281 6.522842
C -1.415468 2.865479 4.265861
C -2.953328 2.500638 6.195555
C 3.642280 -1.307338 6.195555
C 3.189311 0.206908 4.265861
C 3.693371 1.147454 6.522842
C -1.631323 -2.825533 4.387286
C -0.752383 -3.782096 6.516005
C -2.899201 -2.542630 6.516005
H 0.204579 3.916316 6.260566
H -1.383098 4.704822 6.276143
H -0.918128 3.624601 7.612067

H -0.389198 3.083042 3.936150
H -1.790881 2.012452 3.680103
H -2.036377 3.739451 4.015681
H -3.045791 2.181978 7.246238
H -3.434469 3.485518 6.097474
H -3.518812 1.793478 5.572344
H 3.312602 -2.150641 5.572344
H 4.735781 -1.231578 6.097474
H 3.412543 -1.546743 7.246238
H 2.638274 -0.544722 3.680103
H 2.864591 1.204466 3.936150
H 4.256647 0.106172 4.015681
H 3.598060 1.017179 7.612067
H 4.766044 1.154613 6.276143
H 3.289339 2.135329 6.260566
H -0.650243 -2.909005 3.897057
H -2.173978 -3.765440 4.202250
H -2.194151 -2.017629 3.897057
H 0.211065 -3.971608 6.022655
H -0.562225 -3.615348 7.588240
H -1.355959 -4.697055 6.418129
H -3.545046 -1.803016 6.022655
H -3.389790 -3.522822 6.418129
H -2.849872 -2.294574 7.588240
C -0.7348251397 -1.2609273319 0.0605425454
C -1.46521576 0.0000000016 -0.4193408211
C -0.7348251367 1.2609273332 0.0605425461
C 0.7412486808 1.2715260419 -0.35518408
C 1.4150152173 -0.0000000019 0.1708122584
C 0.741248678 -1.2715260439 -0.3551840805
H -0.8003392752 1.3279992819 1.1613342964
H 0.7613005358 1.224880085 -1.4615756757
H 1.3482994771 -0.0000000022 1.2722045961
H 0.7613005336 -1.2248800864 -1.4615756763
H -0.8003392785 -1.3279992808 1.1613342956
H -1.398307726 0.0000000025 -1.5252225612
H -1.2380429733 2.1462222666 -0.3476677008
C 1.5015883962 2.5762454748 0.0268549734
H 2.4841384088 -0.0000000035 -0.0840635496
C 1.501588392 -2.5762454776 0.0268549728
H -1.2380429791 -2.1462222636 -0.3476677019
C -2.9894454133 0.0000000018 -0.0966823017
C 2.8404101897 2.6248266789 -0.722842082
C 1.7738432258 2.658570057 1.5341390826
C 0.6889515178 3.8079756011 -0.3955552619
C 0.6889515217 -3.8079756043 -0.3955552771
C 1.7738432077 -2.6585700671 1.5341390841
C 2.840410193 -2.6248266728 -0.7228420696
C -3.2626446732 0.0000000083 1.4127144563
C -3.6515832441 -1.2394656944 -0.7160047967
C -3.6515832457 1.2394656928 -0.7160048063
H 3.4939176133 1.7809874845 -0.4605658641
H 3.3829457525 3.5502093518 -0.4761433578
H 2.6799322954 2.6074218906 -1.8120667671
H 2.4753929512 1.8785764785 1.8638504413
H 0.8473939878 2.5571740557 2.1198970228
H 2.220271382 3.6332786715 1.7843188134
H 0.3667534684 3.7287205756 -1.4462383149
H 1.3013118037 4.7170957625 -0.297474298
H -0.2062087757 3.944118555 0.227655894
H -0.2062087767 -3.9441185653 0.2276558701
H 1.3013118101 -4.717095764 -0.2974743132
H 0.3667534814 -3.7287205737 -1.4462383325
H 0.8473939638 -2.5571740713 2.1198970161
H 2.4753929277 -1.8785764884 1.8638504536
H 2.2202713641 -3.6332786817 1.784318814
H 2.6799323094 -2.6074218814 -1.8120667562
H 3.3829457579 -3.550209344 -0.4761433433
H 3.4939176098 -1.7809874761 -0.4605658422
H -2.844393888 -0.8913756608 1.9029430591
H -4.3479555408 0.0000000122 1.5977501458
H -2.8443938863 0.8913756815 1.9029430502
H -3.3339808248 -2.1685917044 -0.2226551761
H -3.4120955919 -1.3207736674 -1.7882398048
H -4.745749051 -1.1742331844 -0.6181290291
H -3.3339808311 2.1685917066 -0.2226551899
H -4.7457490527 1.17423318 -0.6181290421
H -3.4120955901 1.3207736603 -1.7882398141

9e

132

C -4.134050 3.638345 5.037933
C -3.969531 3.393892 6.537809
C -2.636790 2.709248 6.843205
C -2.481638 1.395645 6.066827
C -2.634559 1.664323 4.564050
C -3.970902 2.339312 4.249300
C -1.200555 0.632359 6.449929
C 0.096336 1.309977 5.993832
C 1.335801 0.558650 6.492527
C 1.284891 -0.894864 6.009246
C 0.003660 -1.602973 6.463731
C -1.218006 -0.822333 5.966756
C 2.646991 1.291208 6.152860
C 3.840340 0.757717 6.954875
C 5.109473 1.569170 6.691466
C 5.442444 1.605384 5.200142
C 4.258400 2.126690 4.386437
C 2.995582 1.306823 4.658924

C -0.006248 -3.096961 6.091060
C -1.080606 -3.885705 6.850091
C -0.998026 -5.383824 6.554526
C -1.095225 -5.657761 5.053832
C -0.036934 -4.869555 4.282661
C -0.130737 -3.373257 4.587178
H 1.272327 0.535672 7.597262
H 0.133516 2.353630 6.346544
H 0.114154 1.343357 4.890556
H -1.178562 0.601943 7.556111
H -2.150221 -1.308087 6.298591
H -1.225267 -0.834730 4.863026
H -0.011072 -1.559862 7.569582
H 2.161950 -1.455263 6.372142
H 1.330684 -0.906621 4.906442
H 3.598228 0.762687 8.029827
H 4.026064 -0.294573 6.676863
H 2.153615 1.701912 4.069437
H 3.165715 0.271008 4.316603
H -2.543558 2.516005 7.924036
H -1.813983 3.393643 6.571637
H -2.540260 0.728218 3.992027
H -1.816121 2.325792 4.229798
H -0.982005 -3.702293 7.932151
H -2.079602 -3.518401 6.556317
H 0.642508 -2.824512 4.027406
H -1.106889 -3.000302 4.230847
H 5.953586 1.155793 7.264108
H 4.495821 2.114298 3.311820
H 5.689181 0.583854 4.861419
H 6.336049 2.222004 5.019495
H -1.788703 -5.924799 7.096294
H -0.139694 -5.045415 3.201069
H -2.096464 -5.358424 4.697422
H -0.997491 -6.735231 4.851657
H -4.049302 4.340917 7.092891
H -4.054869 2.532818 3.169094
H -3.367428 4.359612 4.704507
H -5.111895 4.097400 4.827116
H -3.329026 0.748506 6.365906
H -4.794182 2.750374 6.890813
H -4.793393 1.651283 4.511757
H 2.502937 2.344992 6.461200
H 4.958362 2.601506 7.052313
H 4.070171 3.180545 4.656107
H 0.972150 -3.503390 6.413260
H -0.035295 -5.772134 6.930178
H 0.965205 -5.234272 4.568097
C 1.2325890312 5.3827346355 1.5620669502
C 1.103146065 5.1180298638 0.0621906975
C 1.1765976016 3.6215205222 -0.2432048802
C 0.1165603503 2.8303537813 0.5331729396
C 0.2727814948 3.0971258312 2.0359498648
C 0.189167723 4.5919277505 2.3506997379
C 0.0960771549 1.339259854 0.1500706888
C 1.3313566553 0.5549280791 0.6061678918
C 1.3004211373 -0.8941434074 0.1074729607
C 0.0161853252 -1.57681101 0.5907538483
C -1.2376698009 -0.8212865285 0.1362690331
C -1.172449207 0.6270269759 0.6332436415
C 2.5904296412 -1.6633886508 0.4471403709
C 2.7250872025 -2.9636047269 -0.3548748012
C 4.0623932607 -3.6569790752 -0.0914661729
C 4.2602398662 -3.9272333005 1.3998581429
C 4.1196824057 -2.6411680793 2.2135628782
C 2.7782477413 -1.9574693839 1.9410763757
C -2.5364560775 -1.5597005042 0.5089403929
C -3.7567066641 -1.0236503489 -0.250091048
C -5.0128271186 -1.8442273171 0.0454740151
C -5.2986617565 -1.8970181445 1.5461676342
C -4.0869102356 -2.4194222813 2.3173393022
C -2.8379801049 -1.590037423 2.0128223871
H 1.2487844541 -0.8506620125 -0.9972618886
H 2.2537765079 1.0445560298 0.2534558963
H 1.3691731801 0.5561876407 1.7094443396
H 0.0807325861 1.3050055487 -0.9561107119
H -2.0592316737 1.1914723608 0.3014091629
H -1.1868153169 0.627116765 1.7369744511
H -1.2077010146 -0.7869724019 -0.969581674
H -0.0306048489 -2.6165654268 0.2278579366
H 0.0289004897 -1.6223466768 1.693558456
H 2.6083351957 -2.7514441516 -1.4298274488
H 1.9066398168 -3.6505912236 -0.0768625964
H 2.699421743 -1.0307601689 2.5305633636
H 1.9662722616 -2.6227158561 2.2833966792
H 1.0558602155 3.4441575763 -1.3240360619
H 2.1807045374 3.2511466295 0.0283627205
H -0.4907591455 2.5474083463 2.6079728068
H 1.2548491061 2.7190725598 2.3702014972
H -3.5485674049 -1.0173355114 -1.332151131
H -3.9381109698 0.0251570533 0.0436827296
H -1.9761302236 -1.9853145843 2.5725937103
H -3.0030669435 -0.5581876399 2.3691532875
H 4.1264538547 -4.5946907072 -0.6641082608
H 4.2276614261 -2.8529762865 3.2881795712
H 3.4989382171 -4.651679526 1.7385814588
H 5.2410516141 -4.3928077653 1.580504671
H -5.8766626908 -1.4299680394 -0.4962941111
H -4.2905891675 -2.4183593436 3.3989307569
H -5.540048304 -0.8802517369 1.902577803

H -6.1829112153 -2.5203935837 1.7483431841
H 1.8834083782 5.6606264828 -0.4928905735
H 0.3147651859 4.7613979573 3.4309056737
H 2.2405364306 5.0794541136 1.8954934064
H 1.1412200437 6.4591002687 1.7728842581
H -0.8675727799 3.2406429449 0.2340937443
H 0.1335178246 5.5104400981 -0.2908130167
H -0.8179284414 4.9602110893 2.0882426548
H 3.4310064534 -1.0117416482 0.1387999006
H 4.8808663368 -3.0099451966 -0.4523129658
H 4.9382338726 -1.9512299254 1.9438931397
H -2.3992348328 -2.6102323709 0.1867402614
H -4.8677468679 -2.8721314305 -0.3301775916
H -3.9016945814 -3.4696583625 2.0319026597

9f
96

C -1.260631 -0.728293 5.628006
C -1.272396 0.733876 6.094057
C -0.000106 1.454121 5.626973
C 1.269813 0.733818 6.100000
C 1.259661 -0.728408 5.633849
C -0.001589 -1.468314 6.100000
H -2.161001 -1.246896 5.991347
H -1.304730 -0.756880 4.525015
H -1.262141 0.728545 7.198360
H 0.002459 1.505263 4.523934
H -0.000982 2.493581 5.989124
H 1.254538 0.728623 7.204240
H 1.308883 -0.756508 4.531106
H 2.158284 -1.247253 6.001174
H -0.004108 -1.453462 7.204251
C -0.002261 -2.919467 5.668151
C 0.004717 -3.265975 4.310417
H 0.012323 -2.480683 3.550530
C 0.001811 -4.600510 3.911759
H 0.007250 -4.848688 2.849119
C -0.008205 -5.617814 4.866541
H -0.010704 -6.663403 4.555178
C -0.015154 -5.287786 6.219865
H -0.023092 -6.075131 6.975417
C -0.012182 -3.949442 6.613722
H -0.017883 -3.697093 7.676753
C 2.528286 1.457440 5.670023
C 3.418740 1.971354 6.617722
H 3.196855 1.846466 7.680227
C 4.580407 2.637432 6.226667
H 5.261144 3.030307 6.983692
C 4.870465 2.800422 4.873926
H 5.778212 3.320651 4.564769
C 3.990991 2.292984 3.917015
H 4.209383 2.415577 2.854881
C 2.832519 1.628761 4.312903
H 2.153695 1.236710 3.551525
C -2.527892 1.459307 5.658525
C -2.825124 1.632545 4.300106
H -2.143599 1.239235 3.541765
C -3.979625 2.300698 3.899192
H -4.192362 2.425035 2.836115
C -4.862141 2.810099 4.852262
H -5.766542 3.333743 4.539113
C -4.579196 2.644902 6.206230
H -5.262339 3.039257 6.960325
C -3.421454 1.974960 6.602323
H -3.205020 1.848722 7.665789
C -1.26004565 0.72649043 0.47199372
C 0.00034715 1.46776407 0.00594346
C 1.26024321 0.72605149 0.47302688
C 1.27140182 -0.73388204 0.00000000
C 0.00000122 -1.45620390 0.46615075
C -1.27140182 -0.73388204 0.00000000
H -2.15935440 1.24693288 0.10865262
H -1.30685219 0.75038823 1.57498477
H 0.00085818 1.45621694 -1.09835959
H 1.30581522 0.74940074 1.57606650
H 2.16000376 1.24654002 0.11087579
H 1.25926548 -0.72325132 -1.10424020
H 0.00027616 -1.51288097 1.56889393
H -0.00002105 -2.49385660 0.09882557
H -1.25979873 -0.72427458 -1.10425130
C -2.52847300 -1.45887689 0.43184905
C -2.82506928 -1.63817402 1.78958311
H -2.14118304 -1.25211491 2.54947033
C -3.98226281 -2.30292426 2.18824048
H -4.19447131 -2.43172396 3.25088110
C -4.86828186 -2.80290290 1.23345874
H -5.77503810 -3.32353341 1.54482234
C -4.58594361 -2.63187048 -0.11986549
H -5.27177384 -3.01866922 -0.87541720
C -3.42541755 -1.96527234 -0.51372189
H -3.20972853 -1.83416069 -1.57675260
C 2.52731354 -1.46194093 0.42997656
C 3.41760281 -1.97614031 -0.51772221
H 3.19850462 -1.84642584 -1.58022721
C 4.57527643 -2.64913440 -0.12666698
H 5.25588470 -3.04223247 -0.88369201
C 4.86145991 -2.81883675 1.22607360
H 5.76586388 -3.34485449 1.53523050
C 3.98226834 -2.31090894 2.18298452

H 4.19763272 -2.43874555 3.24511928
C 2.82779761 -1.63975384 1.78709661
H 2.14885984 -1.24790082 2.54847520
C 0.00084099 2.91777057 0.44147514
C 0.00225345 3.26180088 1.79989385
H 0.00239952 2.47492713 2.55823499
C 0.00364030 4.59570347 2.20080769
H 0.00495093 4.84210759 3.26388531
C 0.00353642 5.61468610 1.24773788
H 0.00482529 6.65974258 1.56088711
C 0.00194402 5.28704985 -0.10623037
H 0.00189441 6.07584638 -0.86032476
C 0.00062863 3.94944507 -0.50232277
H -0.00047982 3.69888889 -1.56578882

9d

180

C -0.740047 1.260138 5.659275
C -1.472943 -0.023006 5.247795
C -0.744142 -1.288896 5.722306
C 0.716551 -1.287109 5.247796
C 1.461337 -0.010830 5.659274
C 0.693406 1.201012 5.121379
C -1.480747 2.576572 5.291299
H -0.680834 1.251219 6.765372
H -2.484513 -0.015377 5.669349
H -1.577477 -0.042782 4.147946
H -0.711663 -1.232639 6.828696
C -1.505279 -2.607221 5.401446
H 1.228943 -2.159339 5.669351
H 0.751692 -1.387530 4.147947
H 1.424008 0.035990 6.765371
C 2.971752 0.005922 5.291297
H 1.230718 2.131666 5.351572
H 0.647711 1.121868 4.022322
C -1.403849 2.907095 3.786361
C -2.971595 2.501924 5.685016
C -0.854637 3.754635 6.071050
C -1.655127 -2.866780 3.887946
C -0.764198 -3.814803 6.018093
C -2.921621 -2.569208 6.018079
C 3.219542 0.237780 3.786358
C 3.678930 1.137181 6.071047
C 3.652530 -1.322513 5.685013
H -1.843173 2.083861 3.197120
C -2.135982 4.222513 3.475868
H -0.350961 3.002668 3.476523
H -3.469069 1.699560 5.118925
H -3.055151 2.245093 6.756136
C -3.697402 3.822192 5.392122
H 0.212630 3.852765 5.817391
C -1.568922 5.077142 5.762606
H -0.911296 3.541309 7.152430
H -0.661214 -2.919861 3.413987
C -2.413230 -4.179851 3.630691
H -2.198045 -2.032567 3.413978
H 0.241258 -3.904658 5.579907
H -0.633296 -3.646535 7.101655
C -1.519144 -5.127448 5.770879
H -3.502158 -1.743384 5.579884
C -3.680935 -3.879331 5.770866
H -2.841356 -2.371705 7.101642
H 2.775866 1.197393 3.476521
C 4.724795 0.261445 3.475864
H 2.726263 -0.554303 3.197118
H 3.230278 2.110527 5.817389
H 3.522516 0.981450 7.152428
C 5.181396 1.179847 5.762602
H 3.206400 -2.154521 5.118922
C 5.158819 -1.290946 5.392118
H 3.471888 -1.523290 6.756133
C -1.476043 5.366439 4.258165
C -3.041783 4.966467 6.173805
C -3.609748 4.111285 3.887428
H -2.068162 4.426916 2.395781
H -4.753570 3.727067 5.689521
H -1.085797 5.890536 6.326475
H -0.420033 5.468254 3.958088
H -1.974702 6.321544 4.024683
H -3.117479 4.776350 7.257048
H -3.566882 5.914379 5.970788
H -4.101010 3.304082 3.319466
H -4.140404 5.047660 3.648656
C 5.821980 -0.151026 6.173799
C 5.385493 1.404931 4.258161
C 5.365351 -1.070489 3.887424
H 4.867901 0.422380 2.395777
H 5.644255 2.004942 6.326470
H 5.604523 -2.253177 5.689516
H 5.695185 -0.311638 7.257042
H 6.905446 -0.131819 5.970782
H 4.945662 2.370369 3.958083
H 6.461968 1.450634 4.024677
H 4.911925 -1.899536 3.319462
H 6.441604 -1.061862 3.648650
C -1.645518 -5.351438 4.257613
C -3.811716 -4.100777 4.257599
C -2.915266 -5.049369 6.398074

H -2.503760 -4.336663 2.544332
H -0.954990 -5.958922 6.221853
H -4.683095 -3.806489 6.221830
H -0.643922 -5.432798 3.804084
H -2.172283 -6.298868 4.056833
H -4.382967 -3.274049 3.804061
H -4.368834 -5.030681 4.056819
H -2.832197 -4.905478 7.487922
H -3.460343 -5.993465 6.233529
C 0.7212877745 1.2709678128 -0.5592749317
C -0.7563949098 1.2641032005 -0.1477954179
C -1.4882878602 -0.0000015532 -0.6223059335
C -0.7563943079 -1.2641064077 -0.1477962333
C 0.7212888529 -1.2709698685 -0.5592744504
C 1.3868102097 -0.0000006132 -0.0213792175
C 1.491003003 2.5706507437 -0.1912987254
H 0.7431699444 1.2152287822 -1.665371771
H -1.2555730536 2.143963371 -0.5693492773
H -0.8257892403 1.3447440752 0.9520535288
H -1.4233278151 -0.000000986 -1.7286964283
C -3.0105586339 -0.000001065 -0.3014460396
H -1.2555712836 -2.1439664926 -0.5693514921
H -0.8257895921 -1.344748568 0.9520525742
H 0.7431718482 -1.2152316368 -1.6653713123
C 1.4910048004 -2.5706519135 -0.1912966806
H 2.4614359653 -0.0000001583 -0.2515721655
H 1.2954222956 -0.0000004422 1.0776784941
C 1.8156943249 2.6693158326 1.3136393602
C 0.680932248 3.8244387537 -0.5850160383
C 2.8242905367 2.6174552667 -0.9710502426
C -3.3102682448 -0.0000082602 1.2120542015
C -3.6858150327 -1.2455871634 -0.9180925365
C -3.6858097341 1.2455942793 -0.9180794895
C 1.8156954285 -2.6693152265 1.3136416669
C 2.8242927507 -2.6174563819 -0.9710474972
C 0.680935046 -3.8244408437 -0.5850130923
H 0.8830901574 2.638164849 1.9028802035
C 2.5888131486 3.9610714683 1.6241317011
H 2.4249059807 1.8052749433 1.6234771464
H -0.2626722848 3.8540816491 -0.0189248809
H 0.4167323501 3.7683845235 -1.656135534
C 1.4614135193 5.1131400332 -0.2921224572
H 3.4429073048 1.742239559 -0.7173907636
C 3.6124734445 3.8972969271 -0.6626064483
H 2.6112159892 2.5598601919 -2.0524302409
H -2.8592805255 -0.8873031302 1.6860125984
C -4.8264724541 -0.0000066044 1.4693088607
H -2.8592771654 0.8872800319 1.6860216832
H -3.260903686 -2.1612653423 -0.4799065599
H -3.4746397501 -1.2748165785 -2.0016552063
C -5.2000720433 -1.2481066528 -0.670879102
H -3.2608939017 2.1612660761 -0.4798843993
C -5.2000666608 1.2481179556 -0.6708656727
H -3.4746346968 1.2748337377 -2.0016419245
H 2.4249063143 -1.8052735799 1.6234788736
C 2.5888149654 -3.9610700099 1.6241357538
H 0.8830909371 -2.638164287 1.9028819991
H 3.4429088467 -1.7422400306 -0.7173886151
H 2.6112187072 -2.559862563 -2.0524276625
C 3.6124763267 -3.8972972163 -0.6626019685
H -0.2626697664 -3.8540837477 -0.0189223971
C 1.4614170029 -5.1131413134 -0.2921177417
H 0.4167356711 -3.7683879031 -1.6561327831
C 3.9094512151 3.9615098023 0.8418347677
C 2.7801946028 5.1174947422 -1.0738045818
C 1.7556025438 5.1817756684 1.2125715381
H 2.7997413431 4.0045389934 2.7042186877
H 0.8509487705 5.9802461853 -0.5895209547
H 4.5584549964 3.8855964491 -1.2264748714
H 4.5256297469 3.0978861897 1.1419124305
H 4.4872668887 4.8709142622 1.0753168641
H 2.5777011395 5.0879910798 -2.1570475254
H 3.3385609896 6.0462001168 -0.8707884786
H 0.8109138373 5.2036201408 1.7805339831
H 2.3011997128 6.1095249274 1.4513437638
C 2.7801984734 -5.1174960066 -1.0737992107
C 3.9094534089 -3.9615083024 0.8418394621
C 1.7556053436 -5.181775175 1.2125764721
H 2.7997426579 -4.0045362485 2.7042228899
H 4.5584581467 -3.8855967246 -1.2264699398
H 0.8509529569 -5.9802481738 -0.5895156151
H 2.5777055284 -5.0879936332 -2.1570422857
H 3.3385653568 -6.0462008056 -0.8707818377
H 4.5256312222 -3.0978839614 1.1419165045
H 4.487269569 -4.8709121322 1.0753228091
H 0.8109163733 -5.2036196443 1.7805384774
H 2.3012029879 -6.1095238327 1.4513499519
C -5.4572401535 -1.2506577893 0.8423874328
C -5.4572347893 1.2506539891 0.8424008995
C -5.8305149411 0.0000104019 -1.2980740877
H -5.0075402813 -0.0000120423 2.555668142
H -5.6380731236 -2.1524162338 -1.1218531022
H -5.6380637885 2.1524342767 -1.1218299986
H -5.0269021499 -2.1587460507 1.2959161322
H -6.541120658 -1.2681819951 1.0431669699
H -5.0268928976 2.1587355392 1.2959393435
H -6.5411152174 1.2681806637 1.043180632
H -5.6643673645 0.0000159077 -2.3879219141
H -6.9206654984 0.0000118923 -1.1335290654

Optimized dimers (M06-2X/def2-SVP)

7
60
0 1
C 3.9122207368 0.9728276089 -2.927382749
C 3.6644306461 -0.4514270319 -2.9495061073
C 3.6422354932 -1.1255562059 -4.2187416548
C 3.8397052225 -0.44144923 -5.3798806165
C 4.0725147447 0.9741528656 -5.3587460363
C 4.1092793444 1.653447467 -4.1784196309
C 3.9464614277 1.6411490042 -1.710212804
C 3.452790528 -1.1253055375 -1.7532836754
H 3.4539192165 -2.2011384227 -4.2285853226
H 3.8200532065 -0.96558682 -6.3366231517
H 4.2266173262 1.5039737326 -6.3002096008
H 4.2924857743 2.729950217 -4.1595932263
C 3.4852761645 -0.4566606598 -0.511292438
C 3.7445277694 0.9641156407 -0.4888018152
H 4.137778176 2.7172278941 -1.693806135
H 3.2507092334 -2.1993607499 -1.7710159942
C 3.2695716388 -1.1314969789 0.709032467
C 3.7806402204 1.6314477447 0.7535938255
C 3.5749757266 0.9554765365 1.9496180003
C 3.3095756179 -0.4655212975 1.927418165
H 3.0597227781 -2.2040694048 0.6904368135
H 3.9798014986 2.7060615376 0.7720583035
C 3.6101848969 1.625671202 3.2214967149
C 3.0878414123 -1.1436326492 3.1760745428
C 3.4001736438 0.9401998897 4.3794971311
C 3.1332135067 -0.4691935212 4.3581295208
H 3.8123767291 2.6988748688 3.2365006396
H 2.8721698282 -2.2140558961 3.1521670162
H 3.4258553165 1.4619976774 5.33762405
H 2.9523930687 -0.9942606462 5.2971658619
C 0.3907163761 0.465547683 -1.927368327
C 0.1251180214 -0.9554065223 -1.9496321423
C 0.0896917816 -1.6254912206 -3.2215618208
C 0.2996458486 -0.9399540737 -4.3795365915
C 0.5668078095 0.4693953893 -4.3581055492
C 0.6124510769 1.1437180304 -3.1759949491
C 0.4308099452 1.1314644565 -0.7089513728
C -0.0805508443 -1.6314305956 -0.7536336113
H -0.112370805 -2.6987180921 -3.2366639193
H 0.2737303959 -1.4617010381 -5.3376859174
H 0.7475926047 0.9945122794 -5.297120319
H 0.8283049552 2.2141024518 -3.1520126713
C -0.0443535184 -0.9641557292 0.488789417
C 0.2150041239 0.4565988796 0.5113362802
H 0.6407818073 2.2040103028 -0.6903047296
H -0.2798354241 -2.7060205076 -0.7721484901
C -0.2463827136 -1.6412148165 1.7101753591
C 0.2474574052 1.1252087017 1.7533489308
C 0.0356287115 0.4513235219 2.9495297069
C -0.2122455565 -0.9729108962 2.9273565386
H -0.4377160152 -2.7172905118 1.693731331
H 0.4496718801 2.1992375197 1.7711298539
C 0.057648897 1.1254299876 4.2187829675
C -0.4095194814 -1.6535408975 4.1783567655
C -0.140071174 0.441331498 5.379881779
C -0.3729300062 -0.9742638268 5.3586984767
H 0.2459964427 2.2010048813 4.2286542002
H -0.5927725065 -2.7300347141 4.1594811461
H -0.1205741924 0.9654709782 6.3366262781
H -0.5272367377 -1.5040819547 6.3001292859

8
52
0 1
C 3.2009172099 -0.8899989836 2.1286080991
C 3.1541589925 -1.7587434043 1.0872049052
C 3.2332395989 -1.2979455734 -0.2768833631
C 3.359413034 0.098410534 -0.5211930755
C 3.4043168913 1.0144884182 0.5750674072
C 3.3244839857 0.5299699577 1.9104335306
C 3.1926348873 -2.1781445226 -1.3667868877
C 3.2736927275 -1.69918052 -2.6721688105
C 3.3924230427 -0.3333082956 -2.9171855208
C 3.4373255919 0.5820880827 -1.8569611812
H 3.1319425352 -1.2516163021 3.1564416898
H 3.047442023 -2.8310479758 1.2646878112
C 3.5269749008 2.4116306105 0.331337942
C 3.3524829582 1.4475622562 2.9694466133
H 3.0911004511 -3.2489033909 -1.1783701172
H 3.2408145754 -2.3989326967 -3.508852242
H 3.4491729391 0.0396446194 -3.9420492849
C 3.5634175621 2.0019781152 -2.0729109118
C 3.5584900769 3.2940850519 1.4200013566
H 3.2826021626 1.0754248333 3.9939520935
C 3.4677721214 2.8143007793 2.7243242982
H 3.6201321704 2.3635678998 -3.1019472936
C 3.6076223967 2.8723527261 -1.0326305399
H 3.6536681855 4.3657160717 1.2328104609
H 3.4916417502 3.5141284414 3.561288568
H 3.7043861058 3.9455579975 -1.2105306894
C -0.2644987933 -2.7203384344 -0.2572994801
C -0.220263747 -2.1586683944 -1.4918945479
C -0.0952601134 -0.7324966021 -1.6621194654
C -0.0188612091 0.0897825754 -0.5035603692
C -0.0636146065 -0.5009713623 0.7971574548

C -0.1848209732 -1.9127167788 0.9345624555
C -0.0497857022 -0.1328993704 -2.9279904405
C 0.067651926 1.2489404092 -3.055908798
C 0.1474298886 2.0586664174 -1.9253804549
C 0.106281827 1.5009024004 -0.6402812549
H -0.36040486 -3.802219312 -0.1427333351
H -0.2758730601 -2.7814706642 -2.3873795712
C 0.0156955394 0.3219182291 1.955135169
C -0.2148710968 -2.4732385354 2.2189968912
H -0.1050956013 -0.7657136242 -3.8163237986
H 0.1009707631 1.7003308863 -4.0488396258
H 0.2483760736 3.1409625621 -2.0292818645
C 0.1844574134 2.308690177 0.5516858632
C -0.0106579216 -0.2803592599 3.2203553993
H -0.308631267 -3.5561186653 2.3242176311
C -0.1240932571 -1.6632273996 3.3482687063
H 0.2905214446 3.3895805142 0.436972026
C 0.1381046932 1.7488280997 1.7869449287
H 0.0587976116 0.3512592394 4.1087279056
H -0.1465272535 -2.1147855672 4.3414666407
H 0.2064634051 2.3713477301 2.6812270767

8 stuck

52
0 1
C 3.6096419608 -2.4641256849 0.6783255185
C 3.6089215156 -2.4641224525 -0.6783266196
C 3.6095121375 -1.2318787576 -1.4269806144
C 3.6067797869 0.0000057148 -0.7147074461
C 3.6071349922 0.000004956 0.7147089626
C 3.6104454151 -1.2318812142 1.4269802503
C 3.61735364 -1.2090379983 -2.8281240433
C 3.6221378146 -0.0000091165 -3.5190044645
C 3.6190018718 1.2090315911 -2.8281299651
C 3.6113237679 1.2318854142 -1.4269837807
H 3.6067442033 -3.4048499399 1.2330148561
H 3.6054070154 -3.4048468005 -1.2330148292
C 3.611606853 1.231886255 1.4269843929
C 3.6183988043 -1.2090391883 2.828124505
H 3.617293707 -2.1547732944 -3.3741008096
H 3.62519276 -0.0000121489 -4.6101382573
H 3.6202488033 2.1547627157 -3.3741067318
C 3.6121794915 2.4641351933 -0.6783304898
C 3.6194561819 1.2090333943 2.8281302788
H 3.6186798946 -2.1547743974 3.3740998198
C 3.6229032435 -0.0000074172 3.5190040424
H 3.6104362391 3.4048611208 -1.2330157306
C 3.6122626063 2.4641360711 0.6783302785
H 3.6206275468 2.1547645853 3.3741071478
H 3.6259737968 -0.0000098602 4.6101380399
H 3.6105751536 3.4048621926 1.2330150438
C -0.0096420558 -2.4641258049 0.6783253833
C -0.0089215852 -2.4641224705 -0.678326758
C -0.0095121558 -1.2318787197 -1.4269806611
C -0.0067797816 0.0000056975 -0.7147073946
C -0.0071350051 0.0000048305 0.7147090091
C -0.0104454809 -1.2318813927 1.4269802082
C -0.0173536329 -1.2090378541 -2.8281240884
C -0.0221377617 -0.0000089188 -3.5190044147
C -0.0190017988 1.209031737 -2.8281298263
C -0.0113237178 1.2318854502 -1.4269836398
H -0.0067443383 -3.4048501068 1.2330146384
H -0.0054071023 -3.4048467826 -1.233015025
C -0.0116068297 1.2318860726 1.4269845381
C -0.0183988942 -1.209039478 2.8281244647
H -0.0172937159 -2.1547731073 -3.3741009246
H -0.0251926853 -0.0000118684 -4.6101382099
H -0.020248694 2.1547629026 -3.3741065177
C -0.0121794066 2.464135171 -0.6783302535
C -0.0194561727 1.2090331057 2.8281304223
H -0.0186800352 -2.1547747292 3.3740997024
C -0.0229032939 -0.000007759 3.5190040917
H -0.010436115 3.4048611459 -1.2330154106
C -0.0122625311 2.4641359456 0.6783305176
H -0.0206275024 2.1547642545 3.3741073601
H -0.0259738626 -0.0000102842 4.6101380916
H -0.010575048 3.4048620299 1.2330153425

6a

24
0 1
C 1.2071590933 -0.7014813269 0.0931790331
C 1.2082021263 0.6930667387 0.089265999
C 0.0009919138 1.3911478697 0.0874071211
C -1.207232503 0.6948070826 0.0885457925
C -1.20826064 -0.699752322 0.0922582086
C -0.0010540781 -1.3978430305 0.094913395
H 2.1518876977 -1.247851538 0.099555462
H 2.1537428654 1.2380625825 0.0927235146
H 0.0017653785 2.4825315442 0.0896278547
H -2.1519730058 1.2412003714 0.0913450251
H -2.1538071874 -1.2447287303 0.0979022311
H -0.0018201924 -2.4891989759 0.1027189381
C 1.2083401723 -0.6929274 3.8116073953
C 1.2071431883 0.7016396577 3.8078383451
C -0.0011558631 1.3978680952 3.8053033347
C -1.2082702344 0.6996039442 3.80652064
C -1.2070807475 -0.6949570492 3.8097600563
C 0.0012150612 -1.3911755977 3.8121280895
H 2.1539617314 -1.2377981063 3.8099989762

H 2.1518348102 1.2481056205 3.802915397
H -0.0020648789 2.4892268001 3.7981351347
H -2.1538849047 1.2444549239 3.8002611038
H -2.1517650061 -1.2414428793 3.8058727833
H 0.0021252029 -2.4825582744 3.8102161691

6b
42
0 1
C 0.4779869493 1.4894522891 3.6324125042
C 1.1774539616 0.2997282584 3.3801167393
C 0.4588283846 -0.8896216536 3.2993512212
C -0.9321497352 -0.9166663176 3.4687813594
C -1.5992311982 0.2827037274 3.7073274223
C -0.9065477209 1.4995587422 3.7877140215
H 0.9896518845 -1.8235855984 3.0925187046
C -1.6724988602 -2.2243222677 3.373449784
H -2.6860657894 0.2763287596 3.8339355797
C -1.6589378427 2.7853927873 4.0147892628
H 1.0324305491 2.4300721313 3.7081798511
C 2.6726858619 0.3199877325 3.2014402082
H -2.7488662197 -2.0926219907 3.5481535273
H -1.2934319853 -2.9481958746 4.1099392543
H -1.534078833 -2.6710473976 2.3760637558
H -0.975251943 3.6172836372 4.229188478
H -2.3617782798 2.6924830542 4.855117389
H -2.2489096645 3.0551079106 3.1246699394
H 3.0508840355 -0.6700233989 2.9136705787
H 3.1783192771 0.6244958177 4.1305492423
H 2.9645552508 1.0352743406 2.4178332911
C 1.6195254951 -0.2883698222 -0.1082031513
C 0.9268354397 -1.5051855376 -0.1888420238
C -0.4576475504 -1.4951777677 -0.0329000855
C -1.1570138315 -0.3055946625 0.2202192019
C -0.4383734325 0.8837470594 0.3011811628
C 0.9525124459 0.9108966317 0.1311637039
H -1.0120900482 -2.4357764162 -0.1089341748
C -2.6522044357 -0.3258318299 0.3992799472
H -0.9691273847 1.8175976765 0.508709818
C 1.6929074444 2.2185001826 0.2269059012
H 2.7063063775 -0.2819007296 -0.2352563102
C 1.6791163831 -2.7909329851 -0.4168018632
H -3.0297814184 0.6632218155 0.6911465836
H -2.9442397601 -1.0441806689 1.1800041866
H -3.1582622134 -0.6262632081 -0.530939516
H 2.7690916458 2.0870176193 0.0509098632
H 1.5555570558 2.6642269856 1.2248932726
H 1.3129865087 2.9430697712 -0.5084510417
H 0.9953703962 -3.6225206968 -0.6321832973
H 2.2686868051 -3.0615184799 0.4733166231
H 2.3822849951 -2.6973866264 -1.2567779146

6c
96
0 1
C 0.6969087406 1.1938676516 4.8288284789
C 1.4200037955 -0.0124674492 4.8304075379
C 0.704893484 -1.2059275153 4.8301250631
C -0.7013892644 -1.2291551917 4.831793285
C -1.3775812946 -0.0131314365 4.8301334486
C -0.6942930099 1.2162886397 4.8307793077
H 1.2426634463 -2.1543698982 4.8311694746
C -1.4253751095 -2.5801089396 4.8263833079
H -2.4678943954 -0.0046274827 4.8307536407
C -1.5013421925 2.5193065492 4.8223744012
H 1.2494261753 2.1338238502 4.8283020906
C 2.9518828879 0.0349499472 4.8204171216
C -0.9998726664 -3.3984694288 6.0560275131
C -1.0434561558 -3.338545026 3.5448405205
C -2.9478954725 -2.4205597962 4.8603785592
C -0.6010081603 3.7573190545 4.8588765426
C -2.4275419097 2.561720857 6.0483250487
C -2.3435664415 2.5671794361 3.5371839183
C 3.4531742135 0.8169045653 6.0451021265
C 3.4125318589 0.7393611898 3.5338965109
C 3.5740031388 -1.363730159 4.8577883447
H 0.0842109194 -3.5795682346 6.0687020872
H -1.5056051925 -4.3764081807 6.0550407216
H -1.2667130927 -2.8715432183 6.9840805837
H 0.0323480517 -3.5677066549 3.5182859317
H -1.2808078584 -2.732107215 2.6569464206
H -1.5921555063 -4.2914046871 3.4803597282
H -3.2764259175 -1.8835477639 5.7625250523
H -3.4222072265 -3.4129004426 4.8674663293
H -3.3176278661 -1.8775870584 3.9786260022
H 0.0268172485 3.772403608 5.7620749938
H -1.2225600468 4.6647150814 4.8651935489
H 0.0555178082 3.8058108541 3.9782117259
H -1.8415413462 2.5293424768 6.9787481544
H -3.1275886402 1.7143736874 6.0588410729
H -3.0205668438 3.4893206751 6.0443479122
H -1.6964146269 2.4610256989 2.6525346072
H -2.8886620171 3.5216344095 3.4659596457
H -3.084541633 1.7542483297 3.5115639917
H 3.1343840458 0.3256276902 6.9763251072
H 3.068384158 1.8464498914 6.055927805
H 4.5529601884 0.8675904329 6.0390711231
H 3.0807514364 1.7880896333 3.5090025388
H 2.9936716243 0.2325802757 2.6505606371
H 4.5114789597 0.7323319511 3.459934301

H 3.2740792074 -1.9138094443 5.7620250447
H 4.6706171912 -1.2790127766 4.8630807136
H 3.2871984654 -1.9579217092 3.9782416563
C 1.4043650836 0.0043367374 0.0697938289
C 0.7208738073 -1.2249667784 0.0692826518
C -0.6703263241 -1.2023145699 0.071332488
C -1.3932334627 0.0041382087 0.0698353556
C -0.6779063479 1.1974817978 0.0698932382
C 0.7283830869 1.2204791734 0.0679473856
H -1.2229663944 -2.1422028553 0.0717601555
C -2.9251415316 -0.0431108504 0.0798213554
H -1.2154806068 2.14603063 0.068715074
C 1.4526130808 2.5713131944 0.0719797807
H 2.4946746429 -0.0043509103 0.0690876613
C 1.5277093593 -2.5281139705 0.0776351322
C -3.3858340356 -0.747388732 1.3664029431
C -3.4264477295 -0.8251441888 -1.1447918436
C -3.5472256732 1.355592312 0.0423727946
C 2.9751026594 2.41143965 0.0374598238
C 1.0716290196 3.331302971 1.352804331
C 1.0266724099 3.388440591 -1.1583835353
C 2.3696751878 -2.5763050489 1.3629308818
C 2.4541630074 -2.5704196654 -1.1481779678
C 0.627297172 -3.7660604933 0.0405512297
H -3.0540073877 -1.7960966279 1.3914714849
H -4.4847840534 -0.7403756931 1.4403277146
H -2.9670254249 -0.2404434899 2.2496731606
H -3.0417918127 -1.854740882 -1.1554548228
H -3.1075312397 -0.3340246679 -2.0760534461
H -4.5262413634 -0.8756830657 -1.1388266991
H -3.2604638353 1.9497632624 0.9219359055
H -4.6438360118 1.2708992813 0.0369988543
H -3.2472119806 1.9056542733 -0.8618471264
H 3.34505557 1.8689468044 0.9194275636
H 3.4496140908 3.40367733 0.0295639587
H 3.3031714487 1.8737870033 -0.8644699802
H 1.3114699469 2.7268531201 2.2413711801
H -0.0044669691 3.5588569259 1.3810717856
H 1.6190594735 4.2850493369 1.4147304949
H 1.2924976549 2.8601756981 -2.0859631584
H 1.5329912756 4.3660744812 -1.1589892378
H -0.0573145706 3.5701541481 -1.1704416626
H 1.7223943628 -2.4702064855 2.247486898
H 3.1107795519 -1.7634897807 1.3888265337
H 2.9146247266 -3.5308519555 1.4341272973
H 3.1543436872 -1.7231882295 -1.1583519357
H 1.8683295734 -2.537731842 -2.0786946758
H 3.0470218952 -3.4981223588 -1.1442852383
H -0.0292362525 -3.8153594211 0.9211809809
H 1.2488861179 -4.6734361181 0.0336224432
H -0.0005080431 -3.7806349771 -0.8626727157

6e

120
0 1
C 0.028900505 1.401253411 4.3862787597
C -1.1746895732 0.6957831822 4.3714446326
C -1.2038176928 -0.7047215192 4.3620980242
C 0.009136805 -1.3937245055 4.3862461316
C 1.2365029624 -0.7191309528 4.3997411828
C 1.2269130779 0.6759133756 4.4092298057
C 0.0393539336 2.9140105718 4.3696653654
C -2.5160683993 -1.4573611187 4.3295855924
C 2.5424552302 -1.4829130635 4.3969754726
C 2.8050576516 -2.1969913281 5.732028192
C 4.149625349 -2.9249767848 5.7209274142
C 4.2469759498 -3.9031854766 4.5496732948
C 3.9805652544 -3.2000052821 3.2181104343
C 2.6307026062 -2.4842661907 3.2361168596
C -3.2523357465 -1.4024569794 5.6774373574
C -4.5502833803 -2.2101150512 5.6380360242
C -5.4606898 -1.75365023 4.4973542646
C -4.7304367689 -1.7989938388 3.1545314938
C -3.4427890969 -0.9779287437 3.2036981585
C 0.8662548282 3.4749540824 3.2036618618
C 0.820768923 5.0017337158 3.1684528774
C 1.2962814116 5.5960620746 4.4947232507
C 0.4908484983 5.0401977261 5.6696898592
C 0.5256967992 3.5118252075 5.6989138929
H -2.1131805413 1.2571372811 4.365768369
H 2.1821349581 1.2085904985 4.4306300011
H -0.0070469953 -2.4874285404 4.3911091756
H -2.9026037408 -1.0178949058 2.2454834039
H -3.7022771978 0.082441482 3.3735743188
H -2.5895678484 -1.7676091355 6.476881736
H -3.4783414402 -0.3469452633 5.9120902099
H -0.0872452381 3.1227546208 6.5263354025
H 1.5593719502 3.1691128817 5.8835791529
H 1.9162022335 3.1484007202 3.314246373
H 0.5024639077 3.0399681905 2.2605256455
H 1.827329859 -3.2353275228 3.3432214828
H 2.4417645527 -1.9588916562 2.2874979792
H 1.9920367274 -2.9232530541 5.9099323808
H 2.762109696 -1.4685928342 6.5561945474
H -5.3851707817 -1.4341090332 2.3482332992
H -5.0751913762 -2.1339582164 6.6023546472
H -6.3709640137 -2.3712001985 4.4628472989
H -5.7886385933 -0.7173797953 4.6913160654
H 0.8669787189 5.4475841273 6.620354595
H 1.4313289012 5.3824675484 2.3352236157

H 1.2316147933 6.6944151826 4.4705692538
H 2.3618439159 5.3448854306 4.638067094
H 4.0187815268 -3.9216921717 2.3877510278
H 4.3053604072 -3.4510389305 6.6749814651
H 5.2335817598 -4.3906438656 4.5378973842
H 3.499787515 -4.7043745742 4.6869396819
H -1.0036837348 3.248737146 4.2204582723
H 3.3538938357 -0.7456678421 4.253967892
H -0.5576653259 5.3733627216 5.5772336389
H -0.2165145329 5.3281013689 2.9750365431
H 4.7793195189 -2.460459011 3.0310741659
H 4.9599336677 -2.1798856181 5.6362813163
H -2.2797444534 -2.5184552722 4.1275751947
H -4.4822608359 -2.8472385063 2.910413799
H -4.304008466 -3.2770784137 5.497077866
C 1.2028513752 0.7030984902 0.1618849982
C -0.0103102157 1.3917767904 0.1376901745
C -1.2374734918 0.7168809641 0.1239725702
C -1.2275171039 -0.6781776376 0.1139526469
C -0.0293356471 -1.4031877556 0.136935324
C 1.174080689 -0.6973850619 0.1521529042
C 2.5147590915 1.4563406956 0.1946375176
C -2.5436918365 1.4802005874 0.1273334489
C -0.0392473186 -2.9159526223 0.1535077829
C -0.8670064965 -3.4773550923 1.3186905517
C -0.820493476 -5.0041047977 1.3539717579
C -1.2944261445 -5.5987401383 0.027269242
C -0.4883630462 -5.0423250809 -1.1470066657
C -0.52408365 -3.5139692489 -1.1762003794
C -2.6307707367 2.4835453352 1.2865526619
C -3.9812274833 3.1981173939 1.3055063345
C -4.250136202 3.8989511909 -0.0268050554
C -4.153620687 2.9190249456 -1.196699325
C -2.8085754526 2.1919171851 -1.2085259283
C 3.2489629491 1.4053943656 -1.1545185033
C 4.5462903979 2.2140384825 -1.1151795364
C 5.4588435312 1.7556114975 0.0229960492
C 4.7305838957 1.7969392684 1.3670327332
C 3.4436532901 0.974726603 1.3177828428
H 0.0055923923 2.4854782345 0.1332119917
H 2.1126643868 -1.2585414385 0.1579479354
H -2.1826223555 -1.2110607317 0.0924551271
H -2.766333323 1.4622108191 -2.0315712162
H -1.9962102093 2.918360567 -1.38870247
H -2.4394657768 1.9601384586 2.2358031841
H -1.8283762078 3.2352319294 1.176605029
H 2.9051315217 1.0115488432 2.2770949192
H 3.7040774037 -0.084864098 1.1445551883
H 3.4753878959 0.3506213173 -1.392090579
H 2.5846210802 1.7719710472 -1.9520018568
H -1.5577894812 -3.1718163096 -1.361746039
H 0.0893653982 -3.1245185485 -2.0030672053
H -1.9170782001 -3.1516464557 1.2068840809
H -0.5046322519 -3.0420658703 2.2622537869
H -4.3110237283 3.4435310945 -2.1513363825
H -4.0188314959 3.9211197773 2.1347465711
H -5.2371366181 4.3855943636 -0.0144057988
H -3.5038254628 4.7005614105 -0.1663638922
H 5.3868956263 1.4307113129 2.171431502
H 5.0697747412 2.1406519844 -2.080486482
H 6.3685888968 2.3739320232 0.0576581742
H 5.7874770716 0.7201350689 -0.174023605
H -0.8634621655 -5.4499028914 -2.0979971273
H -1.4315515198 -5.3852587259 2.1866414297
H -1.2290311414 -6.6970493478 0.0514658061
H -2.360035609 -5.3482935248 -0.1169947051
H 2.2780549252 2.5167631324 0.399714168
H 1.0037939903 -3.2502362234 0.3036872556
H 4.4817569835 2.844338764 1.6141033428
H 4.2993261586 3.2804475766 -0.9712649394
H 0.5602762803 -5.3748570994 -1.0537004708
H 0.2168367231 -5.3297655534 1.5483262822
H -3.3546118993 0.7428533543 0.272768374
H -4.9632801356 2.1734840914 -1.1098377358
H -4.7790766923 2.4581966676 1.4949065926

6f

84
0 1
C 1.1305081116 -0.8513428553 3.8699772057
C 1.2791001422 0.539594749 3.8710062111
C 0.1660402524 1.3887052314 3.8709335286
C -1.112841466 0.8218221566 3.871474187
C -1.2917124915 -0.5665615812 3.8710961013
C -0.161323358 -1.3906352317 3.8713618605
H 2.2817835979 0.9730412928 3.874332556
H -1.9895438105 1.4734453802 3.8750936635
H -0.287279914 -2.4756709151 3.8751787967
C 0.3385425374 2.8640112751 3.8539284402
C 1.3420943721 3.4543484491 3.0726792797
H 1.9867434761 2.8181371445 2.4621242866
C 1.4882126592 4.8377506747 3.0268269498
H 2.25993455 5.2792703205 2.3943084053
C 0.637005628 5.6568991531 3.7687306957
H 0.749845415 6.7415623976 3.7312430702
C -0.3620725216 5.0820909562 4.5525304084
H -1.0265245802 5.7151759348 5.1430374571
C -0.5114201082 3.6969604215 4.5931132572
H -1.2831253521 3.2514097808 5.2239584299
C -2.6556163715 -1.1548502162 3.8541006633

C -2.9520817064 -2.3068943545 4.5940760183
H -2.1804424055 -2.7518458742 5.2254384027
C -4.2263059099 -2.8701856452 4.5535938821
H -4.442549008 -3.7616555975 5.1447918631
C -5.223370121 -2.2929814265 3.768995214
H -6.2191505846 -2.7375761965 3.7317170885
C -4.9393909621 -1.1468294785 3.0262116437
H -5.7075989447 -0.6997173785 2.3933426832
C -3.6683428956 -0.5815183613 3.0720830621
H -3.4393866235 0.2946097519 2.4612519071
C 2.3216660888 -1.7387324519 3.8510389912
C 2.3299149596 -2.9019565064 3.0682514244
H 1.4555260545 -3.141847965 2.4591547549
C 3.4547248067 -3.7203223988 3.0200296957
H 3.4503851254 -4.6086053792 2.3863747501
C 4.5905101422 -3.3941403011 3.7613711186
H 5.473188331 -4.0344310479 3.7222359602
C 4.5933759066 -2.2426735944 4.546894876
H 5.4745303885 -1.9848976143 5.1368913298
C 3.4688202524 -1.4203701254 4.5895018596
H 3.469560804 -0.5299435921 5.2213173836
C 1.2920404435 -0.5624132201 0.2233329681
C 1.1131816455 0.8259616129 0.2244026524
C -0.1657248774 1.3928349554 0.2255093163
C -1.2787861451 0.5437164847 0.2243088766
C -1.1301873969 -0.8472276269 0.2238511947
C 0.1616517821 -1.3864694628 0.2221255281
H 1.9899309158 1.4775304272 0.2214857657
H -2.2814611898 0.977200337 0.2212185686
H 0.2877010895 -2.4714778668 0.2174259389
C -0.3382517279 2.8681150012 0.2444205035
C 0.5122014652 3.7021156066 -0.4930109593
H 1.2842712418 3.2574696343 -1.1240475658
C 0.362892563 5.0871943105 -0.4505257415
H 1.0277469758 5.7210893339 -1.0397096497
C -0.6366800677 5.6609141843 0.3334348994
H -0.7496570944 6.7455142364 0.3722803526
C -1.4884042979 4.8407105491 1.0735874017
H -2.2606773385 5.2813672548 1.7060338553
C -1.3422932939 3.4573670016 1.0258865645
H -1.9876810679 2.8203331786 1.6348215722
C -2.3212422273 -1.7347800731 0.2415960651
C -2.3292203829 -2.8991451171 1.022694074
H -1.4548146044 -3.1395619242 1.6315514733
C -3.4538275139 -3.7178461843 1.0697614452
H -3.4492704868 -4.6070487828 1.7021237641
C -4.5897202087 -3.3908097653 0.3289616274
H -5.4722619723 -4.0313433446 0.3672021541
C -4.5928883181 -2.2381720523 -0.4548417794
H -5.474144686 -1.9797077846 -1.0443849402
C -3.4685028574 -1.4155704971 -0.4963319767
H -3.4694738422 -0.5242378303 -1.1268680155
C 2.6558673628 -1.1508972775 0.2397988457
C 2.9521620626 -2.3021661738 -0.5014635772
H 2.1804827512 -2.7462182983 -1.1334093676
C 4.2262418691 -2.8657994139 -0.4614931141
H 4.4423447464 -3.7566383976 -1.0536926659
C 5.2233354286 -2.2897570907 0.3239232853
H 6.2189950016 -2.734652313 0.3608482006
C 4.9395447578 -1.1443809057 1.067964659
H 5.7077947859 -0.6981627375 1.7014121937
C 3.6686471224 -0.5786743507 1.0225533414
H 3.4399453302 0.2969697716 1.6341751529

6d

168
0 1
C -1.3742687233 -0.3251881287 4.7485071768
C -0.9426510774 1.0132873924 4.7508271456
C 0.4078581186 1.3497250093 4.7504052837
C 1.3511750401 0.3066391513 4.7547409206
C 0.9673786895 -1.0310067846 4.7498493423
C -0.4076727733 -1.3263832564 4.7531705102
C -2.870024757 -0.6245199051 4.6936988855
H -1.6934488929 1.8036076157 4.7404383359
C 0.8974025299 2.794355649 4.694602508
H 2.410919134 0.5618802463 4.7473267627
C 1.9739432535 -2.176862491 4.6883192099
H -0.7165908311 -2.3717044162 4.7447215297
C -3.6031610676 0.0057938321 5.8999444267
C -3.1723162889 -2.1347755723 4.6911489928
C -3.4592744942 -0.0190058906 3.3986316029
C 1.7174915076 2.9980302269 3.3994361759
C 1.809669683 3.116318735 5.9004159018
C -0.2582337052 3.8124410976 4.6895817483
C 3.4333288493 -1.6843250214 4.6835207251
C 1.7382561346 -2.9812840318 3.3891999344
C 1.7991203632 -3.1347432144 5.889165705
H -3.1975239185 -0.4202160707 6.832628105
C -5.1151859403 -0.2461974695 5.8089884744
H -3.4092521746 1.0899159505 5.930466266
H -2.7659817804 -2.5948382252 5.6074592409
H -2.6688230899 -2.6092309266 3.8336365444
C -4.6817983669 -2.3961248694 4.603735922
H -3.2713895305 1.0670959216 3.3823019921
C -4.9675581664 -0.2768750523 3.3036886685
H -2.9260586329 -0.4451195963 2.5313855341
H 2.5640325682 2.2920185738 3.3857981261
C 2.2490676628 4.4325368061 3.3008703396
H 1.0823510378 2.747028722 2.5324936572

H 2.6507710918 2.4054565166 5.9336348914
H 1.2371920901 2.9816216382 6.8332074101
C 2.3491210911 4.5511178174 5.8058129899
H -0.9211780687 3.6121431425 3.8327651549
C 0.2715363713 5.2496088087 4.5985449361
H -0.8599111305 3.6932286091 5.6062307044
H 3.5897984391 -1.0055196555 3.8299637196
C 4.4132211237 -2.8608430539 4.5838765354
H 3.6323108226 -1.1085244254 5.6029278738
H 1.8362916224 -2.3005949226 2.5260277669
H 0.7036337107 -3.3614104203 3.3754563483
C 2.7147548728 -4.1581242375 3.2818991931
H 1.9700087923 -2.5769545022 6.8249093602
C 2.772318252 -4.3183989455 5.7859487638
H 0.7631942937 -3.5084739544 5.9219824486
C -5.3837573873 -1.7567349826 5.8086278843
C -5.2298043281 -1.788445343 3.3053686996
C -5.6626386218 0.3674430157 4.5117326756
H -5.6116197654 0.2184620316 6.6752512257
H -4.8582279922 -3.4833573217 4.6055108293
H -5.3541781896 0.1689566644 2.3718926284
H -6.4682150909 -1.949105926 5.7618165062
H -5.0119438727 -2.2063152185 6.7438228759
H -6.3106262049 -1.9874670133 3.2194719985
H -4.7406586927 -2.2585728664 2.4350508027
H -6.7519050166 0.2095492254 4.4507348136
H -5.4906991111 1.4568019222 4.5067475418
C 3.1546672252 4.7146775299 4.5083480827
C 1.1764648176 5.5402419634 5.8027376248
C 1.0719301765 5.4161830776 3.2997020979
H 2.8288875707 4.541641913 2.3689708734
H 2.9998121386 4.7500979846 6.6717138763
H -0.5812372954 5.9467514352 4.5984362391
H 3.563161447 5.7365873051 4.4451045266
H 4.0117187235 4.0206246557 4.5052099968
H 1.5532434977 6.5750462861 5.7530864294
H 0.600893112 5.4462969702 6.7380896144
H 1.4404710953 6.4513018654 3.2108670339
H 0.4200530473 5.2254793673 2.4299675818
C 4.2150586449 -3.7967583839 5.7831938734
C 4.1551160242 -3.6299020754 3.281224415
C 2.5089397945 -5.090373643 4.484558584
H 5.4432224758 -2.4705965516 4.5841294531
H 2.5178921594 -4.7096189128 2.3472205492
H 2.6210648916 -4.9863875301 6.6483196721
H 4.9231299806 -4.6398170988 5.7274661584
H 4.4228834263 -3.256571516 6.7212752405
H 4.8673946414 -4.4658629279 3.1863883494
H 4.3142205423 -2.9650261563 2.4150185575
H 3.1897428203 -5.95456623 4.4150954439
H 1.4794206912 -5.4857618253 4.4812728604
C -0.9683572945 1.0306131377 -0.0538514601
C 0.4067320837 1.3258053083 -0.0574884223
C 1.3731894652 0.324479039 -0.0529936507
C 0.9413833081 -1.0139310001 -0.0552586215
C -0.4091696169 -1.3501944526 -0.0545441931
C -1.3523512319 -0.306979676 -0.0586839748
C -1.9747238233 2.1766329425 0.0079258197
H 0.7157888344 2.371080161 -0.0491158734
C 2.8689960792 0.6235815906 0.0016580795
H 1.692069141 -1.8043589889 -0.0450312646
C -0.8988269964 -2.794782327 0.0014499965
H -2.4121386512 -0.5620550567 -0.0510431082
C -1.7379107982 2.9816714708 1.3064980372
C -3.4341476869 1.6842713373 0.014074515
C -1.8006729016 3.1339491499 -1.1934684508
C 3.6017916463 -0.0062736931 -1.2050392995
C 3.458421338 0.0172576013 1.2962569174
C 3.1715351278 2.1337784827 0.0049159492
C 0.256711354 -3.8129621394 0.0066456444
C -1.8111031375 -3.1168987766 -1.2043240208
C -1.7190276731 -2.9981459305 1.2965893446
H -1.8350549715 2.3014329977 2.1701459728
C -2.7142371772 4.1586192385 1.4140248629
H -0.7032825149 3.3618009797 1.3190088324
H -3.5899613355 1.0059331115 0.8681114573
H -3.6339267808 1.1080646997 -0.9049026908
C -4.413833191 2.8609495475 0.1139026095
H -0.7647113231 3.5075133455 -1.2272310735
C -2.7736627868 4.3177713596 -1.0900593935
H -1.9723371939 2.5757780741 -2.1288439625
H 3.4076439049 -1.0903423061 -1.2360374436
C 5.1138753994 0.2454170454 -1.1142446484
H 3.1960650992 0.4202678616 -2.137439939
H 3.2705528277 -1.0688616752 1.3117782329
H 2.9253465652 0.4427465155 2.163912337
C 4.9667501137 0.2749093411 1.3910922653
H 2.7651177356 2.5943737853 -0.9110882832
C 4.6810786388 2.3948389763 0.0921528295
H 2.6682633331 2.6078656677 0.8627636045
H 0.8583325253 -3.6939980852 -0.9100743322
C -0.2731926999 -5.2500732801 0.0979731356
H 0.9197843345 -3.6126614114 0.8633545806
H -1.2386017929 -2.9824489485 -2.1371349312
H -2.6521153468 -2.4059309697 -1.2376718377
C -2.3506954384 -4.5516337118 -1.1094434137
H -1.0840797284 -2.7467269256 2.1635675931
C -2.2506593486 -4.4325846792 1.3954824788
H -2.5655890279 -2.292157318 1.309904433
C -4.154656111 3.6305756463 1.4160113508

C -4.2164587028 3.7962836268 -1.0859943363
C -2.5092401438 5.0903093927 0.2107803064
H -2.5166298874 4.7105408346 2.3482943878
H -5.443877294 2.4708157666 0.1146126481
H -2.6229663084 4.9853402669 -1.9528517076
H -4.8667405067 4.4666849923 1.5110034969
H -4.3132258016 2.9661213683 2.2826362416
H -4.9243987752 4.639444285 -1.0301369539
H -4.4250357811 3.2556833836 -2.0236706392
H -3.1899217745 5.9545867665 0.2803717931
H -1.4796877342 5.4856140075 0.2131235336
C 5.6615123252 -0.3689233606 0.1826018601
C 5.2292376833 1.7864354306 1.3901128843
C 5.382680923 1.7559169867 -1.1131965105
H 5.610066494 -0.2188943674 -1.9808336099
H 5.3534670677 -0.1714402469 2.322600539
H 4.8576913724 3.4820411282 0.090868011
H 6.7508102969 -0.2111917803 0.2434435802
H 5.4894356657 -1.4582622788 0.1871091711
H 6.3101089222 1.9852300419 1.4759061998
H 4.7403354756 2.2562089686 2.2607564219
H 6.4671795415 1.9480907471 -1.0665193663
H 5.010746893 2.2060155386 -2.048094459
C -1.0735832809 -5.4163054022 1.396866535
C -1.1781366558 -5.5408743931 -1.1061576453
C -3.1562629311 -4.7149004792 0.1880492504
H 0.5795274902 -5.9472816992 0.098231999
H -3.0014057606 -4.7507108722 -1.9753073995
H -2.8305046801 -4.5414715649 2.3273986747
H -1.4421772136 -6.4513822703 1.4859593321
H -0.4216849191 -5.2254186693 2.2665478656
H -1.5550297834 -6.5756273625 -1.0563014847
H -0.6025396499 -5.4471837678 -2.0415194034
H -3.5648125509 -5.7367774914 0.2514663997
H -4.0132764011 -4.0207997007 0.1910573882

10
96
0 1
C 4.6930419157 -1.4794427668 1.2700048025
C 4.2663859361 -0.7464047919 0.0000000363
C 4.7834028908 0.6989114709 -0.0000000092
C 4.3458536471 1.4287050213 1.2690482195
C 4.7720337483 0.699980083 2.5427569766
C 4.259613053 -0.7482044127 2.5387184175
C 4.6828234676 -1.485465602 3.809623835
C 4.2336078534 -0.7491454254 5.0707290446
C 4.733425429 0.6955746942 5.0777611451
C 4.3172816961 1.433274174 3.8057073139
H 5.794423304 -1.5844373663 1.2727392553
C 4.6930418748 -1.4794428434 -1.2700047003
C 4.3458536891 1.4287049547 -1.2690482966
H 3.2426852617 1.5239791355 1.2640713448
H 5.783933312 -1.5817368511 3.8118920969
H 3.1302782015 -0.7499304121 5.111396699
H 5.8350352305 0.6951767288 5.148593138
H 3.2156593245 1.5263710248 3.7783317275
C 4.772033789 0.699979925 -2.5427569983
C 4.259613014 -0.7482045397 -2.538718347
C 4.3172818005 1.4332739714 -3.8057073869
C 4.7334254831 0.695574384 -5.077761168
C 4.2336077595 -0.7491456766 -5.0707289768
C 4.6828233414 -1.4854658263 -3.8096237378
H 4.2801246479 -2.5026592689 -1.2686088156
H 4.7473156879 2.4559109161 -1.2709441828
H 3.2156594355 1.5263709071 -3.7783318165
H 5.7839331777 -1.5817371711 -3.8118920171
H 4.2801247184 -2.5026592043 1.2686089773
H 3.1596635695 -0.6884137511 0.000000049
H 5.8905291362 0.6475895056 0.0000000073
H 4.7473156158 2.4559109949 1.270944043
H 5.879575124 0.6520809268 2.5546366166
H 3.1526782355 -0.6924475336 2.533544706
H 4.2777849239 -2.5104776518 3.7970065656
H 4.5798028895 -1.2800420939 5.9703410016
H 4.36048989 1.2259462814 5.9675230553
H 4.7166032308 2.4600489015 3.8061810665
H 5.7944232596 -1.5844374753 -1.2727391623
H 3.2426853062 1.5239791012 -1.2640714523
H 5.8795751623 0.6520807091 -2.5546366117
H 3.1526782003 -0.6924475912 -2.5335446069
H 4.7166034135 2.4600486679 -3.8061812065
H 5.8350352847 0.6951763052 -5.1485931594
H 3.1302781057 -0.7499305279 -5.111396599
H 4.2777847087 -2.5104778397 -3.7970064079
H 4.3604899918 1.2259459519 -5.9675231097
H 4.579802712 -1.2800424257 -5.9703409191
C 0.2541461195 -1.428705007 1.2690482039
C -0.1834031208 -0.6989114458 -0.0000000311
C 0.3336138085 0.7464047741 0.0000000202
C -0.0930421713 1.479442776 1.2700047959
C 0.3403867242 0.7482043922 2.5387184006
C -0.1720340335 -0.6999800513 2.5427569554
C 0.2827178886 -1.4332741733 3.8057072992
C -0.133425905 -0.6955746604 5.0777611295
C 0.3663919449 0.7491453524 5.0707290451
C -0.0828235694 1.485465612 3.8096238527
H 1.3573145027 -1.5239791138 1.2640713702
C 0.2541460839 -1.4287049415 -1.2690483194
C -0.0930421168 1.4794428488 -1.2700047302

H -1.1944235548 1.5844373904 1.2727392643
H 1.384340261 -1.5263710842 3.7783318351
H -1.2350357119 -0.6951765452 5.148592988
H 1.4697215988 0.7499299944 5.1113966779
H -1.1839334027 1.5817370242 3.8118921543
C 0.3403867918 0.7482045176 -2.5387183623
C -0.1720340471 -0.6999798944 -2.5427570202
C -0.0828233996 1.4854658332 -3.8096237931
C 0.3663921012 0.7491456052 -5.0707290093
C -0.1334258908 -0.695574353 -5.0777611984
C 0.2827178301 -1.4332739716 -3.8057074075
H -0.1473159503 -2.4559108798 -1.2709442152
H 0.3198751689 2.5026592434 -1.2686088216
H -1.1839332249 1.5817373391 -3.8118921235
H 1.384340195 -1.5263709681 -3.7783319441
H -0.1473158835 -2.4559109577 1.2709440282
H -1.2905293722 -0.6475895114 -0.0000000158
H 1.4403361801 0.6884138368 0.0000000361
H 0.3198750865 2.5026591821 1.2686089521
H 1.447321541 0.6924475303 2.5335446382
H -1.2795754117 -0.6520808324 2.5546364927
H -0.1166037333 -2.4600488615 3.8061810596
H 0.2395094975 -1.2259463456 5.9675230338
H 0.0201970801 1.280042098 5.9703410226
H 0.322215114 2.5104776083 3.7970065744
H 1.3573144642 -1.5239790826 -1.2640715014
H -1.1944234967 1.5844374937 -1.2727392182
H 1.4473216046 0.692447586 -2.5335445574
H -1.2795754227 -0.6520806135 -2.554636548
H 0.3222153688 2.5104777948 -3.7970064483
H 1.4697217575 0.7499301148 -5.1113965967
H -1.2350356955 -0.6951761292 -5.1485930785
H -0.1166038702 -2.4600486291 -3.8061812412
H 0.0201973282 1.2800424305 -5.9703409761
H 0.2395094931 -1.2259460135 -5.9675231256

11
84
0 1
C 4.7364381897 -2.5255612629 0.7355756859
C 4.2980059502 -2.522846934 -0.7253377431
C 4.7721631084 -1.2704507367 -1.4614127645
C 4.3141121892 -0.0002702253 -0.7301599602
C 4.7514782082 -0.0002219909 0.7407544578
C 4.275052583 -1.2663736198 1.4666744903
C 4.3069711269 -1.2592247915 -2.9176629809
C 4.7596235642 -0.0005661368 -3.6541404325
C 4.3078569847 1.2585687077 -2.9179217688
C 4.7726545261 1.2696612973 -1.461535283
H 5.8386757286 -2.5813018383 0.7884033697
H 4.3503539232 -3.4187783712 1.2529809999
H 3.194277911 -2.5620696713 -0.7755291522
H 4.6666143899 -3.4226630558 -1.2430589733
H 5.880566973 -1.2677476577 -1.4542817328
H 3.2031970553 -0.0001906265 -0.7402027943
C 4.2751326528 1.2660842417 1.4664819317
C 4.7086308526 -1.2592938655 2.932233239
H 3.2027052131 -1.3145106888 -2.9306804827
H 4.6708571938 -2.1623859578 -3.4329672836
H 4.3755723863 -0.0005290908 -4.6858416575
H 5.8608153005 -0.0009649029 -3.7261706219
H 3.2036495351 1.3149836349 -2.9314391079
H 4.6727753832 2.1613229954 -3.4332197774
C 4.2988289798 2.5223475993 -0.7257322396
H 5.8624522032 -0.0002812666 0.760932768
H 3.1671120519 -1.247008769 1.4406958918
H 5.881055077 1.2665264863 -1.4541706455
H 3.1671967137 1.2468098003 1.4403093255
C 4.7085267745 1.2591656026 2.9320963796
H 5.8114768544 -1.3183683606 2.9735675756
H 4.3257379332 -2.1611321049 3.4370226984
C 4.2404142797 -0.0000450774 3.6579935451
H 3.195147766 2.5620513493 -0.7763711285
H 4.6680017378 3.4219315495 -1.2434580527
C 4.7368199058 2.5251218623 0.7353147406
H 4.3254946333 2.16102446 3.4367481697
H 5.8113620789 1.3183690913 2.9735407485
H 4.5973272199 0.0000393691 4.6988948997
H 3.1374150312 -0.0001085661 3.7038585203
H 4.3507331058 3.4184586832 1.2525164431
H 5.8390495672 2.5807158718 0.7884414126
C 0.301023474 -2.5225020835 0.7253490906
C -0.1372884124 -2.5250109837 -0.7356112805
C 0.3245216004 -1.2659815275 -1.4667063535
C -0.1514618911 0.0003325942 -0.7407459428
C 0.2860750273 0.0001329165 0.7301328801
C -0.1724796331 -1.2698495243 1.4614001774
C -0.1091104956 -1.2587542083 -2.9322519774
C 0.3593247832 0.0004063564 -3.6580328292
C -0.1084143683 1.2597210694 -2.9320736336
C 0.3251808755 1.2665047106 -1.4665184334
H 1.4047095011 -2.5624435027 0.7756696915
H -0.0681892236 -3.422092301 1.2430345502
H -1.2395413044 -2.5803541716 -0.7885170266
H 0.2484984567 -3.418366367 -1.2530016443
H 1.4324707929 -1.2469203966 -1.4407818053
H -1.2624353174 0.0006233582 -0.7608115168
C -0.171954722 1.2702173652 1.4615606986
C 0.2925262837 -1.2589298996 2.9177221597
H -1.2119709833 -1.3175841582 -2.97354388

H 0.2735536013 -2.1606695834 -3.4370808175
H 0.0023115815 0.0005784692 -4.6989001818
H 1.4623185147 0.0001122316 -3.7040106564
H -1.2112413354 1.3191735371 -2.9733675311
H 0.2747539432 2.1614972415 -3.4367705862
C -0.1360129565 2.5256758283 -0.7352713447
H 1.3969962551 -0.0001066437 0.739953608
H -1.2808829342 -1.2665952552 1.4542164024
H 1.433116361 1.2468924532 -1.4405043003
H -1.2803586379 1.2674521972 1.4543221647
C 0.2929921876 1.2589231304 2.9178961366
H 1.396741182 -1.3152033417 2.93109055
H -0.0722145217 -2.1617970735 3.4329479824
C -0.1593601979 0.000029443 3.6541626407
H -1.238233111 2.5816106828 -0.7882413739
H 0.2502762487 3.418900717 -1.2525152241
C 0.3021851235 2.5227524059 0.7257162209
H -0.0714365097 2.1618534176 3.4332327779
H 1.397225254 1.314802147 2.9313028063
H 0.2247469896 -0.0001082822 4.6858437313
H -1.2605474678 0.0002253636 3.726260902
H -0.0666399282 3.4224463803 1.2434974388
H 1.4058850719 2.5621182539 0.7762030195

9a
36
0 1
C 1.2695126759 0.734086584 4.7648643248
C 1.2676472877 -0.7269021399 4.3118778199
C 0.0066466651 -1.4548757836 4.7806151891
C -1.2584725922 -0.7277912209 4.3218172543
C -1.2578582064 0.7330178186 4.7754056064
C 0.0035904996 1.4609845578 4.3079275929
H 2.165156248 1.2509476972 4.3868392396
H 1.3266103536 0.7689999472 5.8671461191
H 2.1679332191 -1.2434630357 4.6776786452
H 1.3134032457 -0.7615325135 3.2088211402
H 0.0109349563 -1.5089866163 5.8835831152
H 0.0056068131 -2.4931834814 4.4144244082
H -2.1555245685 -1.2452098225 4.6943080107
H -1.3126443262 -0.7620432156 3.2191200949
H -1.305685309 0.7674145149 5.8781456399
H -2.1571154075 1.2492198811 4.4051560797
H 0.0047470922 2.4995311376 4.6720910322
H -0.0011488686 1.5163697653 3.2048403377
C 1.2580944759 0.7249975973 0.3899746241
C 1.2585770402 -0.7325326243 -0.0740434706
C -0.0061223251 -1.4623469604 0.3818506718
C -1.2685795929 -0.7315411884 -0.078290792
C -1.2685755446 0.7256607187 0.3865693872
C -0.0044659583 1.4565749304 -0.0689390667
H 2.1570334493 1.2446433387 0.0252118757
H 1.3075045683 0.7511748867 1.4930737687
H 2.1553821652 -1.2522022099 0.2973093362
H 1.3121133922 -0.7593348556 -1.1767285787
H -0.0081776461 -1.5231765643 1.4847257343
H -0.0059192865 -2.4989018009 0.0121187698
H -2.1670643657 -1.2507557022 0.289560458
H -1.3181383256 -0.7576655225 -1.1811839763
H -1.3210263156 0.750875547 1.4895607714
H -2.1663045263 1.2459374723 0.0197397801
H -0.0045512163 2.4917023348 0.3062233064
H -0.0031197659 1.5203065281 -1.1713962491

9b
54
0 1
C 0.7233740835 -1.2518048602 4.3457747242
C -0.7351034458 -1.2767470051 4.8071289633
C -1.4468298545 -0.0027915775 4.3476067834
C -0.7386100015 1.2734380386 4.8062162172
C 0.720006198 1.2523287516 4.3451516693
C 1.4715068706 0.001390375 4.8045464552
H -1.4870778665 -0.0033853986 3.2408827126
H -0.7408327784 1.2795060333 5.9121073825
H 0.7385719205 1.2863321201 3.2383490925
H 1.4787319757 0.0016700068 5.9104324572
H 0.7417575578 -1.2860117106 3.2389917562
H -0.7371289825 -1.2819820768 5.9130237355
H -2.4921873573 -0.0040668931 4.6994515047
C -1.4626129439 2.5224451566 4.3146503312
H 1.2418937427 2.1587030081 4.6954251726
C 2.9147551611 0.0032512179 4.3117928835
H 1.24787278 -2.1566139776 4.6961896408
C -1.4556608211 -2.5282003601 4.316707747
H -0.9633379473 3.4381548161 4.6642337379
H -1.4797263933 2.5489957778 3.2130663742
H -2.5044930032 2.5457541471 4.6666954593
H 3.4585677234 -0.8862709668 4.6625135075
H 2.9454785573 0.0028404997 3.2101882076
H 3.4559990421 0.8946357357 4.6617677086
H -1.4724266796 -2.5562554858 3.2151453306
H -0.954082841 -3.4421729326 4.6675444857
H -2.4975721317 -2.5538580302 4.6685040254
C -0.723458058 -1.2503023498 0.2555736694
C -1.4715942328 0.0026289389 -0.2039548092
C -0.7199123181 1.2540614586 0.2538054682
C 0.7386443148 1.2745419582 -0.2075091201
C 1.4467492483 -0.0012985912 0.2523615281
C 0.7349204803 -1.2755870029 -0.2060426918

H -0.7381980706 1.2898795249 1.3605576687
H 0.7407162798 1.2794489736 -1.3134053942
H 1.4868023654 -0.0006888455 1.3590831626
H 0.7366702004 -1.2815783155 -1.3119376688
H -0.7417950145 -1.2840546818 1.3623770202
H -1.479171647 0.0018789117 -1.3098407812
H -1.2418154062 2.1599735029 -0.0976596873
C 1.4628618744 2.5240027374 0.282602891
H 2.4921388797 -0.0029978943 -0.0993740126
C 1.4556210355 -2.5267254862 0.284974183
H -1.2480549982 -2.1552554926 -0.0943371446
C -2.9147189802 0.0050877421 0.289170502
H 2.5047326964 2.5467111591 -0.069513217
H 1.480045598 2.5520316073 1.3841650055
H 0.9637473901 3.4393675189 -0.068115762
H 0.9537631789 -3.440916344 -0.0648968721
H 1.473004674 -2.5539620236 1.3865402173
H 2.4973329943 -2.5527506167 -0.0673832994
H -2.9451982764 0.0060343099 1.3907761497
H -3.458619396 -0.8848595083 -0.0603371488
H -3.4560463774 0.8960423993 -0.0617669225

9c

108

C	-0.74699	-1.22040	5.18824
C	-2.22552	-1.21776	5.59425
C	-2.89356	0.07140	5.10034
C	-2.18385	1.31867	5.64274
C	-0.70625	1.26690	5.24280
C	-0.00000	0.00000	5.73851
H	-2.88446	0.09683	3.99430
H	-2.23618	1.25341	6.74704
H	-0.64894	1.27907	4.14026
H	-0.09815	-0.03422	6.84093
H	-0.67478	-1.21309	4.08485
H	-2.25615	-1.17572	6.70082
H	-3.94384	0.08346	5.41478
C	-2.86847	2.66593	5.26158
H	-0.17957	2.16602	5.59641
C	1.52970	0.00476	5.44196
H	-0.26976	-2.14663	5.53455
C	-2.97595	-2.52769	5.20832
C	-2.37564	3.76803	6.20931
C	-2.54828	3.07412	3.81774
C	-4.39150	2.56140	5.41541
C	2.13418	-1.38174	5.69950
C	1.81979	0.40638	3.99026
C	2.22319	1.00057	6.38159
C	-2.76973	-2.88978	3.73325

C	-2.46359	-3.68321	6.07951
C	-4.48118	-2.37933	5.46857
H	-1.28204	3.87771	6.17863
H	-2.81416	4.73875	5.93155
H	-2.66745	3.54942	7.24813
H	-1.49538	3.37087	3.70426
H	-2.75003	2.25162	3.11317
H	-3.16966	3.93222	3.51765
H	-4.66511	2.15378	6.40174
H	-4.84864	3.55826	5.32322
H	-4.83744	1.91821	4.64258
H	1.82220	-2.11145	4.93810
H	3.23292	-1.32464	5.67369
H	1.83846	-1.76775	6.68819
H	1.23466	-0.19978	3.28001
H	1.58467	1.46492	3.80851
H	2.88599	0.25658	3.75915
H	2.10289	0.69516	7.43254
H	3.30122	1.04742	6.16398
H	1.81955	2.01753	6.27389
H	-1.72450	-3.15895	3.52041
H	-3.39738	-3.75275	3.46093
H	-3.05108	-2.05470	3.07389
H	-1.38666	-3.85704	5.94482
H	-2.64672	-3.48023	7.14610
H	-2.98341	-4.61782	5.81920
H	-4.95614	-1.69626	4.74940
H	-4.97841	-3.35662	5.37521
H	-4.67351	-1.99714	6.48390
C	-2.12823	-1.29641	0.15862
C	-2.91395	-0.07403	-0.33185
C	-2.17372	1.20834	0.06678
C	-0.74716	1.24407	-0.49316
C	0.00000	0.00000	0.00000
C	-0.69688	-1.30600	-0.39335
H	-2.12382	1.26885	1.16971
H	-0.83313	1.17437	-1.59494
H	0.04608	0.04553	1.10220
H	-0.78734	-1.31994	-1.49702
H	-2.09474	-1.29987	1.26451
H	-2.90102	-0.12354	-1.43845
H	-2.73946	2.08438	-0.27691
C	0.02410	2.56680	-0.20362
H	1.03975	0.00593	-0.36023
C	0.13033	-2.57224	-0.01651
H	-2.64482	-2.21248	-0.15140
C	-4.42064	-0.06802	0.06577
C	1.22546	2.66888	-1.15322
C	0.52891	2.61762	1.24392
C	-0.87636	3.78356	-0.45410

C	-0.72322	-3.83897	-0.16124
C	0.65579	-2.49672	1.42292
C	1.32351	-2.69857	-0.97364
C	-4.61901	0.29721	1.54112
C	-5.04614	-1.44700	-0.18449
C	-5.17246	0.94989	-0.80329
H	1.90395	1.80968	-1.05203
H	1.80810	3.57803	-0.93950
H	0.89209	2.71890	-2.20134
H	1.33046	1.88565	1.41876
H	-0.28238	2.41280	1.96091
H	0.93298	3.61637	1.47175
H	-1.36552	3.72116	-1.43939
H	-0.27737	4.70655	-0.43195
H	-1.65897	3.87790	0.31287
H	-1.49783	-3.90133	0.61714
H	-0.08828	-4.73339	-0.07137
H	-1.21993	-3.87420	-1.14405
H	-0.15137	-2.25950	2.13414
H	1.43992	-1.73285	1.52860
H	1.09121	-3.46324	1.72083
H	0.97988	-2.84291	-2.00958
H	1.94635	-3.56393	-0.69981
H	1.96638	-1.80691	-0.94911
H	-4.02718	-0.35836	2.19779
H	-5.67707	0.18191	1.82408
H	-4.33202	1.33917	1.74646
H	-4.68571	-2.19726	0.53424
H	-4.81953	-1.80799	-1.20050
H	-6.14033	-1.38918	-0.08230
H	-4.78370	1.97004	-0.67565
H	-6.23963	0.96785	-0.53441
H	-5.09685	0.68569	-1.86950

9e

132

C	2.25263	6.26910	4.19134
C	2.01060	6.05475	5.68656
C	2.15993	4.58336	6.08102
C	1.23953	3.68613	5.24596
C	1.54475	3.89951	3.75924
C	1.35778	5.35876	3.34872
C	1.27219	2.20693	5.67483
C	2.52085	1.45737	5.19888
C	2.54657	0.00097	5.67331
C	1.27045	-0.70961	5.21044
C	-0.00000	0.00000	5.68941
C	0.01809	1.45663	5.21510
C	3.84193	-0.71529	5.24748
C	4.15299	-1.95939	6.08702
C	5.49980	-2.57280	5.69641
C	5.56382	-2.89519	4.20238
C	5.22982	-1.66647	3.35513
C	3.87472	-1.09061	3.76182
C	-1.27220	-0.76667	5.28096
C	-2.49328	-0.41518	6.13828
C	-3.70277	-1.27577	5.76559
C	-4.03575	-1.17024	4.27632
C	-2.81701	-1.49531	3.41131
C	-1.63519	-0.60897	3.80022
H	2.53508	0.01631	6.77977
H	3.43416	1.97544	5.53680
H	2.54181	1.46418	4.09438
H	1.27655	2.19099	6.78130
H	-0.88249	1.98828	5.56575
H	-0.01298	1.47337	4.11087
H	0.02877	0.01084	6.79561
H	1.26440	-1.75689	5.55642
H	1.25828	-0.73965	4.10608
H	4.14947	-1.70039	7.15787
H	3.35491	-2.70870	5.93881
H	3.61784	-0.22002	3.13787
H	3.10355	-1.85761	3.57350
H	1.94011	4.45319	7.15273
H	3.20868	4.27004	5.93144
H	0.92157	3.23659	3.13848
H	2.59560	3.61967	3.56990
H	-2.25136	-0.54142	7.20567
H	-2.74602	0.65045	5.99367
H	-0.76167	-0.82186	3.16377
H	-1.91684	0.44207	3.61475
H	5.69485	-3.47806	6.29122
H	5.22828	-1.92593	2.28457
H	4.83403	-3.69199	3.97443
H	6.55617	-3.29123	3.93841
H	-4.57570	-0.99276	6.37324
H	-3.05652	-1.36475	2.34410
H	-4.36430	-0.13988	4.05302
H	-4.87852	-1.83175	4.02446
H	2.69574	6.68092	6.27803
H	1.58006	5.48517	2.27725
H	3.30810	6.04037	3.96090
H	2.09325	7.32507	3.92528
H	0.20570	4.04175	5.42111
H	0.98741	6.38668	5.93497
H	0.30095	5.64678	3.49017
H	4.66887	0.00013	5.42167
H	6.30181	-1.85531	5.94288
H	6.01128	-0.89854	3.49472

H	-1.06201	-1.84007	5.45295
H	-3.47799	-2.32891	6.00882
H	-2.54043	-2.55589	3.54741
C	6.56126	2.62365	0.91515
C	6.22268	2.73653	-0.57231
C	5.01237	1.87715	-0.94510
C	3.79416	2.22295	-0.08137
C	4.16317	2.05747	1.39701
C	5.34540	2.94311	1.78626
C	2.52141	1.45678	-0.48926
C	2.50289	0.00003	-0.01519
C	1.24876	-0.74974	-0.47563
C	0.00000	0.00000	0.00000
C	-0.02484	1.45644	-0.47431
C	1.25114	2.16664	-0.01021
C	1.28079	-2.22925	-0.04763
C	0.36678	-3.12694	-0.88921
C	0.51518	-4.59846	-0.49485
C	0.26435	-4.81439	0.99870
C	1.15269	-3.90346	1.84756
C	0.96590	-2.44427	1.43681
C	-1.32058	2.17325	-0.05044
C	-1.63295	3.41365	-0.89500
C	-2.97964	4.02828	-0.50591
C	-3.04281	4.35682	0.98681
C	-2.70762	3.13177	1.83888
C	-1.35246	2.55514	1.43352
H	1.24484	-0.73307	-1.58209
H	3.40348	-0.53164	-0.36587
H	2.53369	-0.01741	1.08909
H	2.49219	1.44592	-1.59544
H	1.25750	3.21403	-0.35592
H	1.26315	2.19645	1.09424
H	-0.01184	1.44097	-1.58075
H	-0.91314	-0.51786	-0.33878
H	-0.02198	-0.00697	1.10453
H	0.59344	-2.99590	-1.95937
H	-0.68322	-2.81458	-0.74629
H	1.58302	-1.78018	2.06253
H	-0.08694	-2.16690	1.61854
H	4.76640	2.00918	-2.01085
H	5.26613	0.81082	-0.80742
H	3.29194	2.26520	2.03841
H	4.44733	1.00579	1.57483
H	-1.63035	3.15000	-1.96472
H	-0.83485	4.16375	-0.75081
H	-1.09379	1.68784	2.06142
H	-0.58189	3.32382	1.61719
H	-0.16563	-5.22493	-1.09099
H	0.92388	-4.03095	2.91752
H	-0.79282	-4.58735	1.22292
H	0.42361	-5.87036	1.26481
H	-3.17541	4.93099	-1.10435
H	-2.70542	3.39565	2.90835
H	-2.31324	5.15489	1.21102
H	-4.03517	4.75347	1.24980
H	7.09355	2.45731	-1.18465
H	5.58889	2.80723	2.85190
H	6.89158	1.59249	1.13200
H	7.40433	3.28466	1.16729
H	3.58229	3.29702	-0.24689
H	5.99628	3.79076	-0.80927
H	5.06726	4.00409	1.65666
H	2.31613	-2.58355	-0.21644
H	1.54022	-4.92906	-0.73730
H	2.21082	-4.18980	1.71251
H	-2.14713	1.45656	-0.22109
H	-3.78157	3.30950	-0.74886
H	-3.48875	2.36286	1.70301

9f

96

0 1

C	-1.344687	-0.636021	4.939892
C	-1.283477	0.831093	5.391217
C	0.047496	1.446196	4.966659
C	1.269105	0.652330	5.453655
C	1.154257	-0.800642	4.999935
C	-0.161530	-1.469827	5.424884
H	-2.292102	-1.098854	5.256412
H	-1.358713	-0.641490	3.837271
H	-1.341640	0.853342	6.495407
H	0.092342	1.477750	3.865257
H	0.107787	2.493124	5.301750
H	1.273259	0.672953	6.559401
H	1.202569	-0.837188	3.898845
H	2.017426	-1.381692	5.359604
H	-0.188216	-1.503302	6.530000
C	-0.192577	-2.901468	4.908879
C	-1.114309	-3.345163	3.952125
H	-1.900970	-2.684818	3.581174
C	-1.040789	-4.641425	3.435421
H	-1.766713	-4.952683	2.681869
C	-0.055827	-5.522229	3.873649
H	0.001974	-6.532765	3.466652
C	0.850230	-5.102999	4.850009
H	1.616751	-5.788499	5.216365
C	0.778831	-3.808858	5.357803
H	1.496789	-3.489863	6.117836
C	2.541662	1.329209	4.964919

C	2.826026	2.634435	5.393825
H	2.164672	3.113711	6.120285
C	3.928073	3.332290	4.907940
H	4.125583	4.347239	5.258009
C	4.777550	2.735369	3.974151
H	5.637498	3.281701	3.584050
C	4.522627	1.431874	3.556936
H	5.181242	0.942119	2.837187
C	3.419483	0.731223	4.051834
H	3.253731	-0.288788	3.698600
C	-2.487615	1.581771	4.840495
C	-2.375338	2.617453	3.904298
H	-1.396875	2.974592	3.576212
C	-3.514675	3.209872	3.353140
H	-3.393310	4.006637	2.616834
C	-4.785469	2.789486	3.735880
H	-5.673658	3.250882	3.301802
C	-4.911824	1.779103	4.691453
H	-5.901754	1.451522	5.014563
C	-3.775066	1.186248	5.233707
H	-3.886144	0.392569	5.977053
C	-1.155164	0.797862	1.100181
C	0.160578	1.467056	0.675106
C	1.343737	0.633121	1.159888
C	1.282449	-0.834056	0.708759
C	-0.048589	-1.449036	1.133371
C	-1.270063	-0.655064	0.646241
H	-2.018462	1.378894	0.740848
H	-1.203178	0.833935	2.201322
H	0.187075	1.500764	-0.430007
H	1.357900	0.638716	2.262529
H	2.291160	1.095814	0.843206
H	1.340559	-0.856498	-0.395420
H	-0.093414	-1.480497	2.234768
H	-0.108979	-2.495985	0.798344
H	-1.274103	-0.675661	-0.459500
C	-2.542754	-1.331831	1.134790
C	-3.420351	-0.734107	2.048225
H	-3.254391	0.285699	2.401959
C	-4.523631	-1.434758	2.542860
H	-5.182104	-0.945136	3.262831
C	-4.778854	-2.738007	2.125093
H	-5.638855	-3.284361	2.515040
C	-3.929545	-3.334687	1.190983
H	-4.127252	-4.349448	0.840484
C	-2.827416	-2.636823	0.705329
H	-2.166212	-3.115888	-0.021412
C	2.486547	-1.584634	1.259691
C	3.774076	-1.189194	0.866628
H	3.885298	-0.395534	0.123285
C	4.910746	-1.782109	1.408981
H	5.900710	-1.454568	1.085933
C	4.784252	-2.792506	2.364535
H	5.672357	-3.253966	2.798710
C	3.513390	-3.212802	2.747125
H	3.391912	-4.009545	3.483428
C	2.374129	-2.620305	2.195846
H	1.395635	-2.977368	2.523882
C	0.191756	2.898625	1.191351
C	1.114280	3.342341	2.147313
H	1.901293	2.682056	2.517639
C	1.041200	4.638629	2.664037
H	1.767777	4.949896	3.416955
C	0.055878	5.519434	2.226636
H	-0.001554	6.529981	2.633656
C	-0.851030	5.100165	1.251070
H	-1.617869	5.785640	0.885327
C	-0.780033	3.806019	0.743233
H	-1.498608	3.487061	-0.016228

9f stuck

96

0 1

C -1.2570382253 -0.7242632962 5.6191618301
C -1.2764914549 0.7352260589 6.0901623972
C -0.000135296 1.4536626911 5.6346775226
C 1.2750554632 0.7350261106 6.0931049079
C 1.2564913738 -0.724547148 5.622284572
C -0.0009096985 -1.4721157111 6.0832409769
H -2.1618518512 -1.2479416314 5.9643175953
H -1.2857512416 -0.7378865183 4.5149243024
H -1.2863463482 0.7326591048 7.1943380698
H 0.0011058261 1.5013001433 4.5311647006
H -0.0004574507 2.4943563729 5.9939747872
H 1.2824982243 0.7326678802 7.1973001539
H 1.288088499 -0.7381603856 4.5181556341
H 2.1603634598 -1.248298881 5.9697902561
H -0.0022939336 -1.4802384811 7.1874462804
C -0.0008392524 -2.9112675783 5.6129079471
C 0.0029202412 -3.2141545109 4.2449829634
H 0.0062465899 -2.4065425223 3.5093608262
C 0.0027073256 -4.5333476743 3.7999660204
H 0.005840257 -4.7413319881 2.728647867
C -0.0014422972 -5.5807528152 4.7218762854
H -0.0016474743 -6.6156899215 4.3768944394
C -0.0053126254 -5.2950938709 6.0856080759
H -0.008569444 -6.1073392596 6.8144026744
C -0.0049810876 -3.9706511553 6.5247897837
H -0.0079726469 -3.7538189204 7.5956728914
C 2.5206022193 1.4583610786 5.6258995092

C 3.4338125112 1.9921769784 6.5395420858
H 3.2446012573 1.8828415386 7.6100815529
C 4.578421131 2.659991899 6.1023657169
H 5.2786324841 3.0694110379 6.8324211782
C 4.8273996985 2.8043022081 4.7391047586
H 5.721742358 3.3262894859 4.3958731925
C 3.92450822 2.2764116465 3.8154411381
H 4.1060745618 2.3816683977 2.744501652
C 2.7846328529 1.6110522323 4.2583571666
H 2.0880364779 1.2037762637 3.5218911511
C -2.520877072 1.458887375 5.6203855912
C -2.7818835157 1.6117637002 4.2522972853
H -2.0836435111 1.2044588721 3.5174265387
C -3.9207541677 2.2771796834 3.8069131701
H -4.0999996118 2.3824293585 2.7355799249
C -4.8255785376 2.8051293758 4.7286527231
H -5.7191252979 3.3272198662 4.3835104938
C -4.5795432931 2.6607706947 6.0924442751
H -5.2812903581 3.0702417803 6.8209952186
C -3.4359811459 1.9927724781 6.5320827976
H -3.2491407304 1.8833215834 7.6030281965
C -1.2556492495 0.7232220937 0.4766992501
C 0.0029850171 1.4694023344 0.0166181393
C 1.2583604906 0.7192177025 0.4791933289
C 1.2749027972 -0.7404214318 0.0086055284
C -0.0021244253 -1.4563922133 0.4660901977
C -1.2761009313 -0.7365258627 0.0064265212
H -2.158569712 1.2482420098 0.1286395077
H -1.2879954601 0.736809403 1.580782962
H 0.0041203371 1.4795324524 -1.0875496548
H 1.2885563048 0.7327997822 1.5833378903
H 2.1636139206 1.241386435 0.1329084564
H 1.283222707 -0.73834442 -1.0955848395
H -0.0030322451 -1.5014844328 1.5697614223
H -0.0033938792 -2.4977693149 0.1088282429
H -1.282756179 -0.7347714761 -1.0977762748
C -2.5235382689 -1.4568591293 0.4731391774
C -2.7861223816 -1.6135161579 1.8405293698
H -2.0863632435 -1.2130156157 2.5777327367
C -3.9288195069 -2.274584895 2.2826400564
H -4.1093158455 -2.382944115 3.3534483476
C -4.8359020967 -2.7942773326 1.3584465847
H -5.7324067547 -3.3129085504 1.7011269954
C -4.5883075204 -2.6460798893 -0.0046417398
H -5.2917366444 -3.0490914683 -0.7351674715
C -3.4409787535 -1.9824583392 -0.4410450561
H -3.2529309416 -1.8699355301 -1.5114605102
C 2.5187292513 -1.465956493 0.4768912061
C 3.4315594051 -2.0015558012 -0.4361125926
H 3.2432582536 -1.8918928464 -1.506778724
C 4.5745776258 -2.6715885544 0.001806061
H 5.2744718119 -3.0823703629 -0.727788171
C 4.8223605231 -2.8164092743 1.3652314023
H 5.7154303891 -3.3401703397 1.7090713485
C 3.9198901558 -2.2867188841 2.288264232
H 4.1005210783 -2.3923209114 3.3593303301
C 2.7815973423 -1.6191153807 1.844607242
H 2.0855200283 -1.210521198 2.5808298259
C 0.0046806012 2.9075719533 0.4900937146
C 0.0031058616 3.2066702722 1.8587756534
H 0.0005452782 2.3965620771 2.5916190577
C 0.004591187 4.5245737886 2.3076182496
H 0.0034199366 4.7294706338 3.379542521
C 0.0075612457 5.5744903832 1.3886094793
H 0.0087351575 6.6084574179 1.7364826957
C 0.0088667211 5.2926221907 0.0240238797
H 0.0111058046 6.1069197937 -0.7024800083
C 0.0073617749 3.9694507446 -0.4189211064
H 0.008440539 3.7555375654 -1.4903975628

9d

180
0 1
C -0.824284665 1.171471601 5.5318831873
C -1.481005561 -0.0793609294 4.9355237277
C -0.8132553165 -1.3726341796 5.421879948
C 0.6820768363 -1.3439757774 5.0808082822
C 1.3962936392 -0.0980936017 5.6219156908
C 0.6566218215 1.1553905671 5.1476981978
C -1.5190037205 2.5146423161 5.1605678021
H -0.8993294976 1.0812507299 6.6329570098
H -2.5487911354 -0.1004564659 5.1834714341
H -1.4011609177 -0.0220813603 3.8345706777
H -0.8881313518 -1.3763490598 6.5276011718
C -1.5433517232 -2.6644397217 4.949985878
H 1.1651423058 -2.2395198445 5.4880509548
H 0.8065904872 -1.377300491 3.9829064447
H 1.3206180507 -0.1415787555 6.7258488695
C 2.9176421839 -0.0441516737 5.2892179104
H 1.1650598956 2.0631202464 5.508941301
H 0.7098285866 1.1793903701 4.0463996651
C -1.1276189839 3.0172396069 3.7546030011
C -3.0552130397 2.3800240081 5.2018356356
C -1.1143014748 3.6035346625 6.1777681278
C -1.6374153318 -2.7623109285 3.4162916244
C -0.8081378757 -3.9264790978 5.4523709291
C -2.9809229258 -2.7038311308 5.5147727174
C 3.1857625941 0.4772777409 3.8625400898
C 3.6341155632 0.9016896022 6.2768403042
C 3.5720118362 -1.4361970722 5.4132529201

H -1.3818034598 2.2512613888 3.0018884218
C -1.8271764313 4.3440130602 3.4254159837
H -0.0383125621 3.1762694915 3.7051743286
H -3.3808300601 1.6437136028 4.4498089709
H -3.3654382598 1.9968390254 6.1903501029
C -3.7461888187 3.7169037444 4.9036795199
H -0.0191270536 3.729420133 6.1772186996
C -1.7879911154 4.9452301124 5.8580843779
H -1.4009084776 3.2755341588 7.191657947
H -0.6236409001 -2.7503878266 2.9806306374
C -2.3677160616 -4.0418705327 2.98101719
H -2.1739075323 -1.8848546716 3.0176436454
H 0.2093636999 -3.9592687631 5.0327236454
H -0.7065706649 -3.8803031655 6.5511703879
C -1.5428794698 -5.2090291696 5.0370845267
H -3.5527581071 -1.8339418647 5.1560422085
C -3.7173086144 -3.9819273956 5.0903432383
H -2.942104785 -2.6357541929 6.616004999
H 2.7761628809 1.4931270488 3.7529090326
C 4.6897673755 0.5262836738 3.5605238589
H 2.6670314558 -0.1625007395 3.1270975018
H 3.1930377641 1.9102405161 6.217832094
H 3.4737069753 0.5388678975 7.3065762676
C 5.1391370554 0.9796401592 5.9835503637
H 3.1131835859 -2.1269993147 4.6876471613
C 5.0806122191 -1.3742576334 5.1394165053
H 3.3804764701 -1.8462087889 6.4209614492
C -1.388340225 5.4019917572 4.4474894895
C -3.3109069997 4.773034878 5.9266675833
C -3.3497280255 4.1724187173 3.4928572039
H -1.534929839 4.6639165235 2.4112947519
H -4.8379832754 3.5780801957 4.9537906735
H -1.4664987871 5.6979654076 6.5949095254
H -0.2966273535 5.5485402772 4.3926613054
H -1.8595402989 6.3715405633 4.2168010495
H -3.6095819442 4.4622251945 6.941294674
H -3.8123857317 5.7322846211 5.717709615
H -3.679911314 3.426273115 2.7509188262
H -3.8493102133 5.1231800004 3.244244747
C 5.7545697947 -0.4179312561 6.1309152007
C 5.3624876865 1.4864297178 4.5513525068
C 5.3030839726 -0.8716348832 3.7065684457
H 4.8379184404 0.8921387122 2.5305068957
H 5.6131743593 1.670734762 6.6981189822
H 5.5084998925 -2.3835699751 5.2476183066
H 5.6164500353 -0.7838025521 7.1616224736
H 6.8398159296 -0.3779436975 5.9411379575
H 4.9413811134 2.49931177 4.437844355
H 6.4421735163 1.556403488 4.3399190336
H 4.8353034006 -1.5649517042 2.9872873842
H 6.3807509402 -0.8398610853 3.4762723841
C -1.6154821975 -5.2717670343 3.504559802
C -3.789480279 -4.0403993876 3.5573618983
C -2.9620402232 -5.2078772701 5.6162926591
H -2.4124430745 -4.0748173715 1.8790306179
H -0.9873157543 -6.0810791641 5.4161329031
H -4.7365686459 -3.9665489485 5.5071145046
H -0.6002113696 -5.303726287 3.0761709659
H -2.1304356859 -6.1931357142 3.1865111838
H -4.3530554876 -3.1754734724 3.1699894799
H -4.3278522092 -4.9476943099 3.2377273151
H -2.9209658803 -5.1815034726 6.7175986123
H -3.4899935269 -6.1325054216 5.3306088799
C 0.6888720221 1.2237149986 -0.530700252
C -0.7493982542 1.2320267848 -0.0021863252
C -1.5078157802 -0.0697813001 -0.2850007051
C -0.7337868053 -1.2551643415 0.3064758217
C 0.6751336231 -1.3334540682 -0.2993739045
C 1.4055227068 -0.0220109209 0.0029053311
C 1.4736962393 2.5342020679 -0.2205311175
H 0.6341843048 1.1433202215 -1.6336583356
H -1.3070523661 2.0761428589 -0.430923941
H -0.7131810771 1.3939586904 1.0902843869
H -1.4945670239 -0.2220207985 -1.3819400861
C -3.0157511718 0.0214441229 0.102389287
H -1.2759364205 -2.1873477956 0.1061906238
H -0.6639339068 -1.1479848308 1.4052193715
H 0.5339452364 -1.4031944845 -1.3953554474
C 1.5116920013 -2.5869109737 0.0988849683
H 2.441887228 -0.0576971445 -0.3688855354
H 1.4655800903 0.0685689959 1.1000199218
C 1.9964003702 2.5722129669 1.2306393309
C 0.5892157157 3.7804287784 -0.4374334526
C 2.6927646787 2.6547346672 -1.1601328485
C -3.2465035552 0.7639813913 1.4351737262
C -3.6610756283 -1.3749997506 0.2209272411
C -3.7744512793 0.7938050935 -1.0001173551
C 2.2512639278 -2.4105113379 1.4417538524
C 2.5758735227 -2.8513036441 -0.9892140118
C 0.6304571313 -3.8489637212 0.2076409521
H 1.1545047659 2.461126075 1.9373894154
C 2.7613201263 3.8719440571 1.5136067467
H 2.67717592 1.7249412912 1.4038093723
H -0.2694864294 3.7516164883 0.2516623096
H 0.1841650377 3.7706683502 -1.4651252296
C 1.370097879 5.0758580468 -0.1811509345
H 3.3527259484 1.7810068366 -1.032866791
C 3.4847245446 3.941476022 -0.8849103669
H 2.345125074 2.6475787313 -2.207459714
H -2.7080956684 0.2490901359 2.2503301693

C -4.7417821398 0.8621036759 1.7684114707
H -2.8330887112 1.7827268719 1.36762968
H -3.1687643892 -1.9465951832 1.0238680407
H -3.5050127853 -1.9351562973 -0.7182107219
C -5.1597736301 -1.2747012455 0.536568696
H -3.3397160523 1.8003404104 -1.1184590144
C -5.27030792 0.9106187497 -0.6779624178
H -3.6415375922 0.270214131 -1.9621867393
H 2.9242494522 -1.5400397072 1.3855661333
C 3.0848613789 -3.652406524 1.7873404263
H 1.5221574101 -2.2055621171 2.2453900698
H 3.22529554 -1.9667930496 -1.0989931518
H 2.0700855069 -3.0016617661 -1.9581978202
C 3.4281430713 -4.0830698458 -0.6546059167
H -0.1208895606 -3.7134140473 1.0025384558
C 1.4695797189 -5.0922434774 0.5326715992
H 0.0784612783 -3.9960207904 -0.7377748682
C 3.970914001 3.9492962406 0.5714952743
C 2.5772849852 5.1551423987 -1.123064048
C 1.8492338776 5.0818124417 1.2770248345
H 3.1071547926 3.8647167013 2.5608431002
H 0.7064598544 5.9374233515 -0.3555223124
H 4.3507295322 3.9867581492 -1.5638130423
H 4.6420644166 3.0929764591 0.7515818593
H 4.550764908 4.8654775667 0.7708402163
H 2.2370135234 5.1720769488 -2.1715036615
H 3.1364376203 6.0886367396 -0.9460219456
H 0.9816708128 5.0385895693 1.9578070397
H 2.3915391101 6.016048534 1.497193992
C 2.5188634741 -5.3160323721 -0.5636461057
C 4.1355882728 -3.8707047142 0.6907916696
C 2.1744761588 -4.8810306639 1.8792062978
H 3.5876148724 -3.4935537923 2.755576115
H 4.1775743357 -4.2330283753 -1.4475065956
H 0.8050115648 -5.9687327645 0.59638006
H 2.0215885894 -5.4896308554 -1.5320074028
H 3.117316961 -6.2139302959 -0.3379934745
H 4.8062812748 -2.9970197598 0.6330129244
H 4.7601735564 -4.7462117546 0.9326898959
H 1.4298292166 -4.7304192102 2.67889565
H 2.7639293936 -5.773627484 2.1457101795
C -5.341960543 -0.5437448166 1.8736372202
C -5.4531061497 1.6448209155 0.6570224154
C -5.8759159959 -0.4955198616 -0.5738647584
H -4.8666961201 1.3898196846 2.7283609734
H -5.5809356569 -2.2901503455 0.6098569564
H -5.7725720843 1.4719620798 -1.4813774055
H -4.8398909403 -1.1010350593 2.6827692905
H -6.4116997285 -0.4838902713 2.1330715217
H -5.0369459239 2.664041777 0.5892104539
H -6.5256968262 1.7452014861 0.890713749
H -5.7683969808 -1.0243116574 -1.5350949323
H -6.9545875869 -0.4296023465 -0.3560681596
